# Efficacy and Safety of Azithromycin-Chloroquine versus Sulfadoxine-Pyrimethamine for Intermittent Preventive Treatment of *Plasmodium falciparum* Malaria Infection in Pregnant Women in Africa: An Open-Label, Randomized Trial

**DOI:** 10.1371/journal.pone.0157045

**Published:** 2016-06-21

**Authors:** Joshua Kimani, Kamija Phiri, Steve Kamiza, Stephan Duparc, Ayman Ayoub, Ricardo Rojo, Jeffery Robbins, Russell Orrico, Pol Vandenbroucke

**Affiliations:** 1 College of Health Sciences, University of Nairobi, Nairobi, Kenya; 2 College of Medicine, University of Malawi, Blantyre, Malawi; 3 Medicines for Malaria Venture, Geneva, Switzerland; 4 Pfizer Inc., Sandwich, Kent, United Kingdom; 5 Pfizer Inc., Groton, CT, United States of America; 6 Pfizer Inc., Collegeville, PA, United States of America; 7 Pfizer Inc., New York, NY, United States of America; University of California Los Angeles, UNITED STATES

## Abstract

**Background:**

The World Health Organization recommends intermittent preventive treatment in pregnancy (IPTp) with sulfadoxine-pyrimethamine (SP) in African regions with moderate to high malaria transmission. However, growing resistance to SP threatens the effectiveness of IPTp-SP, and alternative drugs are needed. This study tested the efficacy, tolerability, and safety of a fixed-dose combination azithromycin-chloroquine (AZCQ; 250 mg AZ/155 mg CQ base) for IPTp relative to IPTp-SP.

**Methods and Findings:**

A randomized, Phase 3, open-label, multi-center study was conducted in sub-Saharan Africa (Benin, Kenya, Malawi, Tanzania, and Uganda) between October 2010 and November 2013. Pregnant women received 3 IPTp courses with AZCQ (each course: 1,000/620 mg AZCQ QD for 3 days) or SP (each course 1,500/75 mg SP QD for 1 day) at 4- to 8-week intervals during the second and third trimester. Long-lasting insecticide-treated bednets were also provided at enrollment. Study participants were followed up until day 28 post delivery (time window: day 28–42). The primary endpoint was the proportion of participants with sub-optimal pregnancy outcomes (a composite endpoint comprising live-borne neonates with low birth weight [LBW, <2,500 g], premature birth [<37 weeks], still birth [>28 weeks], abortion [≤28 weeks], lost to follow-up prior to observation of pregnancy outcome, or missing birth weight). The study was terminated early after recruitment of 2,891 of the planned 5,044 participants, due to futility observed in a pre-specified 35% interim analysis. In the final intent-to-treat dataset, 378/1,445 (26.2%) participants in the AZCQ and 342/1,445 (23.7%) in the SP group had sub-optimal pregnancy outcomes, with an estimated risk ratio (RR) of 1.11 (95% CI: 0.97, 1.25; *p* = 0.12). There was no significant difference in the incidence of LBW between treatment groups (57/1138 [5.0%] in the AZCQ group, 68/1188 [5.7%] in the SP group, RR 0.87 [95% CI: 0.62, 1.23]; *p* = 0.44). IPTp-AZCQ was less well-tolerated in mothers than IPTp-SP. Occurrences of congenital anomalies, deaths, and serious adverse events were comparable in neonates for both groups. Limitations included the open-label design and early study termination.

**Conclusions:**

IPTp-AZCQ was not superior to IPTp-SP in this study and alternatives for IPTp-SP remain to be identified. The proportions of sub-optimal pregnancy outcomes and LBW were lower than expected, which may be linked to insecticide-treated bednet use throughout the study. Reduced incidences of symptomatic malaria infection and peripheral parasitemia in the AZCQ group relative to SP suggest that AZCQ warrants further investigation as an alternative treatment of uncomplicated malaria.

**Trial Registration:**

ClinicalTrials.gov (NCT01103063).

## Introduction

Malaria in pregnancy is one of the leading preventable causes of maternal, perinatal, and neonatal morbidity and mortality in sub-Saharan Africa [1]. Annually, an estimated 30 million pregnancies are at risk of *Plasmodium falciparum* infection in stable transmission areas in sub-Saharan countries [1]. The risk of acquiring malaria infection during pregnancy and suffering adverse consequences depend on the level of acquired anti-malarial immunity and the risk is high in adolescent women [2], in first and second pregnancies [1], and in those co-infected with human immunodeficiency virus (HIV) [3]. *P*. *falciparum* infection during pregnancy increases the risk of spontaneous abortion, stillbirth, and prematurity, particularly if it results in acute febrile illness [4]. Furthermore, peripheral and placental *P*. *falciparum* parasitemia can lead to severe maternal anemia, intrauterine growth retardation, preterm delivery, and low birth weight (LBW) [4], which is a crucial indicator of neonatal/infant mortality [5–7], and impaired cognitive development [8].

Important progress in the control of malaria in pregnancy has been made by vector control with long-lasting insecticide-treated bednets (LLIN) and the use of intermittent preventive treatment during pregnancy (IPTp) in areas with moderate to high malaria transmission. IPTp is the periodic, presumptive administration of curative courses of effective antimalarial medication to pregnant women, with the dual goal of clearing any existing peripheral and placental malaria infections and preventing new infections during pregnancy. In 2013, IPTp had been adopted by 36 sub-Saharan African countries and in Papua New Guinea [9]. The World Health Organization (WHO) currently recommends IPTp with sulfadoxine-pyrimethamine (IPTp-SP) in areas with moderate to high malaria transmission in Africa at each scheduled antenatal care (ANC) visit, starting as early as possible in the second trimester until the time of delivery, with doses given at least 1 month apart, so that women receive at least three doses of SP during pregnancy [10].

IPTp-SP was proven to be efficacious in reducing maternal malaria episodes, maternal anemia, placental parasitemia, occurrence of LBW, and neonatal mortality [11,12]. From existing data, three or more courses of IPTp-SP achieve the greatest benefits [12]. However, rapid spread of *P*. *falciparum* resistance to SP [13–15] could reduce the impact of IPTp-SP. So, well-tolerated, efficacious, affordable alternatives to SP are desired for use in IPTp.

A fixed-dose combination therapy with the widely used macrolide antibiotic, azithromycin (AZ), and the former first-line antimalarial treatment, chloroquine (CQ) [16] was evaluated as a possible alternative IPTp drug to SP. As individual agents, AZ and CQ each have a long history of use; offer extensive safety records in adults, children, and pregnant women; and are considered safe in all trimesters of human pregnancy [17]. AZ and CQ exert additive or synergistic activity against CQ-resistant *P*. *falciparum* strains *in vitro* [18,19] and *in vivo* [20]. Moreover, co-administration of AZ and CQ demonstrated 98% and 100% efficacy in the treatment of acute uncomplicated *P*. *falciparum* malaria in non-pregnant adults in two multi-country Phase 3 clinical studies in sub-Saharan Africa [21].

A Phase 3 clinical trial (clinicaltrials.gov identifier: NCT01103063, S1 Text) was undertaken to evaluate the efficacy and safety of IPTp with AZCQ versus IPTp-SP in pregnant women in East and Southern African countries, where SP is the current standard of care and antifolate resistance in *P*. *falciparum* was evident. The study tested the hypothesis that IPTp-AZCQ would reduce the incidence of sub-optimal pregnancy outcomes.

## Methods

### Study Design

This was a multi-center, Phase 3, open-label, randomized, clinical trial that compared the effectiveness of three IPTp courses of AZCQ or SP in pregnant women in five countries in sub-Saharan Africa where antifolate resistance of *P*. *falciparum* to SP was established. Women of all gravidities were enrolled during the second trimester of pregnancy and allocated to receive three IPTp courses of AZCQ or SP during ANC visits at 4- to 8-week intervals. They were then followed up at week 36 to 38 of gestation, at delivery (or within 2 days of study participant reporting home delivery), and on day 28 post delivery (time window: day 28 to 42).

As the study was conducted in areas where *P*. *falciparum* resistance to SP was documented to reduce the protective efficacy of IPTp-SP [8,13,22–24], a superiority design was chosen. A composite primary endpoint was chosen so that all possible pregnancy outcomes that are potentially affected by uncontrolled parasitemia would be included in the analysis. The rationale for selection of endpoints and design features has been described in detail elsewhere [25].

The study was approved by the London School of Hygiene and Tropical Medicine Ethics Committee; the Comité National d’Ethique pour la Recherche en Santé in Cotonou, Benin; the Kenyatta National Hospital—University of Nairobi Ethics Review Committee in Nairobi, Kenya; the College of Medicine Research and Ethics Committee in Blantyre, Malawi; the Medical Research Coordinating Committee in Dar es Salaam, Tanzania; and the Uganda National Council of Science and Technology, the School of Medicine Research and Ethics Committee of Makerere University, and the Mulago Hospital Research and Ethics Committee in Kampala, Uganda. The study was overseen by an independent External Data Monitoring Committee (EDMC) and conducted in accordance with the Declaration of Helsinki on Ethical Principles for Medical Research Involving Human Study Participants; the International Conference on Harmonisation-Good Clinical Practice (ICH-GCP) standards; and local regulatory and legal requirements. Participants (or a legally acceptable representative if the participant was <18 years of age) were to provide written informed consent before enrollment and any study procedures took place. All study participants <18 years of age were to provide assent.

### Study Sites and Participants

The study was conducted between October 2010 and November 2013 at six sites: (1) the Centre de Sante d'Ahouansori Agué and Hôpital Bethesda in Cotonou, Benin; (2) the Siaya District Hospital, in Siaya, Kenya; (3) the Zomba Central Hospital in Zomba, Malawi; (4) The Teule Hospital in Muheza, Tanga, Tanzania; (5) the National Institute for Medical Research (Mwanza Centre)/Nyamagana District Hospital, in Mwanza, Tanzania; and (6) the Mulanda Health Centre IV, in Kampala, Uganda. Pregnant women of all gravidities were eligible if they carried a single fetus of 14 to 26 weeks of gestation (defined by pelvic ultrasound examination at screening), were 16 to 35 years of age, and willing and able to comply with all study procedures and to attend all scheduled follow-up visits. Women presenting at enrollment with clinical symptoms of malaria, severe anemia (hemoglobin <8 g/dL), any condition requiring hospitalization, obstetric complications increasing the risk of sub-optimal pregnancy outcome (e.g., presence of congenital anomalies, placenta previa, or abruption), evidence of severe concomitant infection, or who had taken antimalarial drugs within the past 4 weeks were excluded from enrollment (S1 Table).

### Study Interventions

Study participants were randomly assigned (1:1) to the IPTp-AZCQ or the IPTp-SP regimen using computer-generated randomization cards provided by the sponsor to the investigators. Treatment group assignment remained concealed until the investigator confirmed the study participant met all eligibility criteria. Randomization was stratified according to gravidity into two approximately equal-sized strata (‘primi- and secundigravidae’ and ‘other gravidae’).

Participants in both regimens received three IPTp courses: the first course between 14 and 26 weeks of gestation and the subsequent courses at 4- to 8-week intervals, with the third course administered prior to or during the 36th week of gestation. In addition, all participants also received a LLIN on day 0 of the study, and the installation of these nets was verified by fieldworkers on day 1 (AZCQ regimen) or day 2 (SP regimen).

For the AZCQ combination, we used a fixed-dose tablet formulation of AZCQ 250/155 mg [26]; each IPTp treatment course consisted of a 3-day course of AZCQ 1,000/620 mg per day administered orally once daily on days 0, 1, and 2. For the SP regimen, we used fixed-dose tablets of sulfadoxine 500 mg plus pyrimethamine 25 mg supplied as Fansidar^®^ (Roche); each treatment course consisted of a single dose of sulfadoxine 1,500 mg plus pyrimethamine 75 mg administered orally once on day 0. All study drug doses were administered under direct observation as open-label therapy. Administration of all SP doses and of the first dose of each 3-day AZCQ course was supervised by the investigators during ANC visits, and the second and third doses of each AZCQ treatment course were taken at home under supervision by fieldworkers.

### Outcomes

The composite primary efficacy endpoint was defined as the proportion of participants with a sub-optimal pregnancy outcome comprising: live-born neonate with LBW (defined as <2,500 g), premature birth (delivery before 37 weeks of gestation), still birth (pregnancy loss after 28 weeks of gestation), abortion (pregnancy loss before completion of 28 weeks of gestation), loss to follow-up prior to termination of pregnancy or delivery, and missing birth weight.

Key secondary endpoints included the incidences of: LBW for live-born neonates, sub-optimal pregnancy outcome when including neonatal death and congenital malformation in addition to the six outcomes constituting the primary endpoint, maternal anemia (defined as hemoglobin <11 g/dL) and severe maternal anemia (defined as hemoglobin <8 g/dL) at weeks 36 to 38 of gestation, placental parasitemia at delivery, and of placental malaria infection as detected by histology at delivery; as well as, the number of episodes of curable sexually transmitted infections (STIs) with *Treponema pallidum*, *Neisseria gonorrhoeae*, and *Chlamydia trachomatis* per study participant (based on clinical presentation at any time between the first IPTp dose and delivery or laboratory results for specimens collected at weeks 36 to 38 of gestation).

Other secondary efficacy endpoints were the incidences of: premature birth, still birth, congenital abnormalities in neonates (detected at birth); perinatal or neonatal deaths; study participants requiring additional treatment for malaria during the study period following first IPTp dose (diagnosed based on clinical presentation or laboratory test results); peripheral parasitemia at weeks 36 to 38 of gestation and at delivery; cord blood parasitemia at delivery; STIs with *T*. *pallidum*, *N*. *gonorrhoeae*, or *C*. *trachomatis* following the first IPTp dose (diagnosed based on clinical presentation before, or on laboratory test results between 36 to 38 weeks of gestation); positive laboratory test results for *C*. *trachomatis*, *N*. *gonorrhoeae*, *T*. *pallidum*, *Trichomonas vaginalis*, and bacterial vaginosis at 36 to 38 weeks of gestation; ophthalmia neonatorum in the neonate; bacterial infections including pneumonia and other lower respiratory tract infections between the first IPTp dose and delivery; pre-eclampsia between week 20 and delivery (diagnosed based on high blood pressure [systolic ≥140 mmHg or diastolic ≥90 mmHg in two separate measurements taken ≥4 hours apart] and proteinuria [≥300 mg protein in 24-hour urine collection]); and of nasopharyngeal swabs positive for macrolide-resistant and penicillin-resistant *Streptococcus pneumoniae* at baseline, at day 28 post delivery, and about 6 months following the last IPTp course (tested in ~600 study participants per treatment group); as well as the hemoglobin concentration at weeks 36 to 38 of gestation; the birth weight of live-born neonates; and the number of episodes of symptomatic malaria per study participant between the first IPTp dose and delivery.

Safety endpoints included observed and spontaneously reported adverse events; vital signs; physical examination; laboratory tests (including tests for anemia, glycosuria, and proteinuria); routine obstetric checkup; adverse pregnancy outcomes for mothers; and the general physical examination of neonates through day 28 post delivery (time window: day 28 to 42).

### Procedures

The training of investigators, site teams, and central laboratories as well as compliance and study monitoring have been described previously [25]. The study visits and procedures are outlined in S1 Fig.

Participants with clinical episodes of malaria (defined as fever [oral temperature >37.5°C] and confirmation of malaria by rapid diagnostic test or light microscopy) received standard antimalarial treatment according to the local care guidelines, and continued follow-up. Study participants diagnosed with anemia received standard treatment according to local ANC guidelines.

To investigate whether azithromycin exposure induced emergence of macrolide-resistant pneumococci, nasopharyngeal swabs were collected from a subset of participants (target: about 600 study participants per treatment arm) at baseline, day 28 post delivery, and 6 months after the last IPTp dose, and the sensitivity of isolated *S*. *pneumonia* to azithromycin, erythromycin, and penicillin was determined.

Serious adverse events (SAEs) for mothers or neonates, which were observed or volunteered between signing of informed consent and day 28 to 42 post delivery, including 39 days after the last administration of investigational product, or the last study visit (whichever was later), were recorded and coded using the Medical Dictionary for Regulatory Activities (MedDRA, version 17.0), regardless of suspected causal relationship to study treatment. Adverse events (AEs) were recorded from the time the study participant had taken at least one dose of investigational product through to the last study visit. Patients were evaluated and questioned for AEs at each study visit. Severity and causality of AEs were assessed by the site investigator, with events considered ‘mild’, ‘moderate’, or ‘severe’ if there was no, some, or significant interference with the study participant’s usual function, respectively, and ‘treatment-related’ if there was a reasonable possibility that study treatment had contributed to or caused the event. AEs were classified as ‘serious’ if they were fatal or life-threatening; required inpatient hospitalization or prolongation of existing hospitalization; resulted in significant disability/incapacity; or were a congenital anomaly/birth defect.

Every effort was made to document reasons for discontinuation and pregnancy outcomes for study participants who decided to withdraw from the study. If consent for disclosure of future information was also withdrawn, no further evaluations were performed; but all data collected up to the point of withdrawal remained in the database. In the event of safety concerns or failure to cooperate with study procedures, study participants were discontinued from study drug but not the study per se. All affected participants received standard ANC as per local guidelines, and were followed up regularly.

### Laboratory Procedures

Hemoglobin concentrations were quantified through finger prick or peripheral blood samples using HemoCue^TM^. The presence of peripheral, placental, and cord blood *P*. *falciparum* parasitemia was tested by microscopy using standard Giemsa-stained blood smears (thick and thin) at weeks 36 to 38 of gestation and at hospital delivery. Smears were read and, when positive, parasite density was counted independently by at least two microscopists at different laboratories, blinded to treatment regimen; discrepant results were reviewed by a third microscopist. The parasite count was expressed as the number of parasites per microliter of blood in a thick smear, standardized to a predetermined white cell count of 8000. A blood slide was considered negative when the examination of 100 high power fields on the thick smear did not show the presence of any *falciparum* parasites. In addition, if smears were microscopically-positive for *P*. *falciparum* at weeks 36 to 38 of gestation, the following *P*. *falciparum* genetic resistance markers were determined using polymerase chain reaction (PCR) assay: CQ resistance markers in the *P*. *falciparum* chloroquine resistance transporter (*pfcrt)* and multidrug resistance 1 (*pfmdr1)* genes, and SP resistance markers in the dihydrofolate (*pfdhfr)* and dihydropteroate synthase (*pfdhps)* genes. For placental histology, a sample of placenta tissue (approximately 2 cm x 2 cm x 1 cm) was collected at birth from the participants who delivered at hospital. Samples were placed in 50 mL 10% neutral buffered formalin, stored at room temperature, and shipped for histology review.

The *T*. *pallidum* blood screening test was conducted using the Rapid Plasma Reagnin (RPR) method at baseline and at weeks 36 to 38. Blood samples (~0.5 ml) were collected and the serum used for the test. The Treponema Pallidum Particle Agglutination Assay (TPPA) was used to confirm infection when RPR was positive. *N*. *gonorrhoeae* and *C*. *trachomatis* tests were performed at weeks 36 to 38. An endocervical swab was collected and PCR assay (Amplicor CT/NG, Roche) used for analysis.

All laboratory tests were standardized among units to enable comparison of test results from different laboratories.

### Statistical Analysis

#### Sample size

EAST software Version 5.1 and simulation were used to design the study and calculate the sample size required to detect a 20% risk reduction in the AZCQ treatment group relative to SP, for the primary endpoint of sub-optimal pregnancy outcome, with 90% power and a two-sided type I error rate (alpha) of 0.00125. The resulting sample size was also checked to ensure ≥80% power, at the 0.05 2-sided alpha level, for detecting a 23% risk reduction in the key secondary endpoint of LBW. The 2-sided significance level of 0.00125 (derived as 2 x 0.000625) for the primary endpoint equals the significance level of two independent confirmative trials each conducted at 0.05 2-sided alpha level. Note that to derive this: 0.025 is the 1-sided probability of a false positive for concluding superiority of AZCQ over SP in a single trial, with 0.025 x 0.025 = 0.000625 representing the same overall probability using two independent trials.

The underlying true incidence of the composite primary endpoint in the control group was unknown at the design stage, but required for sample size considerations. So, the design planned one adaptive sample size refinement based on the observed pooled incidence of sub-optimal pregnancy outcomes, without regard to treatment regime, after collection of 1,000 observations for the primary endpoint. Based on the result from this adaptive sample size assessment, the sample size was refined to be 5,044 study participants (maximum allowed for per the protocol; S2 Text) if the study went to completion.

#### Interim efficacy analysis

The final protocol (S2 Text) included one interim analysis at 35% (i.e., following the completion of pregnancy outcome in the first 1,766 of 5,044 study participants to be randomized). Early termination for superiority was to occur if the interim analysis revealed a statistically significant lower risk of sub-optimal pregnancy outcome (primary endpoint) and of LBW (key secondary endpoint) in the AZCQ group compared with SP. The assessment of trial futility at the interim analysis (i.e., low likelihood that AZCQ will demonstrate benefit compared with SP in protective efficacy for IPTp should the study go to completion) was to be based solely on the primary endpoint of sub-optimal pregnancy outcome. Statistical stopping boundaries were employed based on controlling the overall study alpha at 0.00125 and 0.05 for the primary endpoint and LBW, respectively, to account for multiple queries of the data for these two endpoints. The EDMC reviewed interim analysis results and oversaw evaluation of emerging safety data on a regular basis. Although the study was open-label, study personnel involved in the day-to-day operation of the trial remained blinded to interim and aggregate treatment-group results until study termination.

#### Efficacy analyses

The primary analysis population was the intent-to-treat (ITT) population, including all study participants who were randomized, received at least one dose of study medication, and had a singleton gestation. Except for the primary endpoint, as defined above, there were no imputations for missing data.

For dichotomous endpoints (including the primary endpoint), the percentage of study participants meeting the endpoint was estimated for each treatment arm and confidence intervals (CIs) were calculated using the normal approximation to the binomial. The risk ratio (RR) of the proportion of study participants meeting the endpoint (AZCQ/SP) was calculated to compare treatment groups. Mantel-Haenszel estimates of the common RR [27,28] stratified by randomization strata (gravidae) were computed utilizing the estimated variance given by Greenland and Robins [29], and two-sided *p*-values were calculated.

Secondary endpoints involving counts, actual neonate birth weight, and hemoglobin values were analyzed using analysis models of variance (ANOVA) or of covariance (ANCOVA) if a baseline value was available. Model terms included treatment group, randomization strata, and, where applicable, the baseline value. Model adjusted means (least square [LS] means) and corresponding 95% CIs were computed for each treatment group and the difference between treatment groups (AZCQ minus SP).

As noted above, stopping boundaries (i.e., alpha spending functions [30]) were employed to account for the interim analysis involving the primary endpoint and LBW. For the primary endpoint, the adjusted alpha level at the time of final study completion was 0.000109 for determining statistical significance. In the case of LBW, the adjusted alpha level at the final analysis was 0.003031. However, since the study was terminated early for futility based on the primary endpoint, all CIs for all endpoints were stated at the 95% confidence level for informational purposes only and all inferences for all secondary endpoints apart from LBW are to be considered exploratory.

#### Safety analysis

The safety populations included (i) all enrolled study participants who received at least one dose of study medication and (ii) all live-born babies. Descriptive statistics were used to summarize the data.

### Role of the Funding Source

Pfizer and the Medicines for Malaria Venture (MMV) funded this trial and were involved in study design, data analysis, data interpretation, and writing of the study report. All authors had full access to all the data in the study and had final responsibility for the decision to submit for publication. Medical writing and editorial support were provided by Susanne Vidot, PhD, of Engage Scientific and was funded by Pfizer.

## Results

### Interim Analysis Results

The planned interim analysis was performed when the first 1,766 ITT study participants (35%) out of 5,044 planned study participants had an observation for the primary endpoint and these results were reviewed by the EDMC on 12 August 2013. The preliminary estimate of efficacy crossed the futility boundary (estimated relative risk of sub-optimal pregnancy outcome (AZCQ/SP), 1.29; 99.99% CI: 0.80, 2.09). Consequently, the sponsors made the decision to stop the IPTp-AZCQ malaria clinical development program and terminated this study on 19 August 2013. The EDMC, investigators, Ethics Committees, and Regulatory Authorities were notified of this decision in writing on 27 August 2013. Recruitment was stopped, and all active study participants were informed of the termination, switched to the standard of care IPTp treatment per local guidelines, asked to attend discontinuation visits, and followed by the investigators through delivery. All deliveries that occurred prior to study completion, or a study participant’s discontinuation visit, were included in the final ITT analysis. All deliveries that occurred after the study completion, or after a study participant’s discontinuation visit were collected in the Sponsor’s safety database, but excluded from the final ITT analysis.

Due to the early study termination and sensitivity of the primary endpoint to missing pregnancy outcomes (missing outcomes imputed as failure to respond to treatment), an Intent-to-Treat Efficacy Analyzable (ITT EA) analysis population was defined as all ITT study participants whose pregnancy outcome occurred on or before the date that investigators were notified of the interim analysis outcome or who withdrew from the study prior to that point, to allow for a snapshot of the results at that time.

### Study Population and Patient Disposition

**Fig 1:**
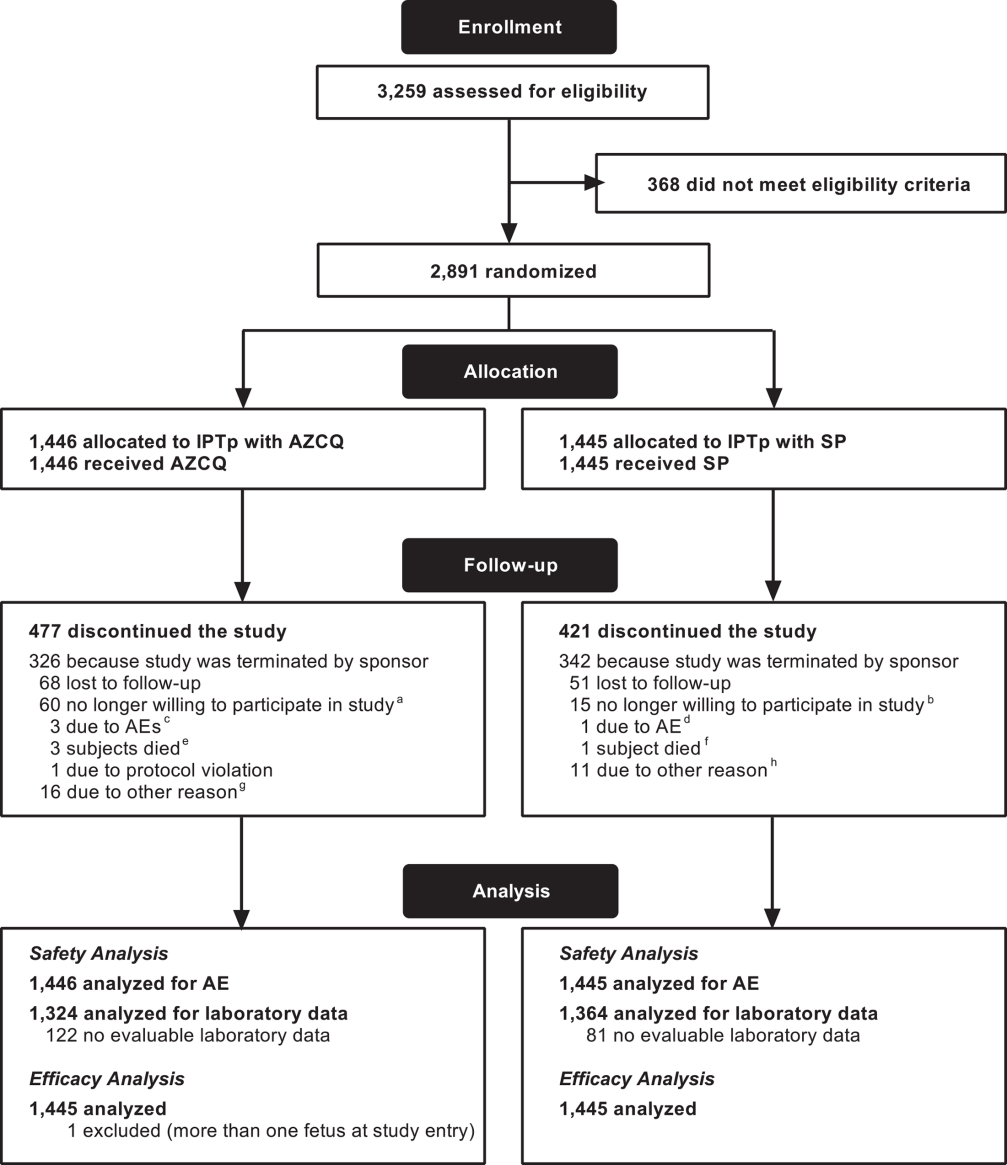
Participant flow chart. ^a^The reasons why study participants were no longer willing to participate included: no specific reason provided, *n* = 17; family, social, or personal issues, *n* = 17; experience of AEs, *n* = 16; no longer willing to take study drug, *n* = 6; relocation, *n* = 3; stillbirth, *n* = 1. ^b^The reasons why study participants were no longer willing to participate included: family, social, or personal issues, *n* = 10; relocation, *n* = 3; no specific reason provided, *n* = 2. ^c^The AEs leading to discontinuation were combinations of nausea, vomiting, asthenia, spontaneous abortion, imminent abortion, and restlessness. ^d^The AE leading to discontinuation was premature rupture of membranes/stillbirth/umbilical cord abnormality. ^e^The causes of deaths were meningitis; postpartum hemorrhage and uterine rupture; and eclampsia. ^f^The death was due to peritonitis and intestinal perforation. ^g^The ‘other’ reasons for discontinuation were: relocation, *n* = 9; family, social, or personal issues, *n* = 5; non-compliance with study procedures, *n* = 2. ^h^The ‘other’ reasons for discontinuation were: relocation, *n* = 5; family, social, or personal issues, *n* = 5; no specific reason provided, *n* = 1.

A total of 3,259 study participants were screened: 2,891 of these were enrolled and randomized to receive AZCQ or SP; 969 of 1,446 (67.0%) study participants in the AZCQ group and 1,024 of 1,445 (70.9%) study participants in the SP group completed the study (Fig 1). The country by country breakdown is as follows: in Kenya, 514 participants were assigned to receive AZCQ and 515 to SP; in Benin, 31 participants were assigned to receive AZCQ and 31 to SP; in Malawi, 305 participants were assigned to receive AZCQ group and 306 to SP; in Tanzania, 420 participants were assigned to receive AZCQ and 419 to SP; in Uganda, 176 participants were assigned to receive AZCQ and 174 to SP.

All study participants were female and 99.9% were black. Baseline characteristics and obstetric history were balanced between the two treatment regimens (Table 1). A total of 866 (59.9%) study participants in the AZCQ group and 864 (59.8%) study participants in the SP group were primi- or secundigravidae.

**Table 1:** Baseline characteristics in the safety population.

		AZCQ	SP
		*N* = 1,446	*N* = 1,445
**Age, yrs**	Mean (SD)	23.3 (4.5)	23.3 (4.6)
	Range	16–35	16–35
**Race, *n* (%)**	Black	1,446 (100.0)	1,444 (99.9)
	Asian	0	1 (0.1)
**Weight, kg**	Mean (SD)	59.8 (9.3)	59.9 (9.5)
	Range	39.3–110.1	35.5–108.5
**Body mass index, kg/m**^**2**^	Mean (SD)	23.8 (3.4)	23.8 (3.5)
	Range	15.8–43.0	16.1–40.1
**Height, cm**	Mean (SD)	158.7 (6.7)	158.7 (6.4)
	Range	135.0–193.0	135.0–192.0
**Number of prior pregnancies**	0	484 (33.5)	481 (33.3)
	1	382 (26.4)	383 (26.5)
	2	280 (19.4)	298 (20.6)
	3	185 (12.8)	168 (11.6)
	4+	114 (7.9)	115 (8.0)
**Prior obstetrical complications, *n* (%)**		51 (3.5)	48 (3.3)
**Risk factors for adverse pregnancy outcomes due to environmental or occupational exposures, *n* (%**)		2 (0.1)	0 (0.0)

In the AZCQ treatment group, 1,104 (76.3%) study participants were dosed on all 9 IPTp treatment days, and in the SP treatment group, 1,245 (86.2%) study participants were dosed on all 3 treatment days. Almost all study participants (AZCQ: 1,420 [98.5%]; SP: 1,418 [98.3%]) installed a bednet over their sleeping area, as required per protocol.

### Final Analysis Results

IPTp-AZCQ was not superior to IPTp-SP in decreasing the relative risk of sub-optimal pregnancy outcome (Tables 2 and 3). A total of 378 (26.2%) study participants in the AZCQ group versus 342 (23.7%) in the SP group had sub-optimal pregnancy outcomes in the ITT population. The computed relative risk (RR of 1.11) was not statistically significant (95% CI: 0.97, 1.25; *p =* 0.12). When excluding missing or unknown values, AZCQ and SP showed near equality (RR of 0.96 in the ITT EA population).

**Table 2:** Incidence of sub-optimal pregnancy outcome (primary endpoint).

	AZCQ	SP	Relative risk estimate (AZCQ/SP)
	*n* (%)	*n* (%)	RRMH^a^; [95% CI]; *p value*
**ITT population**			
**Total outcomes**^b^	**N = 1,445**	**N = 1,445**	
**Sub-optimal pregnancy outcome**	**378 (26.16)**	**342 (23.67)**	**1.11; [0.97, 1.25]; *p* = 0.12237**
Full-term live birth and LBW	24 (1.66)	40 (2.77)	0.60
Full-term live birth and missing birth weight	2 (0.14)	2 (0.14)	1.00
Premature birth and normal birth weight	14 (0.97)	17 (1.18)	0.82
Premature birth and LBW	33 (2.28)	28 (1.94)	1.18
Premature birth and missing birth weight	0 (0.00)	0 (0.00)	−
Spontaneous abortion	6 (0.42)	4 (0.28)	1.50
Induced/elective abortion	1 (0.07)	0 (0.00)	−
Stillbirth	17 (1.18)	17 (1.18)	1.00
Unknown or missing pregnancy outcome^c^	281 (19.45)	234 (16.19)	1.20
**ITT EA analysis population**			
**Total Outcomes**^b^	**N = 1,237**	**N = 1,231**	
**Sub-optimal pregnancy outcome**	**200 (16.2)**	**154 (12.5)**	**1.29; [1.06, 1.57]; *p* = 0.01017**
**ITT EA analysis population excluding study participants with unknown or missing pregnancy outcomes**^d^			
**Total Outcomes**^b^	**N = 1,131**	**N = 1,179**	
**Sub-optimal pregnancy outcome**	**94 (8.31)**	**102 (8.65)**	**0.96; [0.73, 1.25]; *p* = 0.76512**

EA: Efficacy Analyzable.

^a^Mantel-Haenszel estimate of the common relative risk, adjusting for randomization strata (gravidae). A relative risk <1 favors the AZCQ treatment group (reduction in risk for the endpoint).

^b^Number of study participants in the analysis population.

^c^A total of 171/281 study participants (60.9%) in the AZCQ group and 173/234 (73.9%) in the SP group had missing pregnancy outcomes resulting from early termination of the study.

^d^Excludes 106 study participants with unknown and missing pregnancy outcomes in the AZCQ group and 52 in the SP group; most commonly observations were missing because study participants were no longer willing to participate in the study (AZCQ: *n* = 55, SP: *n* = 13) or lost to follow up (AZCQ: *n* = 26, SP: *n* = 24).

**Table 3:** Incidence of sub-optimal pregnancy outcome (primary endpoint) in ITT (efficacy analyzable) population, by country.

	AZCQ		SP	Relative risk estimate (AZCQ/SP)	
	*n* (%)		*n* (%)		
**Benin**					
**Total outcomes**^a^	**N = 29**		**N = 30**		
Sub-optimal pregnancy outcome	3 (10.34)		9 (30.00)	0.34	
Full-term live birth and LBW	2 (6.90)		2 (6.67)	1.03	
Full-term live birth and missing birth weight	0		0	-	
Premature birth and normal birth weight	0		1 (3.33)	0.00	
Premature birth and LBW	1 (3.45)		4 (13.33)	0.26	
Premature birth and missing birth weight	0		0	−	
Spontaneous abortion	0		1 (3.33)	0.00	
Induced/elective abortion	0		0	-	
Stillbirth	0		1 (3.33)	0.00	
Unknown or missing pregnancy outcome	0		0	-	
**Kenya**					
**Total outcomes**^a^	**N = 442**		**N = 438**		
Sub-optimal pregnancy outcome	44 (9.95)		31 (7.08)	1.41	
Full-term live birth and LBW	2 (0.45)		6 (1.37)	0.33	
Full-term live birth and missing birth weight	2 (0.45)		1 (0.23)	1.98	
Premature birth and normal birth weight		10 (2.26)	7 (1.60)		1.42
Premature birth and LBW	12 (2.71)		5 (1.14)	2.38	
Premature birth and missing birth weight	0		0	-	
Spontaneous abortion	2 (0.45)		0	-	
Induced/elective abortion	1 (0.23)		0	−	
Stillbirth	7 (1.58)		3 (0.68)	2.31	
Unknown or missing pregnancy outcome	8 (1.81)		9 (2.05)	0.88	
**Malawi**					
**Total outcomes**^a^	**N = 227**		**N = 228**		
Sub-optimal pregnancy outcome	33 (14.54)		25 (10.96)	1.33	
Full-term live birth and LBW	4 (1.76)		3 (1.32)	1.34	
Full-term live birth and missing birth weight	0		0	-	
Premature birth and normal birth weight	1 (0.44)		3 (1.32)	0.33	
Premature birth and LBW	5 (2.20)		3 (1.32)	1.67	
Premature birth and missing birth weight	0		0	-	
Spontaneous abortion	0		2 (0.88)	0.00	
Induced/elective abortion	0		0	-	
Stillbirth	2 (0.88)		4 (1.75)	0.50	
Unknown or missing pregnancy outcome	21 (9.25)		10 (4.39)	2.11	
**Tanzania**					
**Total outcomes**^a^	**N = 363**		**N = 361**		
Sub-optimal pregnancy outcome	72 (19.83)		63 (17.45)	1.14	
Full-term live birth and LBW	7 (1.93)		12 (3.32)	0.58	
Full-term live birth and missing birth weight	0		0	-	
Premature birth and normal birth weight	3 (0.83)		6 (1.66)	0.50	
Premature birth and LBW	6 (1.65)		9 (2.49)	0.66	
Premature birth and missing birth weight	0		0	-	
Spontaneous abortion	4 (1.10)		0	−	
Induced/elective abortion	0		0	-	
Stillbirth	5 (1.38)		8 (2.22)	0.62	
Unknown or missing pregnancy outcome	47 (12.95)		28 (7.76)	1.67	
**Uganda**					
**Total outcomes**^a^	**N = 176**		**N = 174**		
Sub-optimal pregnancy outcome	48 (27.27)		26 (14.94)	1.83	
Full-term live birth and LBW	9 (5.11)		15 (8.62)	0.59	
Full-term live birth and missing birth weight	0		1 (0.57)	0.00	
Premature birth and normal birth weight	0		0	-	
Premature birth and LBW	8 (4.55)		5 (2.87)	1.58	
Premature birth and missing birth weight	0		0	−	
Spontaneous abortion	0		1 (0.57)	0.00	
Induced/elective abortion	0		0	−	
Stillbirth	3 (1.70)		1 (0.57)	2.97	
Unknown or missing pregnancy outcome	28 (15.91)		3 (1.72)	9.23	

^a^Number of study participants in the analysis population

**Fig 2:**
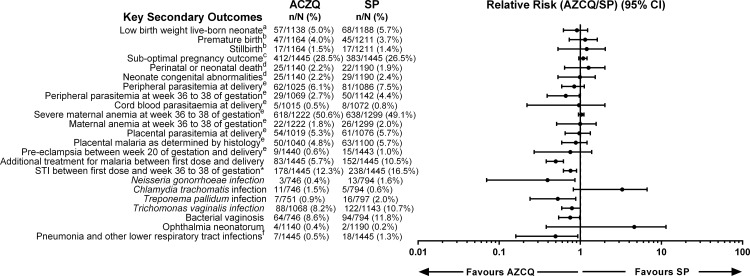
Incidence of secondary outcomes in the ITT population. ^a^Denominators are the number of subjects with a premature or full-term live birth and a non-missing birth weight. ^b^Denominators are the number of pregnancy outcomes, excluding those that were unknown/missing. ^c^Including neonatal death and congenital malformations. ^d^Denominators are the number of subjects with a premature or full-term live birth. ^e^Denominators are the number of subjects with available measurements. ^f^STI = sexually transmitted infection between first dose and Week 36 to 38 of gestation including *T*. *pallidum*, *N*. *gonorrhoeae*, *C*. *trachomatis* (diagnosed based on clinical presentation prior to week 36–38 and/or positive lab test results between week 36–38). ^g^Diagnosed based on positive result at week 36 to 38 of gestation. ^h^Between first dose and delivery

The proportion of study participants with a LBW live-born neonate in the ITT population was marginally lower in the AZCQ group than in the SP group (57 [5.0%] versus 68 [5.7%]); but the estimated relative risk reduction did not reach statistical significance (Fig 2). A number of other secondary outcomes also showed a lower estimated risk in the AZCQ group than in the SP group, with a statistically significant reduction in the number and incidence of STIs, number and incidence of symptomatic malaria episodes, incidence of peripheral parasitemia at weeks 36 to 38 of gestation, and incidence of lower respiratory tract infections (Fig 2). The mean change from baseline to weeks 36–38 of gestation in hemoglobin was significantly greater in the SP group compared with the AZCQ group (0.3 g/dL versus 0.2 g/dL; LS mean estimate of treatment group difference: −0.14, 95% CI: −0.24, −0.03; *p* = 0.01). A full summary of secondary endpoint data is included in S2–S5 Tables.

At day 28 post delivery, in the ITT population, 8/551 participants in the AZCQ group and 17/569 participants in the SP group had nasopharyngeal swabs isolating *S*. *pneumoniae* (S6 Table). No participants in the AZCQ treatment group and 2 (11.8%) participants in the SP group had nasopharyngeal swabs positive for macrolide-resistant *S*. *pneumoniae*. No participants in either the AZCQ or SP treatment group had swabs positive for penicillin-resistant *S*. *pneumoniae*. At 6 months after the last IPTp dose (Visit 7), 16/478 and 11/489 participants in the AZCQ and SP treatment groups respectively, had nasopharyngeal swabs isolating *S*. *pneumoniae*. No participants in either the AZCQ or SP treatment group had swabs positive for macrolide-resistant or penicillin-resistant *S*. *pneumoniae*.

### Safety Results

#### Deaths, stillbirths, and other SAEs

A total of 3 (0.2%) mothers and 25 (2.2%) neonates in the AZCQ group and 1 (0.1%) mother and 22 (1.8%) neonates in the SP group died during the study period (Table 4). In addition, there were 17 (1.5%) stillbirth pregnancy outcomes in the AZCQ group and 17 (1.4%) in the SP group. No deaths were considered related to study drug.

**Table 4:** Overview of adverse events in the safety population.

n (%)	Mothers^a^		Neonates^b^	
	AZCQ	SP	AZCQ	SP
	N = 1,446	N = 1,445	N = 1,149	N = 1,196
Number of AEs	4,068	2,117	677	645
Study participants with AEs	1,185 (82.0)	897 (62.1)	364 (31.7)	389 (32.5)
Study participants with SAEs^c^	65 (4.5)	42 (2.9)	101 (8.8)	104 (8.7)
Study participants with severe AEs	54 (3.7)	23 (1.6)	54 (4.7)	64 (5.4)
Study participants with treatment-related AEs	996 (68.9)	286 (19.8)	4 (0.3)	2 (0.2)
Number of deaths	3 (0.2)	1 (0.1)	25 (2.2)	22 (1.8)
Study participants who discontinued treatment due to AEs	41 (2.8)	5 (0.3)	—	—
Study participants who temporarily discontinued treatment due to AEs	11 (0.8)	1 (0.1)	—	—

^a^Includes all event incidences that occurred between the first dose of study drug and up to 35 days after the last dose of study drug

^b^Includes all events recorded

^c^These events that met ICH GCP criteria to be considered as SAEs may differ from the total number of LBW baby and premature baby that were reported as study endpoints

All-causality SAEs within 35 days of last dose occurred in 65 (4.5%) mothers in the AZCQ group and in 42 (2.9%) mothers in the SP group, as well as in 101 (8.8%) neonates in the AZCQ group and 104 (8.7%) neonates in the SP group (Table 5). The most common SAEs are listed in Table 5. The only SAEs that were considered treatment related occurred in 5 (0.3%) mothers in the AZCQ group and included vomiting, dizziness, diarrhea, and asthenia. Pruritis and generalized pruritis occurred with low frequency (<5%) in both AZCQ and SP treatment groups.

**Table 5:** Most common serious adverse events in the safety population.

System Organ Class, *n* (%)	AZCQ	SP
Preferred Term, *n* (%)		
**Most common SAEs in mothers (≥5 study participants in either treatment group)**^a^		
	**N = 1,446**	**N = 1,445**
Infections and infestations	8 (0.6)	13 (0.9)
Malaria	2 (0.1)	9 (0.6)
Pregnancy, puerperium, and perinatal conditions^b^	49 (3.4)	27 (1.9)
Hemorrhage in pregnancy	7 (0.5)	1 (0.1)
Pre-eclampsia	3 (0.2)	5 (0.3)
Premature delivery	7 (0.5)	5 (0.3)
Stillbirth	5 (0.3)	7 (0.5)
**Most common SAEs in neonates (≥5 neonates in either treatment group)**^c^		
	**N = 1,149**	**N = 1,196**
Congenital, familial, and genetic disorders	28 (2.4)	30 (2.5)
Polydactyly	16 (1.4)	21 (1.8)
Infections and infestations	32 (2.8)	35 (2.9)
Neonatal infection	5 (0.4)	4 (0.3)
Pneumonia	9 (0.8)	10 (0.8)
Sepsis neonatal	11 (1.0)	13 (1.1)
Pregnancy, puerperium, and perinatal conditions^b^	24 (2.1)	19 (1.6)
LBW baby	9 (0.8)	10 (0.8)
Premature baby	17 (1.5)	12 (1.0)
Respiratory, thoracic, and mediastinal disorders	15 (1.3)	23 (1.9)
Neonatal asphyxia	6 (0.5)	9 (0.8)
Neonatal aspiration	1 (0.1)	5 (0.4)

^a^Includes all event incidences that occurred between the first dose of study drug and up to 35 days after the last dose of study drug

^b^These events that met ICH GCP criteria to be considered as SAEs may differ from the total number of LBW baby and premature baby that were reported as study endpoints

^c^Includes all events recorded

#### All-causality and treatment-related AEs

All-causality AEs were reported in 1,185 (82.0%) versus 897 (62.1%) mothers, and in 364 (31.7%) versus 389 (32.5%) neonates, in the AZCQ and SP treatment groups, respectively. The most common all-causality AEs are listed in Table 6. Most were mild or moderate in severity. Treatment-related AEs occurred in a larger proportion of mothers in the AZCQ than in the SP group (996 [68.9%] versus 286 [19.8%]), the most frequent events being vomiting (experienced by 645 [44.6%] in AZCQ and 73 [5.1%] in SP group), dizziness (experienced by 454 [31.4%] in AZCQ and 62 [4.3%] in SP group), headache (experienced by 221 [15.3%] in AZCQ and 131 [9.1%] in SP group), and asthenia (experienced by 220 [15.2%] in AZCQ and 21 [1.5%] in SP group). Treatment-related AEs were reported for 4 (0.3%) neonates in the AZCQ group and 2 (0.2%) neonates in the SP group and they included LBW, neonatal anemia, neonatal jaundice, and prematurity.

**Table 6:** The most common all-causality adverse events in the safety population.

System Organ Class, *n* (%)	AZCQ	SP
Preferred Term, *n* (%)^a^		
**Most common AEs in mothers (≥5% of study participants in either treatment group)**^b^		
	**N = 1,446**	**N = 1,445**
Blood and lymphatic system disorders	207 (14.3)	192 (13.3)
Anemia	206 (14.2)	192 (13.3)
Eye disorders	146 (10.1)	2 (0.1)
Vision blurred	145 (10.0)	1 (0.1)
Gastrointestinal disorders	856 (59.2)	269 (18.6)
Abdominal discomfort	123 (8.5)	50 (3.5)
Abdominal pain	120 (8.3)	36 (2.5)
Diarrhea	206 (14.2)	14 (1.0)
Nausea	216 (14.9)	58 (4.0)
Vomiting	653 (45.2)	96 (6.6)
General disorders and administration site conditions	344 (23.8)	120 (8.3)
Asthenia	240 (16.6)	40 (2.8)
Fatigue	81 (5.6)	22 (1.5)
Infections and infestations	435 (30.1)	498 (34.5)
Malaria	51 (3.5)	130 (9.0)
Upper respiratory tract infection	127 (8.8)	153 (10.6)
Urinary tract infection	105 (7.3)	117 (8.1)
Vulvovaginal candidiasis	75 (5.2)	60 (4.2)
Investigations	160 (11.1)	169 (11.7)
White blood cells urine positive	149 (10.3)	162 (11.2)
Nervous system disorders	660 (45.6)	271 (18.8)
Dizziness	463 (32.0)	84 (5.8)
Headache	300 (20.7)	219 (15.2)
Pregnancy, puerperium, and perinatal conditions	78 (5.4)	75 (5.2)
**Most common AEs in neonates (≥5% neonates in either treatment group)**^c^		
	**N = 1,149**	**N = 1,196**
Infections and infestations	248 (21.6)	256 (21.4)
Upper respiratory tract infection	126 (11.0)	116 (9.7)
Pregnancy, puerperium, and perinatal conditions	72 (6.3)	81 (6.8)

^a^MedDRA preferred term

^b^Includes all events that occurred between the first dose of study drug and up to 35 days after the last dose of study drug

^c^Includes all events recorded

Forty-one (2.8%) mothers in the AZCQ group compared with 5 (0.3%) mothers in the SP group discontinued treatment due to AEs, the most common being vomiting (25 [1.7%] mothers in the AZCQ and 1 [0.1%] in the SP group) and dizziness (9 [0.6%] mothers in the AZCQ and none in the SP group).

#### Neonate outcomes

There were 1,067 (93.4%) and 1,102 (92.6%) normal newborns born to mothers in the AZCQ and SP treatment groups, respectively. The proportions with congenital anomalies were comparable among neonates born to mothers in the AZCQ and SP groups (25 [2.2%] versus 29 [2.4%]).

## Discussion

This randomized controlled study was the first evaluation of AZCQ combination therapy for use in IPTp. The study was stopped early when a predefined interim analysis after accrual of 35% of the planned recruitment level showed that it is unlikely that IPTp-AZCQ would demonstrate a reduction in the incidence of sub-optimal pregnancy outcomes relative to the current standard of care, IPTp-SP. In the ITT population, there was no statistically significant difference in the proportion of study participants with a sub-optimal pregnancy outcome in the AZCQ and SP treatment groups. Yet, the incidence of symptomatic malaria and peripheral parasitemia at weeks 36 to 38 as well as of STIs and lower respiratory tract infections was significantly lower in the AZCQ than in the SP group.

In this population of 2,891 pregnant women from five sub-Saharan African countries, the primary efficacy endpoint of a sub-optimal pregnancy outcome was observed in approximately 1 in 4 study participants in both treatment groups. The estimated relative risk of a sub-optimal pregnancy outcome in the AZCQ compared with the SP group was marginally higher; however, this result was not statistically significant. Furthermore, the two treatment regimens showed near equality when excluding missing or unknown values, which occurred more frequently in the AZCQ treatment group in part due to reduced tolerability (IPTp-AZCQ was less well-tolerated than IPTp-SP). The estimated relative risk of LBW–a key secondary endpoint—was lower in the AZCQ treatment group compared with SP, but not statistically significant. A recent meta-analysis demonstrated that a three-dose IPTp-SP regime in pregnant women who are negative for HIV is associated with a median LBW of 82 per 1,000 (95% CI 67 to 100) [12]. In comparison, the observed incidence of LBW of 5.0% in the AZCQ and 5.7% in the SP group was low, despite the study being conducted in sites with reported SP resistance. This may be due, in part, to care received at regular ANC visits and concurrent LLIN use.

In spite of not meeting the primary end-point, AZCQ was associated with significant improvements relative to SP in a number of secondary endpoints thought to be relevant to IPTp, including the number and incidence of symptomatic malaria episodes, and the incidence of peripheral parasitemia at weeks 36 to 38 of gestation. In addition, the number and incidence of STIs and the incidence of lower respiratory tract infections were also significantly lower in the AZCQ treatment regime compared with SP. However, these improvements did not translate into a significant benefit on the primary and LBW endpoints. Similarly, a recent open-label randomized trial of IPTp with mefloquine versus IPTp-SP found no difference in the incidence of LBW between mefloquine and SP recipients, despite a lower incidence of maternal parasitemia in the mefloquine group [31].

Combination therapy with AZCQ for IPTp in pregnant women from the second trimester was less well-tolerated than SP in this study. Study participants receiving AZCQ were less likely to complete all treatment days than study participants receiving SP. AEs such as vomiting, dizziness, headache, and asthenia were reported more frequently by study participants receiving AZCQ than those receiving SP. Likewise, SAEs and discontinuations due to AEs were more frequent in mothers in the AZCQ treatment group than in the SP treatment group. However, the rates of congenital malformation/anomalies, neonatal deaths, and SAEs were comparable for neonates born to mothers in the two treatment regimens.

### Impact of Regular ANC Clinic Visits and LLIN Use

It has been previously observed that effective malaria control strategies, such as high rates of ANC compliance and LLIN use, result in a lower incidence of sub-optimal pregnancy outcomes [31]. Indeed, clinical trials and field programs have established that LLINs substantially reduce the risk of adverse consequences of malaria in pregnancy, including maternal anemia, severe anemia, peripheral and placental malaria, and low birth weight [32–34]. Thus, concurrent use of LLINs, regular ANC attendance, prompt diagnosis of acute malaria, and effective treatment in this clinical trial could have reduced the influence of malaria on birth outcomes in both treatment groups. These factors, in addition to potentially decreased malaria transmission in the study areas, would reduce the statistical power to detect differences between treatment interventions and may explain the comparable preventive benefit of IPTp-AZCQ and IPTp-SP on sub-optimal pregnancy outcomes in this study. This explanation is supported by the low incidence of LBW observed across both treatment groups in this study.

### Limitations of Study, Analysis, or Data

Due to the early termination of this study, confidence bounds are wider than if the trial had gone to completion; therefore, estimated treatment differences may be biased. However, given the clear lack of evidence in support of the alternative hypothesis after observation of a pregnancy outcome in 2,891 study participants, it is unlikely that enrolling more study participants would have changed the result. Similarly, the conditional power for LBW was low and it is unlikely that the treatment group difference apparent in the final analysis after early termination would have reached statistical significance if the study had completed. As the study was negative for the primary endpoint (sub-optimal pregnancy outcome) and LBW, all inferences for the other secondary endpoints should be considered exploratory and 95% CI and *p* values should not be over-interpreted.

Because primi- and secundigravidae (G1-G2) women are at greatest risk of LBW secondary to malaria infection, they may also have a more marked response to anti-malarial treatment compared to other multigravidae women (G3+). Therefore, future IPTp studies could elucidate the impact of gravidity on outcomes by conducting efficacy analyses separately for G1/G2 and G3+ women.

When considering the findings of this study, it is also important to bear in mind that its superiority design was based on the expectation that protective effectiveness of IPTp-SP on pregnancy outcomes was significantly reduced at the study sites by *P*. *falciparum* resistance to SP. This assumption is supported by reports of reduction in IPTp-SP effectiveness in areas of high prevalence of SP resistance (*pfdhps*A581G and *pfdhps*K540E mutations), while IPTp-SP continues to be effective in areas of low and moderate SP resistance [35].

### Clinical Implications

This study failed in its primary objective to demonstrate a greater benefit of IPTp-AZCQ than IPTp-SP on pregnancy outcomes, and failed to demonstrate that, at this time, three 3-day courses of 1,000/620 mg AZCQ QD is a suitable replacement for three 1-day courses of IPTp-SP. Therefore, taken together with recent findings that IPTp with mefloquine did not outperform and had lower tolerability than IPTp-SP [31,36], the current study suggests that SP remains the best available artemisinin-sparing option for IPTp. According to the World Malaria Report 2014, although the proportion of pregnant women receiving IPTp-SP has been increasing over the last decade and 57% of pregnant women in countries that adopted IPTp received at least one dose of IPTp in 2013, the rates of IPTp administration remain below Global Malaria Action Plan (GMAP) targets [9]. Importantly, attendance rates of pregnant women at ANC clinics markedly exceed the rates of IPTp administration (89% versus 57%), suggesting that existing opportunities to deliver IPTp at antenatal clinics are being missed [9].

The findings of this study also stress the importance of malaria transmission control with LLINs. As with IPTp-SP, the use of insecticide-treated mosquito nets has increased but remains far below the GMAP target to achieve universal access to and utilization by every person at risk, and there is room for further improvements [9].

Although AZCQ did not meet the pre-specified primary endpoint, the observed reductions in symptomatic malaria episodes, peripheral parasitemia, STIs, and lower respiratory tract infections in the AZCQ versus the SP group suggest a protective antimalarial and antibiotic effect of IPTp-AZCQ in pregnant African women of the sub-Saharan region. This suggestion is in line with our findings in an open-label, non-comparative study (NCT01103713) of the parasitological response in primi- and secundigravidae with asymptomatic malaria after a 3-day course of AZCQ (1,000/620 mg QD), reporting a parasitological response at day 28 of 99.35% (95% CI: 97.8, 100.0). Moreover, the same dosing regime of AZCQ was also efficacious in sub-Saharan Africa in the treatment of symptomatic uncomplicated malaria in two multi-country Phase 3 clinical studies in non-pregnant adults [21] and one study in children (NCT00677833). Collectively, these findings suggest that, in view of increasing concerns regarding the emergence of *P*. *falciparum* resistance to artemisinins, AZCQ may still be a valuable alternative in the treatment of uncomplicated malaria, although further research is needed.

### Conclusion

This open-label, Phase 3, randomized clinical trial failed to demonstrate a significantly higher reduction in the risk of sub-optimal pregnancy outcomes among pregnant women in stable *P*. *falciparum* transmission areas in sub-Saharan Africa who receive IPTp with AZCQ rather than SP. Hence, there remains an urgent unmet need to identify alternative well-tolerated and efficacious drugs for IPTp, or to develop alternative strategies to complement the use of LLINs as well as prompt diagnosis and effective treatment of symptomatic malaria in preventing the adverse consequences of malaria in pregnancy. General results from this trial also indicate that AZCQ warrants further investigation as an alternative for the treatment of uncomplicated malaria.

## Supporting Information

S1 Fig Study schematic. ^a^Assessments included recording of medical history, obstetrical history, and concomitant treatment, physical exam, vital signs, urine pregnancy test, routine obstetric check-up, ultrasound examination, tests for hemoglobin, proteinuria, and glycosuria, and screening for syphilis (*Treponema pallidum*). ^b^Each IPTp-AZCQ treatment course comprised an ANC visit with the investigator at the health clinic on day 0, and two home visits by field workers on days 1 and 2 for administration of second and third doses of AZCQ and adverse event reporting. Each IPTp-SP treatment course comprised an ANC visit with the investigator at the health clinic on day 0 and a home visit by a field worker on day 2 for adverse event reporting. ^c^Assessments also included reporting of concomitant treatment, vital signs, and adverse events; as well as a routine obstetric check-up and tests for hemoglobin, proteinuria, and glycosuria. ^d^Every effort was made to encourage women to deliver in hospital. In case of a home delivery, study participants were to notify the study site within 24 hours of occurence and were followed up by field workers within 2 days of reporting the event. ^e^Assessments also included reporting of concomitant treatment, vital signs, and adverse events; as well as physical exam of mother.
(DOCX)

S1 Table Eligibility criteria. (DOCX)

S2 Table Secondary pregnancy outcomes in the ITT population. Statistically significant findings are highlighted in grey. ^a^Denominators are the number of subjects with a premature or full-term live birth and a non-missing birth weight. ^b^Denominators are the number of pregnancy outcomes, excluding those that were unknown/missing. ^c^Denominators are the number of subjects with a premature or full-term live birth.
(DOCX)

S3 Table Pre-eclampsia, maternal anemia, and hemoglobin concentration in the ITT population. Statistically significant findings are highlighted in grey. ^a^Pre-eclampsia was diagnosed based on: (1) systolic blood pressure of ≥140 mmHg or of diastolic blood pressure ≥90 mmHg in 2 separate measurement taken ≥4 hours apart and (2) proteinuria (defined as ≥300 mg protein in 24 hour urine collection). ^b^Denominators are the number of subjects with available measurements.
(DOCX)

S4 Table *P*. *falciparum* parasitemia in the ITT population. Statistically significant findings are highlighted in grey. ^a^Denominators are the number of subjects with available measurements.
(DOCX)

S5 Table Other infections in the ITT population. Statistically significant findings are highlighted in grey. ^a^Denominators are the number of subjects with available measurements. ^b^Denominators are the number of subjects with a premature or full-term live birth.
(DOCX)

S6 Table Nasopharyngeal swaps positive for macrolide- and penicillin-resistant *S*. *pneumoniae*. (DOCX)

S1 Text A0661158 Clinical study protocol. (DOC)

S2 Text CONSORT Checklist. (PDF)

## Abbreviations

AE: adverse event
ANC: antenatal care
AZ: azithromycin
AZCQ: azithromycin-chloroquine
CI: confidence interval
CQ: chloroquine
EDMC: External Data Monitoring Committee
IPTp: intermittent preventive treatment in pregnancy (IPTp)
IPTp-AZCQ: IPTp with AZCQ
IPTp-SP: IPTp with SP
LLIN: long-lasting insecticide-treated bednet(s)
ITT: intention to treat
ITT EA: intention to treat efficacy analyzable
LBW: low birth weight
LS mean: least square mean
SAE: serious adverse event
SD: standard deviation
SP: sulfadoxine-pyrimethamine
RR: risk ratio
