# Supplementary material for: Efficacy and Safety of Azithromycin-Chloroquine versus Sulfadoxine-Pyrimethamine for Intermittent Preventive Treatment of Plasmodium falciparum Malaria Infection in Pregnant Women in Africa: An Open-Label, Randomized Trial
Source: PLoS One. 2016 Jun 21;11(6):e0157045. doi: 10.1371/journal.pone.0157045 (PMC4915657; doi:10.1371/journal.pone.0157045)
Supplement: S1 Fig — aAssessments included recording of medical history, obstetrical history, and concomitant treatment, physical exam, vital signs, urine pregnancy test, routine obstetric check-up, ultrasound examination, tests for hemoglobin, proteinuria, and glycosuria, and screening for syphilis (Treponema pallidum). bEach IPTp-AZCQ treatment course comprised an ANC visit with the investigator at the health clinic on day 0, and two home visits by field workers on days 1 and 2 for administration of second and third doses of AZCQ and adverse event reporting. Each IPTp-SP treatment course comprised an ANC visit with the investigator at the health clinic on day 0 and a home visit by a field worker on day 2 for adverse event reporting. cAssessments also included reporting of concomitant treatment, vital signs, and adverse events; as well as a routine obstetric check-up and tests for hemoglobin, proteinuria, and glycosuria. dEvery effort was made to encourage women to deliver in hospital. In case of a home delivery, study participants were to notify the study site within 24 hours of occurence and were followed up by field workers within 2 days of reporting the event. eAssessments also included reporting of concomitant treatment, vital signs, and adverse events; as well as physical exam of mother. (DOCX) [file pone.0157045.s001.docx]

**S1 Fig. Study schematic.**

**
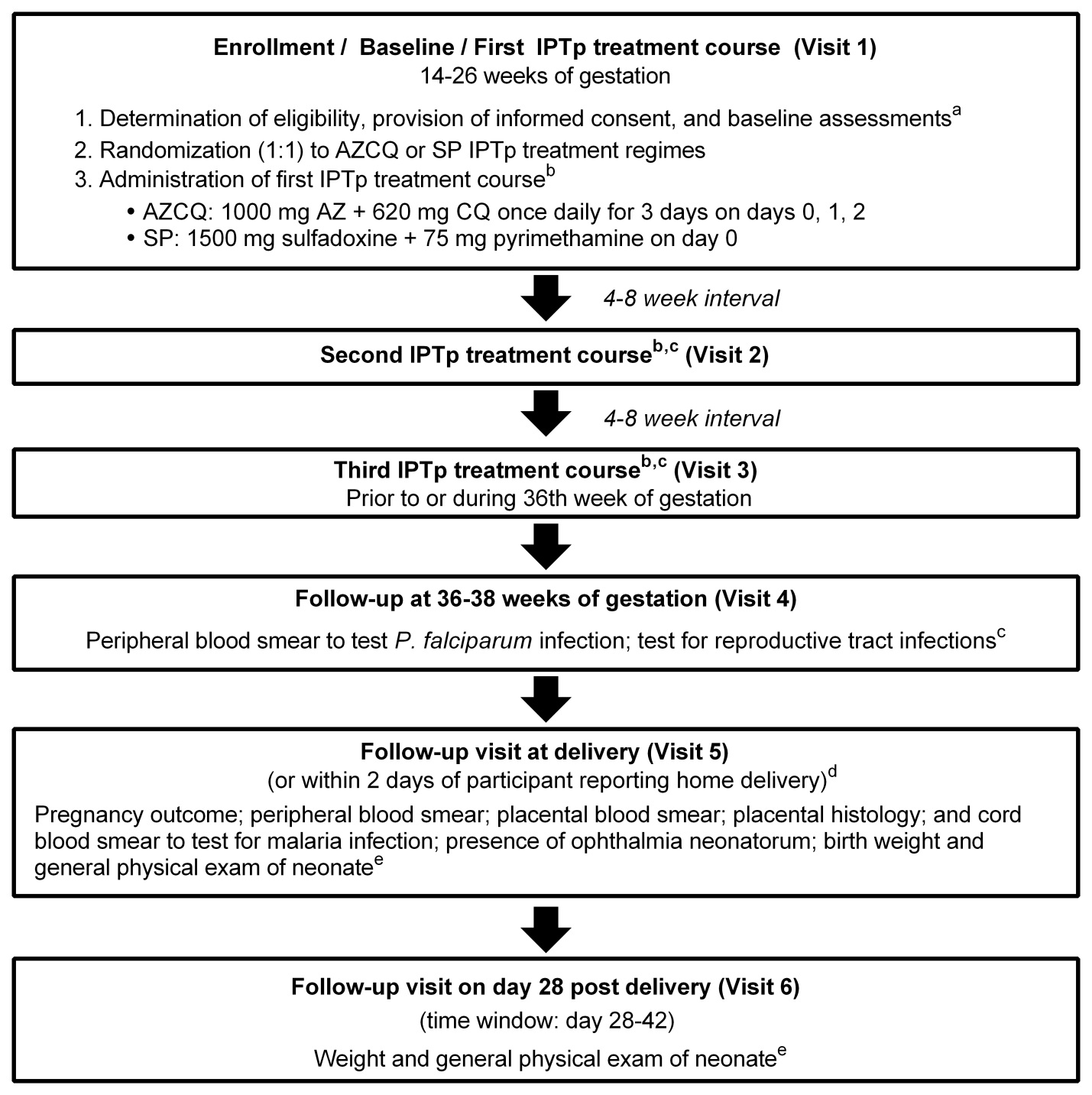
**

^a^Assessments included recording of medical history, obstetrical history, and concomitant treatment, physical exam, vital signs, urine pregnancy test, routine obstetric check-up, ultrasound examination, tests for hemoglobin, proteinuria, and glycosuria, and screening for syphilis (*Treponema pallidum*).

^b^Each IPTp-AZCQ treatment course comprised an ANC visit with the investigator at the health clinic on day 0, and two home visits by field workers on days 1 and 2 for administration of second and third doses of AZCQ and adverse event reporting. Each IPTp-SP treatment course comprised an ANC visit with the investigator at the health clinic on day 0 and a home visit by a field worker on day 2 for adverse event reporting.

^c^Assessments also included reporting of concomitant treatment, vital signs, and adverse events; as well as a routine obstetric check-up and tests for hemoglobin, proteinuria, and glycosuria.

^d^Every effort was made to encourage women to deliver in hospital. In case of a home delivery, study participants were to notify the study site within 24 hours of occurence and were followed up by field workers within 2 days of reporting the event.

^e^Assessments also included reporting of concomitant treatment, vital signs, and adverse events; as well as physical exam of mother.
