# Supplementary material for: Efficacy and Safety of Azithromycin-Chloroquine versus Sulfadoxine-Pyrimethamine for Intermittent Preventive Treatment of Plasmodium falciparum Malaria Infection in Pregnant Women in Africa: An Open-Label, Randomized Trial
Source: PLoS One. 2016 Jun 21;11(6):e0157045. doi: 10.1371/journal.pone.0157045 (PMC4915657; doi:10.1371/journal.pone.0157045)
Supplement: S1 Table — (DOCX) [file pone.0157045.s002.docx]

**S1 Table. Eligibility criteria.**

| **Inclusion Criteria**   1. Pregnant women (all gravidae) with ≥14 and ≤26 weeks of gestational age (as defined by ultrasound examination). 2. Evidence of a personally signed and dated informed consent document indicating that the subject (or a legally acceptable representative if a subject is <18 years of age) has been informed of all pertinent aspects of the study and that all questions by the subject have been sufficiently answered. Assent was obtained from subjects <18 years of age. 3. Subjects who are willing and able to comply with scheduled visits, treatment plan, laboratory tests, and other study procedures. 4. Subjects who agree to be supervised for treatment administration, and are available for all follow-up at delivery and at day 28 post delivery. |
| --- |
| **Exclusion criteria**   1. Age <16 years or >35 years. 2. Multiple gestations (more than one fetus) as per the ultrasound results at screening. 3. Clinical symptoms of malaria. 4. Hemoglobin <8 g/dl (measured at enrollment). 5. Any condition requiring hospitalization at enrollment. 6. Use of antimalarial drugs in previous 4 weeks. 7. History of convulsions, hypertension, diabetes or any other chronic illness that may adversely affect fetal growth and viability. 8. Inability to tolerate oral treatment in tablet form. 9. Known allergy to the study drugs or to any macrolides or sulfonamides. 10. History of smoking or alcohol or drug abuse since first becoming aware of current pregnancy. 11. Participation in another investigational study within 30 days before the current study and/or during study participation. 12. Inability to comprehend and/or unwillingness to follow the study protocol. 13. Concurrent participation in another investigational study. 14. Previous randomization in the current study. 15. Requirement to use medication during the study that might interfere with the evaluation of the study drug (e.g., trimethoprim-sulfamethoxazole use in subjects positive for HIV infection) or that is contraindicated in pregnancy per package inserts. 16. Other severe acute or chronic medical or psychiatric condition or laboratory abnormality that may increase the risk associated with study participation. 17. Evidence of current obstetric complications that may adversely impact the pregnancy and/or fetal outcomes, including presence of congenital anomalies, placenta previa, or abruption. 18. Known severe Sickle Cell (SS) disease or Sickle Hemoglobin C (SC) anemia. 19. Known family history of prolonged QT Syndrome, serious ventricular arrhythmia, or sudden cardiac death. |
