# Supplementary material for: Efficacy and Safety of Azithromycin-Chloroquine versus Sulfadoxine-Pyrimethamine for Intermittent Preventive Treatment of Plasmodium falciparum Malaria Infection in Pregnant Women in Africa: An Open-Label, Randomized Trial
Source: PLoS One. 2016 Jun 21;11(6):e0157045. doi: 10.1371/journal.pone.0157045 (PMC4915657; doi:10.1371/journal.pone.0157045)
Supplement: S2 Table — Statistically significant findings are highlighted in grey. aDenominators are the number of subjects with a premature or full-term live birth and a non-missing birth weight. bDenominators are the number of pregnancy outcomes, excluding those that were unknown/missing. cDenominators are the number of subjects with a premature or full-term live birth. (DOCX) [file pone.0157045.s003.docx]

**S2 Table. Secondary pregnancy outcomes in the ITT population.**

| **Secondary Endpoint** | **AZCQ**  **n/N (%)** | **SP**  **n/N (%)** | **Relative risk estimate (AZCQ/SP)**  **RRMH; [95% CI]; *p* value** |
| --- | --- | --- | --- |
| LBW live-born neonate (<2500 g)^a^ | 57/1138 (5.0) | 68/1188 (5.7) | 0.87; [0.62, 1.23]; *p*=0.4428 |
| Premature birth^b^ | 47/1164 (4.0) | 45/1211 (3.7) | 1.09; [0.73, 1.62]; p=0.6833 |
| Stillbirth^b^ | 17/1164 (1.5%) | 17/1211 (1.4%) | 1.04; [0.53, 2.03]; p=0.9021 |
| Perinatal or neonatal death^c^ | 25/1140 (2.2%) | 22/1190 (1.9%) | 1.14; [0.64, 2.01]; *p*=0.6542 |
| Neonate congenital abnormalities^c^ | 25/1140 (2.2%) | 29/1190 (2.4%) | 0.90; [0.53, 1.53]; *p*=0.6978 |
| Sub-optimal pregnancy outcome, including neonatal deaths and congenital malformations | 412/1445 (28.5) | 383/1445 (26.5) | 1.08; [0.96, 1.21]; *p*=0.2265 |
|  | **AZCQ**  **mean (SD)** | **SP**  **mean (SD)** | **Difference (AZCQ-SP)**  **LS mean; [95% CI]; *p* value** |
| Neonate birth weight, g | 3134.4 (489.5) | 3132.4 (468.8) | 2.1; [−36.5, 40.8]; p=0.9145 |

Statistically significant findings are highlighted in grey.

^a^Denominators are the number of subjects with a premature or full-term live birth and a non-missing birth weight.

^b^Denominators are the number of pregnancy outcomes, excluding those that were unknown/missing.

^c^Denominators are the number of subjects with a premature or full-term live birth.
