# Supplementary material for: Efficacy and Safety of Azithromycin-Chloroquine versus Sulfadoxine-Pyrimethamine for Intermittent Preventive Treatment of Plasmodium falciparum Malaria Infection in Pregnant Women in Africa: An Open-Label, Randomized Trial
Source: PLoS One. 2016 Jun 21;11(6):e0157045. doi: 10.1371/journal.pone.0157045 (PMC4915657; doi:10.1371/journal.pone.0157045)
Supplement: S3 Table — Statistically significant findings are highlighted in grey. aPre-eclampsia was diagnosed based on: (1) systolic blood pressure of ≥140 mmHg or of diastolic blood pressure ≥90 mmHg in 2 separate measurement taken ≥4 hours apart and (2) proteinuria (defined as ≥300 mg protein in 24 hour urine collection). bDenominators are the number of subjects with available measurements. (DOCX) [file pone.0157045.s004.docx]

**S3 Table. Pre-eclampsia, maternal anemia, and hemoglobin concentration in the ITT population.**

| **Secondary Endpoint** | **AZCQ**  **n/N (%)** | **SP**  **n/N (%)** | **Relative risk estimate (AZCQ/SP)**  **RRMH; [95% CI]; *p* value** |
| --- | --- | --- | --- |
| Pre-eclampsia between week 20 of gestation and delivery^a,b^ | 9/1440 (0.6%) | 15/1443 (1.0%) | 0.61; [0.27, 1.38]; p=0.2321 |
| Maternal anemia at week 36 to 38 of gestation (hemoglobin <11g/dl)^b^ | 618/1222 (50.6) | 638/1299 (49.1) | 1.03; [0.95, 1.11]; p=0.4605 |
| Severe maternal anemia at week 36 to 38 of gestation (hemoglobin <8g/dl)^b^ | 22/1222 (1.8) | 26/1299 (2.0) | 0.90; [0.51, 1.57]; p=0.7035 |
|  | **AZCQ**  **mean (SD)** | **SP**  **mean (SD)** | **Difference (AZCQ-SP)**  **LS mean; [95% CI]; *p* value** |
| Change in hemoglobin from baseline to week 36 to 38 of gestation, g/dl | 0.2 (1.6) | 0.3 (1.5) | −0.14; [−0.24, −0.03]; *p* = 0.0131 |

Statistically significant findings are highlighted in grey.

^a^Pre-eclampsia was diagnosed based on: (1) systolic blood pressure of ≥140 mmHg or of diastolic blood pressure ≥90 mmHg in 2 separate measurement taken ≥4 hours apart and (2) proteinuria (defined as ≥300 mg protein in 24 hour urine collection).

^b^Denominators are the number of subjects with available measurements.

Data verification completed by Shuang Li, 21 November 2014.
