# Supplementary material for: Efficacy and Safety of Azithromycin-Chloroquine versus Sulfadoxine-Pyrimethamine for Intermittent Preventive Treatment of Plasmodium falciparum Malaria Infection in Pregnant Women in Africa: An Open-Label, Randomized Trial
Source: PLoS One. 2016 Jun 21;11(6):e0157045. doi: 10.1371/journal.pone.0157045 (PMC4915657; doi:10.1371/journal.pone.0157045)
Supplement: S4 Table — Statistically significant findings are highlighted in grey. aDenominators are the number of subjects with available measurements. (DOCX) [file pone.0157045.s005.docx]

**S4 Table. *P. falciparum* parasitemia in the ITT population.**

| **Secondary Endpoint** | **AZCQ**  **n/N (%)** | **SP**  **n/N (%)** | **Relative risk estimate (AZCQ/SP)**  **RRMH; [95% CI]; *p* value** |
| --- | --- | --- | --- |
| Peripheral parasitemia at week 36 to 38 of gestation^a^ | 29/1069 (2.7%) | 50/1142 (4.4%) | 0.62; [0.39, 0.97]; *p*=0.0360 |
| Peripheral parasitemia at delivery^a^ | 62/1025 (6.1%) | 81/1086 (7.5%) | 0.81; [0.59, 1.12]; *p*=0.1975 |
| Cord blood parasitemia at delivery^a^ | 5/1015 (0.5%) | 8/1072 (0.8%) | 0.66; [0.22, 2.01]; *p*=0.4655 |
| Placental parasitemia at delivery^a^ | 54/1019 (5.3) | 61/1076 (5.7) | 0.93; [0.65, 1.33]; *p*=0.7105 |
| Placental malaria as determined by histology^a^ | 50/1040 (4.8) | 63/1100 (5.7) | 0.84; [0.59, 1.21]; *p*=0.3468 |
| Additional treatment for malaria between first dose and delivery | 83/1445 (5.7%) | 152/1445 (10.5%) | 0.49; [0.38, 0.62]; p<0.0001 |
|  | **AZCQ**  **mean (SD)** | **SP**  **mean (SD)** | **Difference (AZCQ-SP)**  **LS mean; [95% CI]; *p* value** |
| Number of episodes of symptomatic malaria per study participant from first dose to delivery | 0.06 (0.27) | 0.13 (0.42) | −0.07; [−0.09, −0.04]; p<0.0001 |

Statistically significant findings are highlighted in grey.

^a^Denominators are the number of subjects with available measurements.
