# Supplementary material for: Efficacy and Safety of Azithromycin-Chloroquine versus Sulfadoxine-Pyrimethamine for Intermittent Preventive Treatment of Plasmodium falciparum Malaria Infection in Pregnant Women in Africa: An Open-Label, Randomized Trial
Source: PLoS One. 2016 Jun 21;11(6):e0157045. doi: 10.1371/journal.pone.0157045 (PMC4915657; doi:10.1371/journal.pone.0157045)
Supplement: S5 Table — Statistically significant findings are highlighted in grey. aDenominators are the number of subjects with available measurements. bDenominators are the number of subjects with a premature or full-term live birth. (DOCX) [file pone.0157045.s006.docx]

**S5 Table. Other infections in the ITT population.**

| **Secondary Endpoint** | **AZCQ**  **n/N (%)** | **SP**  **n/N (%)** | **Relative risk estimate (AZCQ/SP)**  **RRMH; [95% CI]; *p* value** |
| --- | --- | --- | --- |
| STI between first dose and week 36 to 38 of gestation | 178/1445 (12.3%) | 238/1445 (16.5%) | 0.75; [0.62, 0.90]; p=0.0016 |
| Positive result for STI/RTI at week 36 to 38 of gestation^a^ |  |  |  |
| *Chlamydia trachomatis* | 11/746 (1.5%) | 5/794 (0.6%) | 2.34; [0.82, 6.66]; p=0.1113 |
| *Neisseria gonorrhoeae* | 3/746 (0.4%) | 13/794 (1.6%) | 0.25; [0.07, 0.86]; p=0.0284 |
| *Treponema palladium* | 7/751 (0.9%) | 16/797(2.0%) | 0.46; [0.24, 0.88]; p=0.0188 |
| *Trichomonas vaginalis* | 88/1068 (8.2%) | 122/1143 (10.7%) | 0.77; [0.59, 1.00]; p=0.0527 |
| Bacterial vaginosis | 64/746 (8.6%) | 94/794 (11.8%) | 0.73; [0.54, 0.98]; p=0.0384 |
| Ophthalmia neonatorum^b^ | 4/1140 (0.4%) | 2/1190 (0.2%) | 2.09; [0.38, 11.38]; p=0.3942 |
| Pneumonia and other lower respiratory tract infections | 7/1445 (0.5%) | 18/1445 (1.3%) | 0.39; [0.16, 0.93]; p=0.0332 |
|  | **AZCQ**  **mean (SD)** | **SP**  **mean (SD)** | **Difference (AZCQ-SP)**  **LS mean; [95% CI]; *p* value** |
| Number of episodes of STIs per study participant from first dose to delivery | 0.14 (0.40) | 0.19 (0.47) | −0.05; [−0.08, −0.02]; *p*=0.0011 |

Statistically significant findings are highlighted in grey.

^a^Denominators are the number of subjects with available measurements.

^b^Denominators are the number of subjects with a premature or full-term live birth.
