# Supplementary material for: Efficacy and Safety of Azithromycin-Chloroquine versus Sulfadoxine-Pyrimethamine for Intermittent Preventive Treatment of Plasmodium falciparum Malaria Infection in Pregnant Women in Africa: An Open-Label, Randomized Trial
Source: PLoS One. 2016 Jun 21;11(6):e0157045. doi: 10.1371/journal.pone.0157045 (PMC4915657; doi:10.1371/journal.pone.0157045)
Supplement: S6 Table — (DOCX) [file pone.0157045.s007.docx]

**S6 Table. Nasopharyngeal swaps positive for macrolide- and penicillin-resistant *S. pneumoniae***

|  | **AZCQ**  **n/N (%)** | **SP**  **n/N (%)** |
| --- | --- | --- |
| Day 28 post delivery |  |  |
| Total number of study participants tested | 551 | 569 |
| Positive for *S. pneumoniae* | 8 | 17 |
| Positive for macrolide-resistant *S. pneumoniae* | 0/8 (0%) | 2/17 (11.8%) |
| Positive for penicillin-resistant *S. pneumoniae* | 0/8 (0%) | 0/17 (0%) |
| About 6 months after last IPTp dose |  |  |
| Total number of study participants tested | 478 | 489 |
| Positive for *S. pneumoniae* | 16 | 11 |
| Positive for macrolide-resistant *S. pneumoniae* | 0/16 (0%) | 0/11 (0%) |
| Positive for penicillin-resistant *S. pneumoniae* | 0/16 (0%) | 0/11 (0%) |
