# Supplementary material for: Efficacy and Safety of Azithromycin-Chloroquine versus Sulfadoxine-Pyrimethamine for Intermittent Preventive Treatment of Plasmodium falciparum Malaria Infection in Pregnant Women in Africa: An Open-Label, Randomized Trial
Source: PLoS One. 2016 Jun 21;11(6):e0157045. doi: 10.1371/journal.pone.0157045 (PMC4915657; doi:10.1371/journal.pone.0157045)
Supplement: S2 Text — (PDF) [file pone.0157045.s009.pdf]

CLINICAL PROTOCOL

A PHASE 3, OPEN LABEL, RANDOMIZED, COMPARATIVE STUDY TO EVALUATE AZITHROMYCIN PLUS CHLOROQUINE AND SULFADOXINE PLUS PYRIMETHAMINE COMBINATIONS FOR INTERMITTENT PREVENTIVE TREATMENT OF FALCIPARUM MALARIA INFECTION IN PREGNANT WOMEN IN AFRICA

Compound:	Azithromycin Dihydrate and Chloroquine Phosphate
Compound Name (if applicable):	Azithromycin/Chloroquine Combination
US IND Number (if applicable):	N/A
Protocol Number:	A0661158
Phase:	Phase 3
Version and Date:	Final Protocol: 14 February 2010

This document contains confidential information belonging to Pfizer. Except as otherwise agreed to in writing, by accepting or reviewing this document, you agree to hold this information in confidence and not copy or disclose it to others (except where required by applicable law) or use it for unauthorized purposes. In the event of any actual or suspected breach of this obligation, Pfizer must be promptly notified.

090177e1811dafca\Approved\Approved On: 22-Feb-2010 14:41

PROTOCOL SUMMARY

Indication

Intermittent preventive treatment of *falciparum* malaria in pregnant women (IPTp).

Background and Rationale

Malaria is a serious protozoal infection caused by any of five species of *Plasmodia* and transmitted by anopheline mosquitoes; it continues to be one of the largest global health problems.[1, 2] As per the World Health Organization (WHO) *World Malaria Report* 2009,[3] about half of the world's population is at risk of infection in 108 endemic countries and territories. In 2008, there were an estimated 243 million cases and 863,000 malaria-related deaths.[3] Malaria in pregnancy (MIP) is one of the most common causes of preventable mortality and morbidity in pregnant women and infants in sub-Saharan Africa. An estimated 30 million pregnancies are at risk of malaria infection in sub-Saharan Africa each year;[4] about 200,000 infants and 10,000 women die of malaria in pregnancy each year.[5]

MIP is associated with suboptimal pregnancy outcomes including low birth-weight (LBW, <2,500 g) neonates and is caused by pre-term delivery, intrauterine growth-retardation, or both.[6, 7] MIP is also associated with adverse events including still births and miscarriages.[8] LBW is the most predictive risk factor for neonatal mortality in sub-Saharan Africa. LBW newborns who survive infancy have more episodes of diarrheal diseases and respiratory infections than children born above the LBW threshold.[7-9] In turn, these LBW infants are also more likely to have micro-vascular conditions later in life including hypertension, obesity, and Type-2 diabetes.[10-13] LBW girls have a higher risk as adults of developing pre-eclampsia and delivering LBW infants.[14] Thus reducing the incidence of LBW has broad public health implications that are life-long and inter-generational.

Important progress has been made in the control of malaria in pregnancy in Africa with the introduction of an intermittent preventive treatment for malaria in pregnancy (IPTp). The WHO recommends the administration of IPTp during antenatal visits in high malaria transmission area of sub-Saharan Africa.[11] However, sulfadoxine-pyrimethamine (SP) resistance has become widespread especially in East and Southern Africa greatly limiting the protective effect of IPTp. The development of safe, efficacious and affordable replacement of SP for IPTp is an urgent priority.

The combination of azithromycin (AZ) and chloroquine (CQ) could potentially replace SP for IPTp. AZ and CQ are synergistic *in vitro* and *in vivo* against CQ resistant strains of *P. falciparum*. Co-administration of AZ and CQ has demonstrated efficacy, safety and tolerability in two multi-country Phase 3 clinical studies (A0661134 and A0661155) in the treatment of symptomatic uncomplicated malaria in non-pregnant adults in sub-Saharan Africa. AZ and CQ have been on the market for several years and have extensive safety records in adults, children and pregnant women. Both AZ and CQ have also been widely used in all trimesters of pregnancy and are considered safe in pregnant women as individual agents. AZ is used to treat and prevent sexually transmitted infections (STIs) including *Treponema pallidum* (syphilis), *Neisseria gonorrhoeae* and *Chlamydia trachomatis*

090177e1811dafca\Approved\Approved On: 22-Feb-2010 14:41

infections. The incidence of adverse fetal outcomes has been reduced by about 30% when these three STIs were treated in pregnancy.[30] Hence, activity of AZ against these infections should provide additional benefit. AZ has also demonstrated protective effect against *Trichomonas vaginalis* when used as chemoprophylaxis[31] and reduces the risk of preterm delivery attributable to *T.* vaginalis, even in the second trimester when first-line treatment, metronidazole, should be avoided.[32] CQ, having anti-inflammatory properties,[97] has been shown to reduce systolic pressure and increase blood flow,[98] offering potential protection against pre-eclampsia.

A fixed dose combination tablet formulation of AZ and CQ (AZCQ - 250 mg AZ/155 mg CQ base) has been developed specifically for the IPTp indication and will be evaluated in this study. In a clinical pharmacology study A0661186, AZCQ administration resulted in the systemic exposures to AZ, CQ and des-ethyl CQ (an active metabolite of CQ) at levels comparable to those achieved following administration of commercial standalone tablets of AZ (Zithromax) and CQ (Aralen) evaluated in adult treatment studies.

This study is a Phase 3 clinical trial aimed at demonstrating superiority of AZCQ over SP, the current standard of care for IPTp indication. It will be conducted primarily in East and Southern Africa where SP resistance is evident. The study will be conducted in asymptomatic pregnant subjects enrolled during second trimester of pregnancy, and about half of the subjects will be primigravidae and secundigravidae pregnant women. Each subject will receive three IPTp courses of AZCQ or SP during antenatal care (ANC) visits at 4 - 8 week intervals. Subjects will be followed up at delivery or within 2 days of subject reporting home delivery (within 24 hours of delivery), and on Day 28 (window: Day 28 - Day 42) post-delivery. This will be the pivotal study for regulatory submissions to European Medicines Agency (EMA) and to the national regulatory agencies in sub-Saharan African countries.

Objectives

The primary objective is to establish superiority of AZCQ over SP in protective efficacy for IPTp as measured by the proportion of subjects with sub-optimal pregnancy outcome.

Key secondary objectives include comparison of IPTp regimens of AZCQ and SP in:

1. Proportion of subjects with LBW (<2500 g) live neonates;
2. Proportion of subjects with severe anemia (hemoglobin <8 g/dL);
3. Proportion of subjects with anemia (hemoglobin <11 g/dl);
4. Proportion of subjects with placental parasitemia;
5. Occurrence of sexually transmitted infections (STIs); and
6. Safety and tolerability of the two treatment regimens.

090177e1811dafca\Approved\Approved On: 22-Feb-2010 14:41

Study Design

This is a Phase 3, open label, randomized, parallel group study that will compare the efficacy of IPTp regimens of AZCQ and SP in asymptomatic pregnant subjects enrolled during second trimester of pregnancy. About half of the subjects will be primigravidae and secundigravidae pregnant women since they have a higher risk for suboptimal pregnancy outcomes due to malaria. They will be randomized to receive either AZCQ or SP after informed consent is obtained (and assent if applicable) and inclusion/exclusion criteria are determined to have been met. Each subject will receive three IPTp courses of AZCQ or SP during antenatal care (ANC) visits at 4 - 8 week intervals. The first treatment course will be administered during the second trimester (14-26 weeks of gestation as confirmed by ultrasound). The last treatment course should be given to subjects prior to or during Week 36 of gestation. Subjects will be followed up at delivery or within 2 days of subject reporting home delivery (report within 24 hours when it occurs), and on Day 28 (window: Day 28 - Day 42) post-delivery. Long lasting insecticidal-treated bednets (LLINs) will be given to all subjects on Day 0 at Visit 1 of the study with the instructions to use them; the installation of LLINs will be verified during the first home visits (Day 1 or Day 2) of Treatment Course 1 by fieldworker(s).

Study Treatments

The study treatment is a fixed dose tablet formulation of AZ and CQ (AZCQ) containing 250 mg AZ and 155 mg CQ base. Comparator drug is SP and will be supplied as Fansidar® (Roche) tablet (500 mg sulfadoxine/25 mg pyrimethamine). Subjects will be randomized in 1:1 ratio to receive either AZCQ or SP IPTp regimens. Both regimens will consist of three treatment courses. The treatment courses of AZCQ and SP will consist of the following:

1. **AZCQ treatment course**: 1000 mg AZ and 620 mg of CQ base (4 combination tablets of AZCQ; individual strength of 250mg/155mg), *per os* (PO, orally) once daily for 3 days (Days 0, 1, 2);

OR

2. **SP treatment course:** 1500 mg sulfadoxine and 75 mg pyrimethamine (3 fixed tablets of SP strength at 500mg/25mg), PO single dose once daily on Day 0.

Each subject will receive three IPTp treatment courses of AZCQ or SP during ANC visits at 4 - 8 week intervals. The first treatment course will be administered during the second trimester (14-26 weeks of gestation as confirmed by ultrasound). The last treatment course should be given to subjects prior to or during 36 weeks of gestation.

The first dose of each AZCQ treatment course will be administered under supervision by investigators during the ANC visits. The subsequent two doses of AZCQ will be administered at home under supervision by the fieldworker(s).

Each dose of SP treatment will be administered under supervision during the ANC visits.

090177e1811dafca\Approved\Approved On: 22-Feb-2010 14:41

All study drugs will be administered as open therapy under supervision.

Main Endpoints

Primary Efficacy Endpoint

- Presence of subjects with a sub-optimal pregnancy outcome defined as any of the following: live-borne neonate (singleton) with low birth-weight (or LBW for short, defined as live birth weight <2,500g), premature birth (<37 weeks), abortion (≤28 weeks), still birth (>28 weeks), lost to follow-up prior to termination of pregnancy or delivery, or missing birth weight of the neonates.

Key Secondary Efficacy Endpoints

1. Occurrence at birth of a LBW live neonate;
2. Occurrence of severe maternal anemia (Hb <8 g/dL) at 36-38 weeks of gestation;
3. Occurrence of anemia (Hb <11 g/dL) at 36-38 weeks of gestation;
4. Occurrence of placental parasitemia at delivery;
5. Occurrence of placental malaria as determined by histology;
6. Number of episodes of STIs per subject including *T. pallidum*, *N. gonorrhoeae*, *C. trachomatis*, during the study period following first dose (diagnosis based on clinical presentation any time from first IPTp dose to delivery and/or on laboratory test results between Weeks 36-38).

Other Secondary Efficacy Endpoints

1. Hemoglobin concentration at 36-38 weeks of gestation;
2. Occurrence at birth of a neonates with congenital abnormalities;
3. Occurrence of a perinatal or neonatal death;
4. Birth weight of the live-borne neonate (singleton);
5. Number of episodes of symptomatic malaria per subject anytime from first IPTp dose administration to delivery;
6. Occurrence of a subject requiring additional treatment for symptomatic malaria during the study period following the first dose (diagnosed based on clinical presentation and/or lab test results);
7. Occurrence of peripheral parasitemia at 36-38 weeks of gestation;

090177e1811dafca\Approved\Approved On: 22-Feb-2010 14:41

8. Occurrence of peripheral parasitemia at delivery;
9. Occurrence of cord blood parasitemia at delivery;
10. Occurrence of STIs including *T. pallidum, N. gonorrhoeae, C. trachomatis* during the study period following first dose (diagnosed based on clinical presentation prior to Week 36-38 and/or lab test results between Week 36-38 of gestation);
11. Occurrence of a positive result for *C. trachomatis* infection at 36-38 weeks of gestation (diagnosed based on lab result);
12. Occurrence of a positive result for *N. gonorrhoeae* infection at 36-38 weeks of gestation (diagnosed based on lab result);
13. Occurrence of a positive resultfor *T. pallidum* test at 36-38 weeks of gestation (diagnosed based on lab result);
14. Occurrence of a *T. vaginalis* infection at 36-38 weeks of gestation (diagnosed based on lab result);
15. Occurrence of bacterial vaginosis at 36-38 weeks of gestation (diagnosed based on lab result);
16. Occurrence of ophthalmia neonatorum (diagnosed based on lab test results) in the neonate;
17. Occurrence of bacterial infections including pneumonia and other lower respiratory tract infections anytime from first IPTp dose administration to delivery;
18. Occurrence of pre-eclampsia from Week 20 to delivery;
19. Occurrence of nasopharyngeal swabs positive for macrolide resistant and penicillin resistant *Streptococcus pneumoniae* at baseline, at Day 28 (window Day 28 - Day 42) post delivery, and at about 6 months following last IPTp course. This test will be done in about 600 subjects each from the AZCQ and SP arms from two or more sites.

Safety Endpoints

- Safety and tolerability will be assessed by spontaneously reported adverse events, vital signs, physical examination, laboratory tests including hemoglobin and urine test for glucose and protein, and pregnancy outcomes for mothers and by the general physical examination for the neonates through Day 28 (window Day 28 – Day 42) post delivery.

090177e1811dafca\Approved\Approved On: 22-Feb-2010 14:41

090177e1811dafca\Approved\Approved On: 22-Feb-2010 14:41

Outcome Research Endpoints

1. Number of times a newborn/infant is taken to a local health clinic/physician's office/outpatient hospital clinic including emergency room (without having to be admitted) during the first 28 days (window Day 28 - Day 42) of life;

2. Number of times a newborn/infant is admitted to a hospital during the first 28 days (window Day 28 - Day 42) of life;

3. Number of times a mother is taken to a local health clinic/physician's office/outpatient hospital clinic including Emergency Room (without having to be admitted) from the time of the administration of the first dose of IPTp through Visit 6 on Day 28 (window Day 28 - Day 42) post delivery;

4. Number of times a mother is admitted to a hospital from the time of the administration of the first dose of IPTp through Visit 6 on Day 28 (window Day 28 - Day 42) post delivery.

Statistical Methods

Sample Size

One blinded sample size assessment will be performed when approximately 1,000 subjects achieve the primary endpoint. The sample size will range between 2,602 and 5,044. Due to the adaptive nature of the sample size determination, operating characteristics of the study design in terms of power and Type I error rate were evaluated using simulations. The design assumed a 20% risk reduction in the primary endpoint of proportion of subjects with sub-optimal pregnancy outcomes (relative risk =0.80, AZCQ/SP), alpha =0.000625 1-sided, and provides approximately 90% power. The secondary endpoints such as low birth weight will use an alpha=0.025 1-sided. See Section 9.1 for details.

Statistical Analysis

The primary efficacy endpoint of the trial is the presence of subjects with a sub-optimal pregnancy outcome defined as any of the following: live newborn (singleton) with low birth-weight (or LBW, defined as live birth weight <2,500 g), premature birth (<37 weeks), abortion (≤28 weeks), still birth (>28 weeks), lost to follow-up prior to termination of pregnancy or delivery, or missing birth weight of the neonate. The primary analysis set will be the intent-to-treat (ITT), and will exclude multiple gestations. Secondary analyses will also be repeated using the per protocol analysis set, also excluding multiple gestations.

The Mantel-Haenszel estimate of the common risk ratio will be computed using SAS Proc Freq, adjusted for the randomization strata. The risk ratio will be the proportion of AZCQ subjects with suboptimal pregnancy outcome over the proportion of SP subjects with sub-optimal pregnancy outcome. The p-value will be calculated using the Wald test statistic on the natural log risk ratio scale.

All secondary efficacy endpoints that are dichotomous, expressed as a proportion, will be summarized and analyzed using the same methods as for the primary endpoint. Secondary efficacy endpoints that are not dichotomous will be analyzed using analysis of covariance or analysis of variance.

Interim Analysis

Unblinded sequential analyses will be performed following completion of the primary endpoint (pregnancy outcome) assessment at 50%, 70%, and 100% (final analysis) of the accrued number of subjects.

Independent Data Monitoring Committee (IDMC)

The IDMC constituted for IPTp will review safety data at regular intervals to be determined at the time of IDMC charter development.

090177e1811dafca\Approved\Approved On: 22-Feb-2010 14:41

Study Schematic

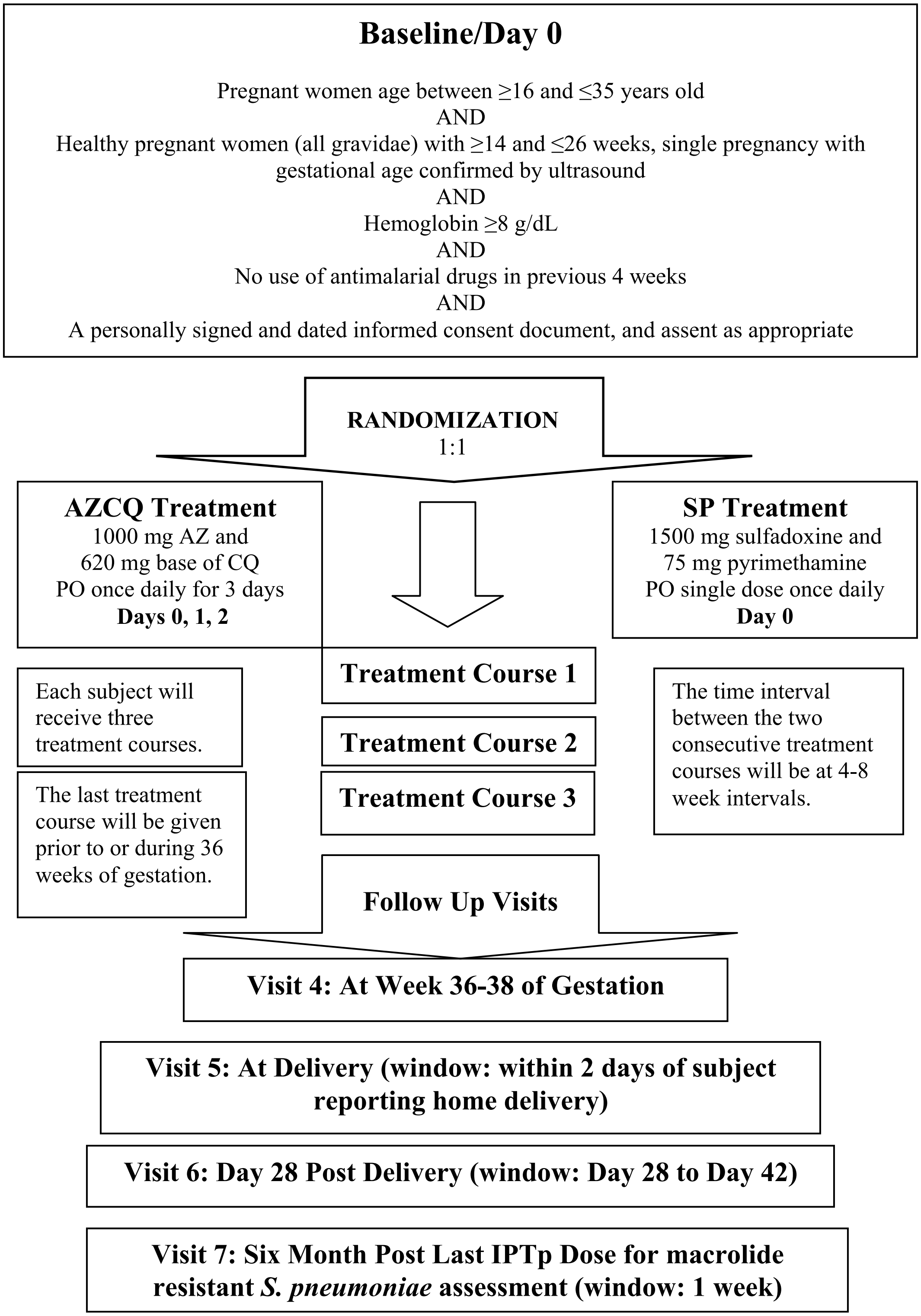

090177e1811dafca\Approved\Approved On: 22-Feb-2010 14:41

090177e1811dafca\Approved\Approved On: 22-Feb-2010 14:41

SCHEDULE OF ACTIVITIES

Protocol Activity	Visit 1			Visit 2			Visit 3			Visit 4	Visit 5	Visit 6	Visit 7
	Treatment Course 1[a]			Treatment Course 2[a]			Treatment Course 3[a]			Follow Up Visits			
	Day 0[b, c]	Day 1[b]	Day 2[b]	Day 0[b]	Day 1[b]	Day 2[b]	Day 0[b]	Day 1[b]	Day 2[b]	Weeks 36-38 of gestation	At Delivery[d]	Day 28 Post Delivery[e]	6 month post last IPTp dose[f]
**Informed Consent Process**	X												
**Medical History**	X												
**Obstetrical History**	X												
**Assess Concomitant Treatment**	X			X			X			X	X	X	X
**Complete Physical Exam[g]**	X												
**Limited Physical Exam[h]**											X	X	X
**Vital Signs**	X			X			X			X	X	X	X
**Routine Obstetric Checkup**	X			X			X			X			
**Ultrasound[i]**	X												
**Laboratory**													
Peripheral Blood Smears										X	X[t]		
Hemoglobin (Hb)	X			X			X			X	X[t]	X	
Urine Pregnancy Test[j]	X												
Urine tests for glucose & protein	X			X			X			X			
**Randomize Subjects**	X												
**Administer Treatment**													
Azithromycin-chloroquine (AZCQ)[k]	X	X	X	X	X	X	X	X	X				
Sulfadoxine-pyrimethamine (SP)[l]	X			X			X						
**Distribute long lasting insecticidal-treated bednets (LLINs)**	X												
**Confirm the installation of LLINs at home by fieldworkers[m]**		X[m]	X[m]										
**Molecular Testing**													
Genetic Marker for CQ Resistance[n]										X[n]			
Genetic Markers for SP Resistance[o]										X[o]			

090177e1811dafca\Approved\Approved On: 22-Feb-2010 14:41

Protocol Activity	Visit 1			Visit 2			Visit 3			Visit 4	Visit 5	Visit 6	Visit 7
	Treatment Course 1[a]			Treatment Course 2[a]			Treatment Course 3[a]			Follow Up Visits			
	Day 0[b, c]	Day 1[b]	Day 2[b]	Day 0[b]	Day 1[b]	Day 2[b]	Day 0[b]	Day 1[b]	Day 2[b]	Weeks 36-38 of gestation	At Delivery[d]	Day 28 Post Delivery[e]	6 month post last IPTp dose[f]
**Reproductive Tract Infections (RTIs) Testings**													
Treponima pallidum Tests	X[p]									X			
Chlamydia trachomatis Test										X			
Neisseria gonorrhoeae Test										X			
Trichomonas vaginalis										X			
Bacterial Vaginosis										X			
Ophthalmia neonatorum[q]											X		
**Macrolide and Penicillin Resistance Test for *Strep. Pneumonia*[r]**	X											X	X
**Adverse Events Reporting[s]**	X	X	X	X	X	X	X	X	X	X	X	X	X
**Placental Blood Smear[t]**											X[t]		
**Placental Histology[t, u]**											X[t]		
**Cord Blood Smear[t]**											X[t]		
**Pregnancy Outcome**											X		
**Neonate Weight**											X	X	
**General Physical Exam of Neonate[v]**											X	X	
**Outcome Research/Resource Utilization Questions**											X	X	

a. Each subject will receive 3 IPTp treatment courses of AZCQ or SP during antenatal care (ANC) visits at 4 - 8 week intervals. The first treatment course will be administered during the second trimester (14-26 weeks of gestation as confirmed by ultrasound). The last treatment course should be given to subjects prior to or during 36 weeks of gestation.

b. The day of visit for the specified treatment course.

c. All tasks should be performed prior to the first dose except AE assessment and bed net distribution.

d. Every attempt should be made to follow up the subject at delivery. In the case of home delivery, the subject should report delivery within 24 hours of delivery. Fieldworker should visit the subject at home thereafter within 2-days of subject reporting delivery.

e. Day 28 post delivery: window Day 28- Day 42 post delivery.

f. Only for a limited number of subjects who participate in macrolide and penicillin resistance test for *Strep. Pneumoniae*. Window: 1 week from 6 months post last IPTp course.

090177e1811dafca\Approved\Approved On: 22-Feb-2010 14:41

g. Complete physical exam: general appearance, throat, neck, thyroid, musculoskeletal, skin, lymph nodes, extremities, pulses, pulmonary, cardiac, abdominal, and neurological examination.

h. Limited physical exam: general appearance, brief pulmonary, cardiac, abdominal and neurological examination.

i. Ultrasound will be performed only if the urine pregnancy test is positive.

j. If not done at any prior ANC visit for this pregnancy.

k. For subjects on AZCQ arm only. The first dose of treatment is administered under supervision during the ANC visit. The subsequent two doses following each ANC visit will be administered at home under supervision of fieldworkers. If a subject is withdrawn from the AZCQ IPTp regimen, she will be switched to the standard IPTp regimen as per local guidelines and will undergo regular follow up visits. If she receives an SP IPTp course during the study, she will be subsequently followed up as per follow up schedule for subjects receiving SP IPTp regimen.

l. For subjects on SP arm only. Each treatment is administered under supervision during the ANC visit.

m. The installation of LLINs will be confirmed during home visits Days 1 or 2 of Visit 1 by fieldworker(s): on Day 1 or Day 2 for subjects on AZCQ arm; and on Day 2 for subjects on SP arm.

n. Only at Week 36-38 if positive for parasitemia on AZCQ arm only.

o. Only at Week 36-38 if positive for parasitemia on SP arm only.

p. Only if not done at the previous ANC check up. The test will be offered in consistent with local ANC guidelines.

q. Laboratory diagnosis of ophthalmia neonatorum: A sample of **c**onjunctival exudates from neonates with purulent discharge will be collected prior to giving the standard presumptive treatment/prophylaxis for ophthalmia neonatorum. This treatment will be given to neonates at birth per national/local treatment guidelines.

r. Nasopharyngeal swabs will be collected in about 600 subjects each from the AZCQ and SP arms from two or more sites (1) at baseline, (2) at Day 28 - Day 42 post delivery, and (3) at about 6 months post last IPTp course.

s. AE assessment will be conducted on Day 0 and Day 2 of Visit 1, 2, and 3 for subjects in SP arm: Day 0 assessments will be done at the Antenatal visit and Day 2 assessment will be done at home by the fieldworker.

t. Possible only if hospital delivery.

u. A sample of placenta tissue (size: about 2cm x 2cm x 1cm) will be collected from the subjects who deliver at hospital as feasible.

v. Physical exam of neonate: weight, length, head circumference, APGAR score, Ballard score, congenital anomaly, and complications at birth and up to six weeks of life if applicable.

TABLE OF CONTENTS

1. INTRODUCTION 17
1.1. Indication 17
1.2. Background 17
1.3. Azithromycin 20
1.3.1. Non-Clinical Summary of Azithromycin 20
1.3.1.1. Pharmacology and Microbiology of Azithromycin 20
1.3.1.2. Non-clinical Activity of Azithromycin as an Antimalarial Agent 20
1.3.1.3. Non-clinical Safety of Azithromycin 21
1.3.2. Clinical Summary of Azithromycin 21
1.3.2.1. Clinical Pharmacokinetics of Azithromycin 21
1.3.2.2. Clinical Efficacy of Azithromycin Monotherapy in Malaria 23
1.3.2.3. Clinical Effects of Azithromycin against Sexually Transmitted Infections 23
1.3.2.4. Clinical Safety and Tolerability of Azithromycin 24
1.4. Chloroquine 26
1.4.1. Non-Clinical Summary of Chloroquine 26
1.4.1.1. Pharmacology and Microbiology of Chloroquine 26
1.4.1.2. Non-clinical Safety of Chloroquine 26
1.4.2. Clinical Summary of Chloroquine 26
1.4.2.1. Clinical Pharmacology of Chloroquine 26
1.4.2.2. Clinical Efficacy of Chloroquine Monotherapy in Malaria 27
1.4.2.3. Other Potential Clinical Benefits of Chloroquine 28
1.4.2.4. Clinical Safety and Tolerability of Chloroquine 28
1.5. Azithromycin-Chloroquine Combination (AZCQ) 29
1.5.1. Non-Clinical Summary Information 29
1.5.1.1. Non-Clinical Efficacy of the Azithromycin and Chloroquine Combination against *P. falciparum* 29
1.5.1.2. Non-Clinical Safety of Azithromycin and Chloroquine Combination 29
1.5.2. Clinical Summary Information for Azithromycin and Chloroquine Combination 30
1.5.2.1. Clinical Pharmacology of Azithromycin and Chloroquine Combination 30
1.5.2.2. Clinical Efficacy of Azithromycin-Chloroquine Combination against *P. falciparum* 31
1.5.2.3. Clinical Safety and Tolerability of Azithromycin and Chloroquine Combination 32
1.5.2.4. Clinical Safety of Azithromycin and Chloroquine Combination in Pregnancy 32
1.6. Rationale for AZCQ in IPTp 33
1.6.1. Dosing Regimen Selection 34
1.6.1.1. Sulfadoxine-Pyrimethamine (SP) as Comparator 34

090177e1811dafca\Approved\Approved On: 22-Feb-2010 14:41

1.6.1.2. Azithromycin-Chloroquine (AZCQ) 34
2. STUDY OBJECTIVES AND ENDPOINTS....35
2.1. Objectives....35
2.2. Endpoints....35
3. STUDY DESIGN....38
4. SUBJECT SELECTION....38
4.1. Inclusion Criteria....38
4.2. Exclusion Criteria....39
4.3. Randomization Criteria....40
4.4. Life Style Guidelines....40
5. STUDY TREATMENTS....41
5.1. Allocation to Treatment....41
5.2. Breaking the Blind....42
5.3. Drug Supplies....42
5.3.1. Formulation and Packaging....42
5.3.2. Preparation and Dispensing....42
5.3.3. Administration....42
5.3.4. Compliance....43
5.4. Drug Storage and Drug Accountability....43
5.5. Concomitant Medication(s)....44
5.6. Use of Anti-Malarial Treatment for Malaria in Pregnancy....44
5.7. Treatment for Sexually Transmitted Infection....45
5.8. Treatment for Anemia in Pregnancy....45
6. STUDY PROCEDURES....45
6.1. Study Period....45
6.1.1. Visit 1: Baseline/Screening Visit/Treatment Course 1....45
6.1.2. Visit 2: Treatment Course 2....46
6.1.3. Visit 3: Treatment Course 3....47
6.1.4. Visit 4: Follow-up Visit at Weeks 36 to 38 of Gestation....48
6.1.5. Visit 5: Follow-up Visit at Delivery....49
6.1.5.1. Follow Up Visit at Birth in Case of Hospital Delivery (At Birth)....49
6.1.5.2. Follow Up Visit at Birth in Case of Home Delivery (Within 2 Days of Subject Reporting Delivery)....50
6.1.6. Visit 6: Follow-up Visit on Day 28 Post Delivery (Window: Day 28 to Day 42)....50
6.1.7. Follow-up Visit at About 6 Months after Last IPTp Course (in a Limited Number of Subjects Only)....51
6.1.8. Unscheduled Visit....51
6.2. Subject Withdrawal....51

090177e1811dafca\Approved\Approved On: 22-Feb-2010 14:41

7. ASSESSMENTS....52
7.1. Safety Assessments: 52
7.2. Efficacy Assessments 53
7.3. Outcome Research Assessment 56
8. ADVERSE EVENT REPORTING....57
8.1. Adverse Events 57
8.2. Reporting Period....57
8.3. Definition of an Adverse Event 57
8.4. Abnormal Test Findings 58
8.5. Serious Adverse Events 58
8.6. Hospitalization....59
8.7. Severity Assessment 60
8.8. Causality Assessment 60
8.9. Exposure During Pregnancy 61
8.10. Withdrawal Due to Adverse Events (See also Section on Subject Withdrawal)....61
8.11. Eliciting Adverse Event Information....62
8.12. Reporting Requirements 62
8.12.1. Serious Adverse Event Reporting Requirements 62
8.12.2. Non-Serious Adverse Event Reporting Requirements....63
8.12.3. Medical Device Complaint Reporting Requirements 63
8.12.4. Sponsor Reporting Requirements to Regulatory Authorities....63
9. DATA ANALYSIS/STATISTICAL METHODS 63
9.1. Sample Size and Power Determination 63
9.1.1. Simulated Power Under a 20% Risk Reduction 64
9.2. Efficacy Analysis....65
9.2.1. Analysis of Primary Endpoint....66
9.2.2. Analysis of Secondary Endpoints 67
9.3. Outcome Research Analysis 67
9.4. Safety Analysis 68
9.5. Interim Analysis 68
9.6. Independent Data Monitoring Committee (IDMC)....69
10. QUALITY CONTROL AND QUALITY ASSURANCE....69
11. DATA HANDLING AND RECORD KEEPING 69
11.1. Case Report Forms/Electronic Data Record....69
11.2. Record Retention 70
12. ETHICS....70
12.1. Institutional Review Board (IRB)/Independent Ethics Committee (IEC)....70
12.2. Ethical Conduct of the Study....71

090177e1811dafca\Approved\Approved On: 22-Feb-2010 14:41

12.3. Subject Information and Consent/Assent 71
12.4. Reporting of Safety Issues and Serious Breaches of the Protocol or ICH GCP 71
13. DEFINITION OF END OF TRIAL 72
13.1. End of Trial in all Participating Countries 72
14. SPONSOR DISCONTINUATION CRITERIA 72
15. PUBLICATION OF STUDY RESULTS 72
15.1. Communication of Results by Pfizer 72
15.2. Publications by Investigators 73
16. REFERENCES 75

TABLES

Table 1. Azithromycin Pharmacokinetic Data 22
Table 2. PK Parameters from A0661186 Study Formulation 30
Table 3. Severity Assessment 60
Table 4. Sample Size Determination 64
Table 5. Power and Futility Probability: 20% Risk Reduction 64
Table 6. Type I Error Rate 65

FIGURES

Figure 1. Predicted Azithromycin Serum Concentrations in Healthy Subjects and Pregnant women 22

090177e1811dafca\Approved\Approved On: 22-Feb-2010 14:41

1. INTRODUCTION

1.1. Indication

Intermittent Preventive Treatment of *falciparum* malaria in pregnant women (IPTp).

1.2. Background

Malaria is a serious protozoal infection caused by any of five species of *Plasmodium* and transmitted by anopheline mosquitoes. Malaria caused by *Plasmodium falciparum* is one of the leading causes of deaths caused by a single causative agent.[1, 2] As per the World Health Organization (WHO) *World Malaria Report* 2009,[3] about half of the world's population is at risk of infection in 108 endemic countries and territories. In 2008, there were an estimated 243 million cases and 863,000 malaria-related deaths.[4] Africa has the largest number of people living in high-risk areas and the most the malaria-related deaths. Pregnant women are more prone to malaria than non-pregnant women, especially during their first and second pregnancies. Malaria in pregnancy (MIP) is one of the most common causes of preventable morbidity and mortality in pregnant women and infants in sub-Saharan Africa. An estimated 30 million pregnancies are at risk of malaria infection in the region each year;[4] about 200,000 infants and 10,000 women die of malaria in pregnancy each year.[5]

MIP is associated suboptimal pregnancy outcomes including low birth-weight (LBW, <2,500 g) neonates and is caused by pre-term delivery, intrauterine growth-retardation, or both.[6, 7] MIP is also associated with adverse events including still births, and miscarriages.[8] LBW is the most predictive risk factor for neonatal mortality in sub-Saharan Africa. LBW newborns who survive infancy have more episodes of diarrhea disease and respiratory infection than children born above the LBW threshold.[7-9] In turn, these LBW infants are also more likely to have micro-vascular conditions later in life including hypertension, obesity, and Type-2 diabete mellitus.[10-13] LBW girls have a higher risk as adults of developing pre-eclampsia and delivering LBW infants.[14] Thus reducing the incidence of LBW has broad public health implications that are life-long and inter-generational.

Intermittent Preventive Treatment in Pregnancy (IPTp) is one of the four key strategies recommended by the WHO's Global Malaria Program for malaria control in high transmission areas, where approximately 1 in 4 pregnant women are parasitemic at any given time, but remain typically asymptomatic.[15] Placental infection is of particular concern to fetal development and can only be diagnosed post-partum by placental histological examination. IPTp is based on administration of a complete curative dosing regimen of an antimalarial medicine at periodic intervals during pregnancy regardless of whether or not pregnant women are infected with malaria. IPTp has two primary objectives: (1) to clear asymptomatic peripheral and placental parasitemia, and (2) to provide intermittent chemoprophylaxis against malaria infection during pregnancy.[4]

The WHO recommends use of IPTp in endemic areas of sub-Saharan Africa with two or three (in HIV infected pregnant women) courses of sulfadoxine and pyrimethamine (SP) after fetal quickening in second trimester with each course given no less than one month apart, and all prior to the last month of pregnancy.[15] As an anti-metabolite, SP is not safe in first

090177e1811dafca\Approved\Approved On: 22-Feb-2010 14:41

trimester of pregnancy. IPTp with SP has been shown to be effective in reducing maternal anemia, placental malaria and low birth weight neonates.[16] It is estimated that universal coverage with IPTp would reduce all-cause neonatal mortality by 32%.[4] Thirty-three African countries have officially adopted IPTp as national policy in their national malaria control programs. The benefits of IPTp are, however, compromised by increasing resistance of *Plasmodium falciparum* to SP especially in East and Southern Africa. The in vivo SP resistance rates have typically been evaluated in terms of treatment failures in clinical trials in children with uncomplicated infection[17] but therapeutic efficacy measured in sick children does not predict efficacy in IPTp with SP. Acquired immunity in adults contributes to the efficacy of partially effective drugs. This protection, however, is not uniform and it is least efficacious among primigravidae who are most vulnerable to the detrimental effects of MIP. It is likely that as rates exceed an undetermined threshold of resistance, SP may fail to provide protection even in multigravidae and hence there is an urgent need to find new, safe and effective drugs or drug combination for IPTp.[4] Also, while SP still seems to work reasonably well in West Africa, evidence suggests that gene-flow will prompt the spread of SP resistance from east to West Africa.[18]

An ideal antimalarial drug or drug combination for IPTp needs to be safe, well tolerated, and efficacious in preventing the detrimental effects of malaria on the mother and the fetus, and, ideally, not used concurrently as the first line treatment for symptomatic malaria. Few clinical trials have attempted to evaluate alternatives to SP in IPTp. Malaria in Pregnancy Consortium is planning to conduct a multi-country clinical trial in East and Southern Africa comparing the IPTp efficacy and safety of SP and mefloquine. Tolerability remains an issue for mefloquine, however, as evidenced by a recent IPTp study in Benin in which 78% ($P<0.001$) of women who received a single dose (15 mg/kg) experienced adverse events including vomiting, dizziness, tiredness, and nausea; one subject had severe neuropsychiatric symptoms.[19] Two retrospective studies found associations between mefloquine exposure and spontaneous abortion,[20] and stillbirth.[21] These results remain unexplained and have not been observed in other studies. In Ghana, the combination of SP and amodiaquine (600 mg on Days 0 and 1, 400 mg Day 2) was recently evaluated for possible use in IPTp; adverse events, most commonly body pains and weakness, dizziness, vomiting and nausea, were observed among 89% ($P=0.001$) of women following the first course.[22] Similar observations of poor amodiaquine tolerability have been reported in other studies.[23, 24]

Other antimalarials that are planned to be investigated for IPTp are SP combinations with azithromycin (AZ) and artesunate. While AZ has been used in all trimesters of pregnancy to treat a variety of infections, there is no evidence of synergy between SP and AZ against *P. falciparum*. Artemisinins are among the most effective and rapidly acting antimalarials to date, but their use in non-clinical studies has been associated with fetal resorptions and embryotoxicity in rodents and rabbits with narrow therapeutic margins when given early in pregnancy.[25] No such evidence in clinical trials among pregnant women has been reported so far. The WHO currently recommends use of artemisinins for treatment of uncomplicated infections in pregnant women only when efficacious alternatives are not available.[26] As for IPTp use, the short half-life of artemisinins may restrict their utility in chemoprophylaxis. Hence, combination of SP and artesunate may not be a viable alternative to SP for IPTp. Artesunate and AZ would not likely be an appropriate combination either, as the combination

090177e1811dafca\Approved\Approved On: 22-Feb-2010 14:41

has produced poor *in vivo* results.[27] This, however, is not unexpected since in vitro pharmacodynamic interaction studies with this combination have also shown that artesunate and AZ are antagonistic to each other.[28] Therefore, options for efficacious, safe and well-tolerated preventive alternatives for pregnant women are limited. The need to develop new therapies is clear and urgent.[29]

The combination of AZ and chloroquine (CQ) could potentially replace SP for IPTp. AZ and CQ combination is synergistic against CQ resistant strains of *P. falciparum* as demonstrated both *in vitro* and *in vivo*. Co-administration of AZ and CQ has demonstrated efficacy, safety and tolerability in two multi-country Phase 3 clinical studies (A0661134 and A0661155) in the treatment of symptomatic uncomplicated malaria in adults in sub-Saharan Africa. AZ and CQ have been on the market for several years and have extensive safety records in adults, children and pregnant women. Both AZ and CQ have been widely used in all trimesters of pregnancy and are considered safe in pregnant women as individual agents. AZ has been used to treat and prevent sexually transmitted infections (STIs) including *Neisseria gonorrhoeae* and *Chlamydia trachomatis* infections. The incidence of adverse fetal outcomes has been shown to have reduced by about 30% when STIs, including the above three STIs and *Trichomonas vaginalis,* were treated in pregnancy.[30] In another study, AZ against these infections should provide additional benefit. In addition, AZ has also demonstrated protective effect against *T. vaginalis* when used for chemoprophylaxis[31] and reduced the risk of preterm delivery attributable to *T. vaginalis,* even in the second trimester when first-line treatment, metronidazole, should be avoided.[32]

A fixed dose combination tablet formulation of AZ and CQ (AZCQ - 250 mg AZ/155 mg CQ base) has been developed specifically for the IPTp indication and will be evaluated in this study. In a clinical pharmacology study A0661186, AZCQ administration resulted in the systemic exposures to AZ, CQ and des-ethyl CQ (an active metabolite of CQ) at levels comparable to those achieved following administration of commercial standalone tablets of AZ (Zithromax) and CQ (Aralen) evaluated in adult treatment studies. Activity of AZ against these common STIs should provide additional benefit in improving pregnancy outcmes.

This study is a Phase 3 clinical trial aimed at demonstrating superiority of AZCQ over SP, the current standard of care for IPTp indication. It will be conducted primarily in East and Southern Africa where SP resistance is evident. Most of the sites will be in areas of high CQ resistance. The study will be conducted in asymptomatic pregnant subjects enrolled during second trimester of pregnancy, and about half of the subjects will be primigravidae and secundigravidae pregnant women. Each subject will receive three IPTp courses of AZCQ or SP during antenatal care (ANC) visits at 4 - 8 week intervals. Subjects will be followed up at delivery or within 2 days of subject reporting home delivery (within 24 hours of delivery), and on Day 28 (window: Day 28 - Day 42) post-delivery. This will be the pivotal study for regulatory submissions to European Medicines Agency (EMA) and to the national regulatory agencies in sub-Saharan African countries.

090177e1811dafca\Approved\Approved On: 22-Feb-2010 14:41

1.3. Azithromycin

1.3.1. Non-Clinical Summary of Azithromycin

1.3.1.1. Pharmacology and Microbiology of Azithromycin

AZ is a slow-acting anti-malarial macrolide,[33] an analogue of erythromycin with a nitrogen atom inserted into the macrolide nucleus. This pharmacological change enables greater penetration of drug into macrophages, fibroblasts and polymorphonuclear neutrophils, and enhanced accumulation within acidified vacuoles, extending the 1.5-hour half-life of erythromycin to 68 hours for AZ.[34] Stable at gastric pH, AZ has an absolute bioavailability of 37% following oral administration.[35] In animal studies, it has been shown to accumulates in hepatic, renal, pulmonary and splenic tissue,[36] and gradually leaches into the bloodstream over a one-week period.[37] Mild renal dysfunction and mild-to-moderate hepatic dysfunction do not affect excretion significantly.

AZ has significant activity against respiratory pathogens, both extracellular (eg, *Streptococcus pneumoniae*, *Haemophilus influenzae*, and *Moraxella catarrhalis*) and intracellular (eg, *Chlamydia pneumoniae*, *Legionella pneumophila*); selected sexually transmitted infections (eg, *Chlamydia trachomatis*, *Neisseria gonorrhoeae, Treponema pallidum,* and *Haemophilus ducreyi*)[38-40] as well as other atypical organisms such as *Bartonella* spp., non-tuberculous mycobacteria, *Rhodococcus equi*, and rickettsiae are susceptible to AZ. AZ also exhibits anti-protozoal activity (eg, *Toxoplasma gondii*, *Giardia. intestinalis*, *Entamoeba histolytica*)[41-43] and anti-paraplasmic activity including *Babesia microti*[44] and *P. falciparum*.

1.3.1.2. Non-clinical Activity of Azithromycin as an Antimalarial Agent

In vitro studies have shown that AZ has a 50% inhibitory concentration (IC_{50}) similar to other antibiotics used against malaria. In an *in vitro P. falciparum* model, AZ did not appear to exhibit cross-resistance with aminoquinolines, artemisinin derivatives, or quinine in an in vitro *P. falciparum* model.[45] The *in vitro* anti-malarial activity of AZ increased 200-fold against *P. falciparum* isolates when incubated between 24 and 48 hours, while its IC_{50} values drop as low as 35 nanomolar.[46] At 48-hours, AZ was 10-fold more active than erythromycin against CQ-resistant *P. falciparum*; the two compounds were equipotent, however, when CQ-sensitive parasites were exposed to the same drug concentration.[47, 48] Yeo and Rieckmann demonstrated that the minimum inhibitory concentrations (MICs) of AZ for both CQ-resistant and sensitive strains decreased over 48 to 96 hours and concluded that it behaved like other antibiotics in its slow rate of action against *P. falciparum*.[49] In India, AZ was tested in vitro against 10 strains of *P. falciparum* (5 CQ-resistant and 5 CQ-sensitive) and completely inhibited parasite growth in all 10 isolates at the highest concentration (40 μg/ml).[50]

In vivo animal studies lend further support to the role of AZ as an antimalarial agent. In the *P.berghei* murine model, AZ was shown to be more active than erythromycin with lower IC50s against both CQ-resistant and sensitive strains.[48] All mice were cured of parasitemia

090177e1811dafca\Approved\Approved On: 22-Feb-2010 14:41

within three days after receiving a dose of AZ that achieved an IC_{50}. AZ was also more active than roxithromycin, clarithromycin, erythromycin and doxycycline in another study using a rodent model with *P. berghei*.[27]

1.3.1.3. Non-clinical Safety of Azithromycin

The non-clinical data for AZ suggest little if any evidence of toxicity. The non-clinical toxicology program included single-dose studies in rats, mice, and dogs, multiple-dose studies of up to 6 months in rats and dogs, fertility and reproductive developmental studies in rats, mice, or rabbits, neonatal studies in rats and dogs, and mechanistic studies in rats and dogs to characterize the extent, reversibility, and consequences of phospholipidosis. Genetic toxicology studies for AZ were also conducted. AZ did not produce toxicity in neonatal rats and dogs at the highest doses ranging from 60 to 140 mg/kg/day that are approximately 5-10 times higher than the human therapeutic dose. Phospholipidosis was observed in tissues of both the neonatal and adult animals though the clinical significance of this finding is unknown.

1.3.1.3.1. Non-clinical Safety of Azithromycin on Reproductive Systems

In fertility/reproductive non-clinical studies, AZ showed no evidence of producing fetal abnormalities in mice, rats, and rabbits at the highest doses ranging from 40 to 200 mg/kg/day. Fetal abnormalities or evidence of embryotoxicity were not observed in mice and rats dosed with AZ at 10, 20, 40, 50, 100, and 200 mg/kg/day (mice dosed on gestation Days 6-13, rats on Days 6-15). A slight delay in fetal ossification was noted when a decrease in maternal body weight occurred at doses of 100 and 200 mg/kg/day at approximately 7-15 times higher than human treatment dose. No evidence of impaired fertility due to AZ was found in rats at 10 mg/kg/day. Rabbits dosed with AZ at 10, 20, and 40 mg/kg/day (on gestation Days 6-18) were not observed with fetal abnormalities or embryotoxicity. There was no evidence of teratogenicity in animal models at four-times the human treatment dose.[51-53]

1.3.2. Clinical Summary of Azithromycin

1.3.2.1. Clinical Pharmacokinetics of Azithromycin

The pharmacokinetics of AZ has been evaluated in several clinical pharmacology studies. Following oral administration of AZ tablets to healthy subjects, peak serum concentrations are achieved between 2 to 3 hours. AZ is widely distributed throughout the body with markedly higher and sustained exposure in tissues than in systemic circulation (up to 100 times of corresponding plasma or serum concentration) indicating that AZ is heavily tissue bound (per AZ label). The observed mean serum elimination half-life of AZ closely reflects the tissue depletion half-life of 2-3 days.[120, 121] The observed exposures of AZ and CQ in patients with malaria are consistent with that from healthy subjects.

Available AZ PK data suggests that no dosage adjustment is necessary in pregnant women. Pharmacokinetics of AZ following a single oral dose at 1000 mg was evaluated in 20 full term gravid pregnant women in the USA by Ramsey *et al*.[54] In this study, peak maternal

090177e1811dafca\Approved\Approved On: 22-Feb-2010 14:41

serum AZ concentrations occurred within six hours following oral administration. AZ levels in myometrial, adipose, and placental tissues were much higher than serum concentrations. Although only 2.6% of a maternal dose perfuses the placenta,[55] placental AZ concentrations were maintained 6-15 times of the corresponding serum AZ concentrations during the first 72 hours post-dose (Table 1 below).[54]

Table 1. Azithromycin Pharmacokinetic Data

Specimen	Time After Azithromycin Administration (ng/mL)				
	6 h (n=2)	12 h (n=7)	24 h (n=5)	72 h (n=5)	168 h (n=1)
Maternal serum	311 ±170	144 ±79	63 ±37	60 ±31	<10.4
Maternal myometrium	1382 ±549	1792 ±1107	1299 ±466	961 ±419	36 ±0
Maternal adipose tissue	1041 ±706	494 ±204	599 ±330	551 ±129	41 ±0
Placenta	2130 ±340	1196 ±224	936 ±223	915 ±628	40 ±0

Data are given as mean ± SD.

In a PK modeling analysis, AZ concentrations were predicted for pregnant women based on the known AZ exposures in term gravid pregnant women reported by Ramsey *et al*[54] and in healthy subjects described by Liu *et al*.[56] The predicted serum AZ concentrations in pregnant women were overlaid with the mean serum concentrations (with 95%CI) in healthy subjects (Figure 1), producing considerable overlap between the range of expected concentrations in both groups. Thus AZ exposures in pregnant women should be similar to healthy subjects.

Figure 1. Predicted Azithromycin Serum Concentrations in Healthy Subjects and Pregnant women

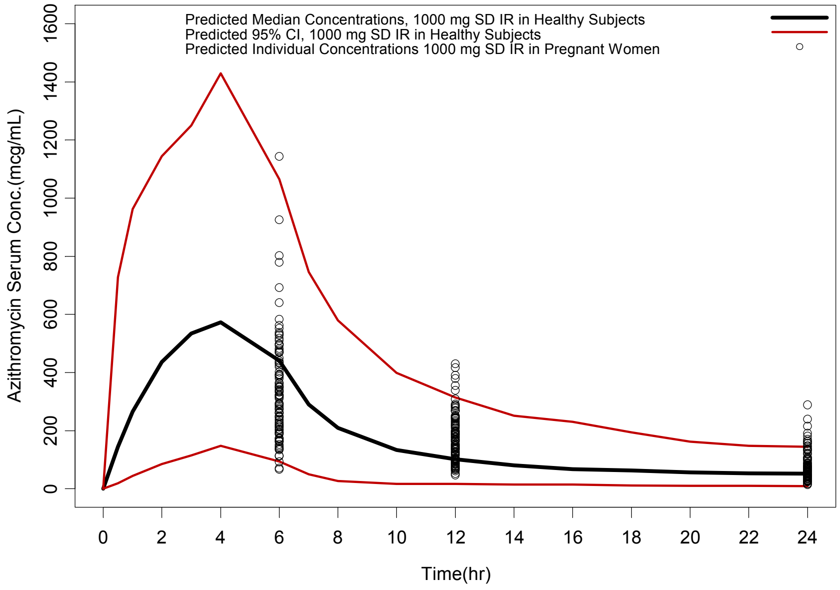

090177e1811dafca\Approved\Approved On: 22-Feb-2010 14:41

090177e1811dafca\Approved\Approved On: 22-Feb-2010 14:41

1.3.2.2. Clinical Efficacy of Azithromycin Monotherapy in Malaria

Daily regimens of 250 mg of AZ with a loading dose of 500 or 750 mg have shown an impressive chemoprophylactic effect against *P. vivax*. AZ had a 99% protective effect (95% CI, 93% to 100%) among semi-immune subjects in Indonesia over a 20-week period.[57] A similar protective efficacy, 98% (95% CI, 88% to 100%), was seen in Thailand[58] in a semi-immune population. By comparison, the chemoprophylactic effect of AZ against *P. falciparum* has shown promise, but has been less impressive. Among semi-immune populations, an equivalent or higher loading dose with the same daily regimen resulted in protective efficacies of 83% (95% CI, 69% to 91%) in Kenya,[59] 71% (95% CI, -14% to 94%) in Thailand,[58] and 72% (95% CI, 50% to 84%) in Indonesia.[57]

Human challenge studies with one *P. falciparum* strain revealed only partial causal prophylaxis but 100% suppressive prophylaxis.[60, 61] Although this high level of protection has not been replicated in the field where multiple infections may be expected, these data suggested that AZ has the potential to be an effective, well-tolerated clinical prophylactic agent for *P. falciparum* malaria.

In vivo treatment efficacy of AZ was measured indirectly as part of a clinical trial of trachoma control in The Gambia. A retrospective analysis of children in The Gambia receiving oral AZ versus tetracycline eye ointment for trachoma revealed that symptomatic parasitemia and spleen indices were significantly reduced in those who received AZ. These data were encouraging and prompted prospective studies evaluating the use of AZ in the treatment of uncomplicated *falciparum* malaria.[28] In a phase 2 treatment trial in India (066-191), 1000 mg of AZ were given to patients with smears positive for *P. falciparum* or *P. vivax* once a day for three days. The analysis indicated suboptimal efficacy (33% at Day 28) with AZ monotherapy.[62]

1.3.2.3. Clinical Effects of Azithromycin against Sexually Transmitted Infections

Sexually transmitted infections (STI) adversely affect pregnancy and contribute to pre-term delivery, LBW, intrauterine growth-retardation, spontaneous abortion, stillbirth, newborn morbidity and mortality.[63] The prevalence estimates of symptomatic STIs at antenatal clinics in sub-Saharan Africa range between 2.5 and 17% for *T. pallidum*, 1.7 and 7% for *Neisseria gonorrhoeae*, 5.3 to 20.8% for *C. trachomatis* and 7.3% to 62% for chancroid.[4] In addition, a shortcoming to STIs is the asymptomatic infection, which remains undiagnosed and untreated. If untreated in pregnancy, one-third of women will develop congenital syphilis, carrying major risk for the fetus.

AZ has significant activity against selected STIs (eg, *Chlamydia trachomatis*, *Neisseria gonorrhoeae, Treponema pallidum*). In a randomized clinical trial involving 4,033 pregnancies in Uganda,[64] the presumptive treatment of STIs in pregnancy improved maternal health and birth outcomes. Vaginal infections were significantly lower in women who received a one-time dose of azithromycin (1,000 mg), metronidazole (2,000 mg) and cefixime (400 mg) compared to women who received iron/folate and low-dose multivitamins. In the treatment group, the relative risk (RR) of T. vaginalis was 0.28 (95% CI, 0.18–0.49), the RR of bacterial vaginosis was 0.78 (95% CI, 0.69–0.87), and the RR of infant ophthalmia was 0.37 (95% CI, 0.20– 0.70).

Single oral doses of 1000 mg and 2000 mg of azithromycin are recommended for chlamydial and gonococcal infections.[65, 66] In two separate studies conducted in Tanzania,[67] and in United States & Madagascar,[68] a single 2000 mg dose of azithromycin was comparable to the standard dosing regimen of benzathine penicillin in the treatment of early syphilis. Hence, activity of AZ against these infections should provide additional benefit when AZ and CQ combination is used for IPTp in pregnant women.

1.3.2.4. Clinical Safety and Tolerability of Azithromycin

Doses of AZ between 500 mg and 2000 mg have been used in all trimesters of human pregnancy for the treatment of upper and lower respiratory tract infections, skin diseases, *C. trachomatis*, mycoplasma and group B streptococci infections among women allergic to other antibiotics,[4] No dosage adjustments have been recommended for use of AZ in management of STIs in pregnant women.[66]

Adults treated with a 1000 mg oral dose of AZ report mild to moderate side-effects including diarrhea or loose stools (7%), nausea (5%), vomiting (2%), and vaginitis (2%); up to 1% of adults experience dizziness, headache, vertigo, and somnolence.[69]

1.3.2.4.1. Clinical Safety of Azithromycin in Pregnancy

Meta-analysis of eight randomized clinical trials among pregnant women with *C. trachomatis* infection found that AZ was associated with fewer gastrointestinal adverse events than erythromycin (OR =0.11, 95% CI 0.07–0.18) and fewer total adverse events (OR =0.11, 95% CI 0.07–0.18).[70]

Bar-Oz and colleagues conducted a prospective multi-center study of pregnancy outcomes between pregnant women exposed to one of the new macrolides (clarithromycin, roxithromycin and AZ) during the first trimester of pregnancy (n=161) and two comparison groups exposed to either other antibiotics or other non-teratogens (n=953). The rate of major malformations in the macrolide exposed group was 4.1% compared to 2.1% in the other antibiotic exposed group and 3.0% in the other non-teratogens group. The calculated odds ratio (OR) comparing macrolides to other non-teratogens was 1.41 (95 % confidence interval: 0.47-4.23). However, among the malformations in the macrolide group (4/97) only one case involved AZ (1/27) and the remainder involved roxithromcyin (3/31). Based on these data, AZ did not appear to confer a higher risk of malformations compared to other antibiotics and other non-teratogens.[71]

Cooper and colleagues conducted a retrospective cohort study of 30,049 infants from Tennessee Medicaid who had been exposed to AZ and other antibiotics in utero compared to infants with no exposure to antibiotics (24521) in utero. Overall, 2.95% of the cohort had a confirmed major congenital malformation ranging from 2.5% to 3.0% according to antibiotic groups (doxycycline 2.5%, erythromycin 2.6%, ciprofloxacin 2.8%, multiple 2.8%, AZ 2.9%, amoxicillin 2.9%, none 3.0%). Compared to infants with no exposure to antibiotics, there was no statistically significant increased risk of overall congenital malformations in the group exposed to AZ over the entire pregnancy (relative risk (RR) 1.00, 95% confidence interval 0.71, 1.42).[72]

090177e1811dafca\Approved\Approved On: 22-Feb-2010 14:41

Kalliani and colleagues conducted a randomized controlled pilot trial of SP alone, SP plus AZ and SP plus artesunate in the treatment of malaria in 141 pregnant women.[73] No significant differences in birth outcomes were noted between the three groups. Congenital anomalies were not examined in this study.

1.3.2.4.2. Potential Risk of Macrolide Resistance to *S. pneumoniae* with Azithromycin

One of the potential concerns with the use of azithromycin for preventive treatment at a community level is emergence of macrolide and resistant pneumococci. Antibiotic resistance may emerge in a population of pneumococci because treatment eradicates susceptible organisms, favoring the survival of resistant strains. Alternatively, exposure of organisms to antibiotics may allow organisms which have developed point mutations in critical genes to survive.[74]

Mass trachoma prophylactic treatment campaigns have shown that macrolide-resistant pneumococcal strains were induced in the nasopharynx[75, 118] and conjunctiva[119] after the one-time administration of 1000 mg of AZ. Selection was, however, temporary. In Australia, 98.7% (78 of 79) of nasopharyngeal pneumococcal isolates collected at baseline were sensitive to AZ, decreasing to 84.2% (32 of 38) two to three weeks post-treatment, and then to 73% (27 of 37) when measured two months later. Six months post-treatment, 94.9% of isolates were sensitive to AZ.[75]

In a retrospective study conducted in Nepal, nasopharyngeal S pneumoniae isolates were collected for susceptibility to AZ one year after administering a single dose of AZ to treat trachoma in a village in Nepal. Among all S pneumoniae isolates (50 of 50), no resistance to AZ was detected.[76] In a large community trial conducted among children in Rombo District, northern Tanzania[1] suggested very high macrolide sensitivity of pneumococci in samples collected at pre-treatment, two months and six months post-treatment after community-wide administration of AZ. A total of 1315 samples were obtained at baseline, 1,225 at 2 months and 1402 at 6 months. The prevalences of S. pneumoniae carriage at these time points were 11% (141 of 1315), 12% (149 of 1225), and 7% (92 of 1,402), respectively. The most striking feature of the data is the almost complete absence of macrolide resistance. Only one erythromycin-resistant isolate was identified among nearly 4000 pneumococcal swabs collected and at six months post treatment.[77] PCR analysis of this resistant isolate indicated the bacterial mutation due to an efflux pump. These data presented in this study suggest that the impact of community-wide application of AZ is unlikely to increase the prevalence of macrolide resistance in communities where this type of resistance is rare before administration and where background use of azithromycin is low.

The planned IPTp study will investigate the sensitivity of *Streptococcus pneumoniae* against macrolides (erythromycin and azithromycin), and penicillin over time in a sub-group of subjects at (1) baseline, (2) Day 28-42 post delivery, (3) and about six-months following last IPTp course.

090177e1811dafca\Approved\Approved On: 22-Feb-2010 14:41

1.4. Chloroquine

1.4.1. Non-Clinical Summary of Chloroquine

1.4.1.1. Pharmacology and Microbiology of Chloroquine

CQ has been the first-line treatment of malaria in much of the world for most of the past 60 years. Due to widespread resistance, CQ is no longer recommended for treatment of falciparum malaria. Resistance to CQ is associated with polymorphisms in the *P. falciparum* food vacuole transporter protein (*pfcrt*) located on chromosome 7.[78] All *pfcrt* alleles from CQ-resistant strains, regardless of geographic origin, encode a conserved K76T amino acid substitution. The effect of *pfcrt* on CQ pharmacokinetics remains disputed. Some researchers have theorized that *pfcrt* enables protonated CQ to escape the food vacuole while others argue *pfcrt* binds directly to CQ, thereby inhibiting its ability to alter food vacuole pH.[79]

1.4.1.2. Non-clinical Safety of Chloroquine

Given the extensive clinical experience demonstrating the safety of CQ over the last 60 years, the available non-clinical information for CQ is limited and most studies are relatively old. No information was found in the literature concerning CQ administration to neonatal animals. The available non-clinical data for CQ suggest adverse events at doses 16-40 fold higher than those proposed for this malaria program.

1.4.1.2.1. Preclinical Safety of Chloroquine on Reproductive Systems

For CQ fertility/reproductive preclinical studies, limited information is available in the published literature. CQ can produce adverse embryonic effects/fetal abnormalities in experimental animal pregnancies, but at doses of CQ that are substantially higher than doses used for malaria prophylaxis or treatment.[80] Udalova *et al*[81] using a single dose of CQ 1000 mg/kg administered to pregnant rats on Day 9 of gestation reported an increase in embryonic deaths and fetal ocular malformations. Walker and Warner[82] reported that a single dose of CQ 400 mg/kg administered orally to rats between Days 8-9 of gestation produced mainly ocular malformations. Sharma and Rawat[83] orally administered CQ 700 mg/kg to rats on 15 days of gestation. A significant reduction in maternal body weight and placental weights were observed. Fetal growth retardation was also noted along with an increase in skeletal abnormalities. The authors concluded that the effects of CQ on the developing rat fetus are similar to that of ethanol. The above doses of CQ in animals are from 16- to 40-fold greater than the proposed dose of CQ 30 mg/kg body weight to be evaluated in the study.

1.4.2. Clinical Summary of Chloroquine

1.4.2.1. Clinical Pharmacology of Chloroquine

The pharmacokinetics of CQ were recently evaluated in healthy subjects in studies A0661118 and A0661186. Following oral administration, CQ is rapidly and almost completely absorbed from the gastrointestinal tract with mean peak plasma concentration

090177e1811dafca\Approved\Approved On: 22-Feb-2010 14:41

~ 5-7 hours. The mean plasma half-life of CQ is ~200 hours. CQ undergoes appreciable degradation in the body and the main primary active metabolite, desethyl-CQ, has a mean elimination half-life around 240 hours (10 days). Available CQ PK data suggests that there is no significant difference in the pharmacokinetics of CQ between pregnant and non-pregnant women and no dosage adjustment is required for CQ in pregnant women. Lee and colleagues[84] compared the pharmacokinetics of CQ and desethyl-CQ following administration of a standard 3 day 25 mg/kg dosing regimen of CQ in 12 pregnant and 13 non-pregnant patients with acute *P. vivax* malaria on the northwestern border of Thailand.[84] While the total area under the blood concentration-time curve ($AUC_{0-\infty}$) of CQ tended to decrease with the gestational age, the overall pharmacokinetics of CQ was not significantly different between pregnant and non-pregnant women. The AUC values for desethyl- CQ were not significantly affected by pregnancy either. The authors concluded that no dosage adjustment is required in pregnant women.[84]

1.4.2.2. Clinical Efficacy of Chloroquine Monotherapy in Malaria

Due to prevailing drug resistance in East Africa, CQ monotherapy is not used for chemoprophylaxis or treatment. It continues to offer some protective effect for pregnant women, however, in West Africa. A recent observational study in Benin examined the effect of self-administered CQ among pregnant women (N=1090), comparing self-reported dosing over pregnancy with birth weights. An estimated 49.9% of women reported taking weekly CQ in the first trimester, increasing to 92% of women in the second trimester and 97.5% in the final trimester. Subjects with self-reported chemoprophylactic use for seven or more months were four times more likely to give birth to child of normal birth weight (>2500 grams) than women who used chemoprophylaxis for less than four months (adjusted OR =3.96; 95% CI =1.9 to 8.28; p=<0.001).[85]

Although CQ is no longer recommended for treatment of *P. falciparum* due to high levels of resistance, particularly in east Africa, the return of parasite sensitivity to CQ has been documented in Malawi. In 1993, CQ *in vivo* failure rates in Malawi were as high as 58%.[86] Five years later, after changing to SP as first-line treatment, CQ inhibited in vitro blood schizont development in 96.5% (28 of 29) of isolates in Malawi,[87] indicating that *pfcrt* was no longer under selection pressure. In 2001, field sampling failed to find parasites carrying the *pfcrt* mutation associated with resistance[88] and a clinical trial using CQ monotherapy was 100% efficacious (63 of 63) among asymptomatic semi-immune adults who received 600 mg on Day 0 and Day 1, and 300 mg on Day 2.[89] Most recently, a study in 2005 showed CQ to clear parasite infection in 98.8% (79 of 80) of Malawi children with uncomplicated *P. falciparum* malaria.[90] The reemergence of high *in vitro* sensitivity to CQ in Malawi, within just five years, suggests the *pfcrt* resistance mutation involves considerable fitness cost to *P. falciparum*.[91-93] Kenya recently reported similar micro-evolutionary changes in P. *falciparum* since suspending CQ use in 1999, but at a slower rate than Malawi. Many reasons may contribute to delayed return of sensitivity, not the least of which is that CQ continued to be widely available for four years after the national treatment policy was changed, and the fact that amodiaquine, an analogue of CQ, was the second-line treatment during the CQ era and continues to be used to this day as the second-line therapy.[94]

090177e1811dafca\Approved\Approved On: 22-Feb-2010 14:41

Evidence of CQ efficacy was reported, as well, in a recent four-arm clinical trial conducted in Ghana among pregnant women with asexual *P falciparum* stage parasitemia. Women randomized to a CQ treatment group (N=225) received 600 mg for 2 days and 300 mg on the third day. The uncorrected day-28 treatment failure rate was 30% (62 of 208). Polymerase chain reaction (PCR) analysis confirmed that 14% (30 of 208) were treatment failures while 6% (11 of 208) were re-infections. PCR was unable to distinguish cases of recrudescence from new infection in the remaining 10% (21 of 208).[23] In a Phase 2 treatment trial in India (066-191), CQ were given to patients with smears positive for *P. falciparum* at dose of CQ base 600 mg for first two days and 300 mg on the third day. The analysis indicated suboptimal efficacy (27% at Day 28) with CQ monotherapy.[62]

1.4.2.3. Other Potential Clinical Benefits of Chloroquine

CQ may offer protection against HIV transmission. Cord blood containing high levels of CQ has been associated with a reduced risk of mother-to-child transmission (MTCT) of HIV.[95] Viral shedding in breast milk has been lowered among HIV-positive women who received three days of 600 mg CQ as an anti-malarial chemoprophylactic.[96] It is unknown whether this reduction in viral load is sufficient to prevent HIV transmission among mothers who choose to breastfeed. CQ, having anti-inflammatory properties,[97] has been shown to reduce systolic pressure and increase blood flow[98] and may offer potential protection against pre-eclampsia.

1.4.2.4. Clinical Safety and Tolerability of Chloroquine

CQ is safe and generally well tolerated in treatment doses. Due to its rapid absorption, CQ has a narrow therapeutic index, increasing the potential for toxic overdose. Hypotension and cardiac failure can be prompted by a single oral dose of 3500 mg.[99] Despite toxicity at high doses, the most commonly reported side-effect in African populations is pruritus which is mostly mild in intensity and peaks 24 hours after intake.[100]

1.4.2.4.1. Clinical Safety of Chloroquine in Pregnancy

CQ has been widely used since the 1940s for the treatment of malaria and is included in WHO and the US Centers for Disease Control and Prevention (CDC) guidelines for the treatment women in all trimesters of pregnancy infected with susceptible strains of the parasite. It has been demonstrated safe crossing the placenta without teratogenic effect.[101] A review of the medical literature regarding the use of CQ in pregnant women did not identify any adverse outcomes when pregnant women are treated in accordance with dosing regimens suggested in the WHO and the CDC guidelines for the treatment of malaria.

Three publications were identified that describe clinical trials in pregnant women where CQ was administered, none reported any safety-related information in either women or their offspring to be of use to this review.[102-104]

Law and colleagues performed a study of cord/maternal drug distribution in 19 mothers who had been prescribed IPTp with CQ (750 mg daily) for 3 consecutive days for IPTp, and assessed breast milk transfer following another course of CQ administered in the early

090177e1811dafca\Approved\Approved On: 22-Feb-2010 14:41

postnatal period (days 1–3 after delivery).[105] Absolute infant dose via milk (mg/kg/day) was calculated as the product of the milk Cavg and an average infant milk intake of 0.15 l/kg/day. Relative infant dose was calculated as absolute infant dose/days of exposure (mg/kg)/maternal dose (mg/kg) and expressed as a percentage. The median absolute and relative infant doses were 34 mg/kg/day and 2.3% for CQ and 15 mg/kg/day and 1.5% for desethyl-CQ, respectively. The absolute dose is well below recommended pediatric and neonatal treatment doses and the combined relative infant dose (CQ plus desethyl-CQ =3.2%) is also significantly lower than the recommended 10% safety cut-off. The authors believe that this, in addition to the absence of observed adverse effects, is supportive of CQ's compatibility for use at recommended doses during breast-feeding.

1.5. Azithromycin-Chloroquine Combination (AZCQ)

1.5.1. Non-Clinical Summary Information

1.5.1.1. Non-Clinical Efficacy of the Azithromycin and Chloroquine Combination against *P. falciparum*

In an *in vitro* study by Ohrt *et al*,[106] AZ and CQ combination demonstrated activity ranging from additive to synergistic against eight CQ resistant P. *falciparum* isolates and exhibited an additive response against two CQ sensitive strains in vitro. Addictive effects were also observed in studies conducted by Gingras[47] and Sidhu.[37] Fidock *et al* at Columbia University recently demonstrated potent in vitro synergy of AZ and CQ against CQ resistant P. *falciparum* strains from Bamako, Mali investigational site for Pfizer's adult treatment study A0661155 with the fractional inhibitory IC_{90} values of 0.3 to 0.4. (Personal communication, ASTMH, New Orleans, December 9, 2008).

One of the considerations involving the use of AZ in the treatment of malaria has been the slower rate of resolution of parasitemia, possibly a consequence of its mechanism of action. If combined with a fast-acting parasiticidal agent, the therapeutic utility of AZ may be optimized. The addition of a more rapidly cidal agent to AZ had been recommended as a way to optimize its application in a clinical setting. CQ has been demonstrated to rapidly reduce the circulating burden of circulating malaria organisms. A combination of CQ and AZ may therefore be rapidly parasiticidal while reducing the likelihood of breakthrough resistance.

1.5.1.2. Non-Clinical Safety of Azithromycin and Chloroquine Combination

Since CQ and macrolides (except AZ) are known to prolong QT interval of electrocardiogram (ECG) measurements, the combination of AZ and CQ was evaluated in a non-clinical model to assess the arrythmogenicity potential of the combination. Alternans (action potential duration alternations) is a measure of cardiac instability in humans and animals associated with the onset of ventricular fibrillation. Arrythmogenicity potential of CQ, AZ and combination of CQ and AZ were assessed in anesthetized guinea pig alternans model following administration AZ and CQ alone and following combination treatment at clinically relevant concentrations proposed for the treatment/IPT of malaria. CQ alone, but not AZ, caused a profound increase in action potential duration. None of the drugs or their

090177e1811dafca\Approved\Approved On: 22-Feb-2010 14:41

090177e1811dafca\Approved\Approved On: 22-Feb-2010 14:41

combination had any significant effects on alternans at the adjusted free drug concentrations studied. These concentrations represented 1- to 2-fold the Cmax levels with either drug alone or 10- to 15-fold the maximum free drug concentrations anticipated following dosing with CQ and AZ in clinical studies. Despite the ability of CQ to inhibit hERG current and prolong QT potential, it appears to lack proarrhythmic liability at concentrations at or exceeding clinically relevant levels alone or when used in combination with AZ.

1.5.2. Clinical Summary Information for Azithromycin and Chloroquine Combination

1.5.2.1. Clinical Pharmacology of Azithromycin and Chloroquine Combination

The pharmacokinetics of AZ and CQ was characterized in healthy subjects following co-administration of AZ and CQ tablets in healthy volunteers in study A0661118. AZ had no clinically relevant effect on CQ pharmacokinetics and CQ had no clinically relevant effect on AZ pharmacokinetics either. In a clinical pharmacology study A0661186, the systemic exposures (Cmax and AUClast) to AZ, CQ and desethyl CQ following fixed dose combination of AZCQ tablets were comparable to those achieved following administration of commercial standalone tablets of AZ (Zithromax) and CQ (Aralen) evaluated in earlier adult treatment studies, A0661134 and A0661155. (Table 2)

Table 2. PK Parameters from A0661186 Study Formulation

	**Cmax[a] (ng/ml)**	**AUClast[a] (ng.hr/ml)**
**Chloroquine**		
Azithromycin 500 mg and Chloroquine base 300 mg individual tablets	108 (34)	3281 (30)
Azithromycin and Chloroquine base 250/150 mg X 2 tablets	95.8 (36)	3253 (35)
**Azithromycin**		
Azithromycin 500 mg and Chloroquine base 300 mg individual tablets	437 (38)	3877 (32)
Azithromycin and Chloroquine base 250/150 mg X 2 tablets	496 (40)	3910 (30)

a. Geometric mean (CV%)

In a recently published study, Salman and colleagues evaluated the pharmacokinetic properties of AZ for its use in combination with CQ for IPTp in 31 pregnant and 29 age-matched non-pregnant Papua New Guinean participants. All study participant received two 2000 mg doses of AZ that were given 24 h apart to pregnant and age-matched non-pregnant participants in combination with 450 mg CQ base daily for three days. The results demonstrated that in pregnant and non-pregnant participants, AZ exposures ($AUC_{0-\infty}$:28.7 vs 31.8 mg·h/L respectively) and the estimated terminal elimination half-lives (78 vs 77 hours, respectively) were similar.[107] The characterized pharmacokinetics of AZ in this study are also consistent with the results of pervious studies.[107] This study did not evaluate CQ pharmacokinetics.

1.5.2.2. Clinical Efficacy of Azithromycin-Chloroquine Combination against *P. falciparum*

The clinical efficacy of AZ and CQ co-administration was investigated in several Phase 2/3 clinical studies. The efficacy outcomes from these studies are summarized below.

- A two-stage treatment trial in India demonstrated *in vivo* synergy between AZ and CQ against P. *falciparum* infection.[62] In the first stage of the trial (066-191), neither CQ monotherapy (27% efficacy at Day 28) nor AZ monotherapy (33% efficacy at Day 28) demonstrated adequate efficacy. During the second stage (066-191B), the combination of AZ and CQ demonstrated a 97% efficacy at Day 28. The dosing regimen used in this study was 1000 mg AZ once daily and 600 mg CQ base once daily for 3 days.

- Subsequently, the combination of AZ and CQ was evaluated for acute uncomplicated *falciparum* malaria in three Phase 2/3 studies, A0661120, A0661126 and A0661154 in India and South America regions. A dose response for AZ (on mg/kg basis) was observed. While efficacy rates observed in India (A0661120) and South America (A0661126) were suboptimal (86.3% and 57.1% respectively) with 1000 mg of AZ dose, the efficacy rate with the regimen containgin higher AZ dose of 2000 mg in a subsequent study in A0661154 was 97.2% at Day 28 (96.4% at Colombia and 98.1% at India) with a higher AZ dose of 2000 mg. The treatment was well tolerated.[108]

- Two further Phase 2b/3 randomized comparative studies (A0661134 and A0661155) were conducted in sub-Saharan Africa in adults with the combination regimens of 1000 mg AZ and 600 mg CQ, once daily for 3-day treatment. Study 1134 was conducted in five countries in Africa including Ghana, Mali, Zambia, Kenya and Uganda. Study 1155 was conducted in Ghana, Mali, Zambia, Burkina Faso and Senegal. Both studies demonstrated efficacy, safety, and tolerability in the treatment of acute uncomplicated *falciparum* malaria. The Day 28 efficacy rates were 98%[109] and 100%[110] (PCR corrected), and 95% and 99% (PCR uncorrected) for study A0661134 and A0661155, respectively.

- A pediatric Phase 2/3 clinical trial (A0661157) of AZCQ is ongoing in Kenya, Mali, Ghana, Burkina Faos and Cote d'voire. This is a comparative study designed to evaluate a fixed dose AZCQ combination regimen of 30 mg/kg of AZ plus 10 mg/kg of CQ base once daily for 3 days vs. standard regimen of AL (artemether/lumefantrine) in treatment of symptomatic uncomplicated malaria. The study is being conducted in two sequential age-based cohorts. Enrollment in the first cohort older subjects between ≥5 years of age and ≤12 years of age is complete. Enrollment in the primary target Cohort 2, with younger subjects between ≥6 months of age to ≤59 months of age is ongoing. A blinded interim analysis (IA) was recently conducted at 50% completion of Cohort 2. The Based on review of IA data, the Independent Data Monitoring Committee (IDMC) for this program recommended continuing with the study.

090177e1811dafca\Approved\Approved On: 22-Feb-2010 14:41

1.5.2.3. Clinical Safety and Tolerability of Azithromycin and Chloroquine Combination

AZ and CQ are marketed compounds with well established safety profiles in adults and children. The combination of AZ and CQ at doses up to 2000 mg (~30 mg/kg) AZ plus CQ 600 mg base (~10 mg/kg) daily for three days appears well tolerated from the data derived from 066-191/b, A0661120, A0661118, A0661126, A0661134, A0661139, A0661154 and A0661155 studies. Phase 1 trial A0661139 demonstrated comparable tolerability between the combination of 1000 mg and CQ 600 mg (base) daily for three days and the combination of 1500 mg and CQ 600 mg base daily for three days. Most adverse events reported with AZ and CQ co-administration are mild in nature and are related to GI tract in origin. Pruritus is an adverse event commonly associated with CQ and is observed with the combination as well.

CQ is known to delay cardiac repolarization through inhibition of the rapidly activating delayed rectifier potassium current known as hERG and treatment with CQ has been shown to cause QT prolongation in patients. Macrolides (other than AZ) are also known to prolong QT interval on ECG measurements, however, this effect has not been observed with AZ. In the cardiovascular safety study A0661139, co-administration with AZ did not significantly affect the QTc effect associated with CQ use. Use of AZ or CQ has not been associated with torsades de pointes, a type of fatal arrhythmia often associated with drugs that cause QT prolongation (refer to the investigator brochure for details).

1.5.2.4. Clinical Safety of Azithromycin and Chloroquine Combination in Pregnancy

Both AZ and CQ have been widely used in all trimesters of pregnancy and are considered safe in pregnant women as individual agents. AZ has been used in all trimesters of pregnancy for STI and bacterial infections with dosage between 500 mg and 2000 mg.[66] CQ was recommended for prevention and treatment of malaria in all trimesters of pregnancy until recently with CQ resistance becoming widespread and the treatment courses with less than 2000 mg CQ base (total dose) produced similar rates of adverse events.[111]

A comprehensive review of the literature to identify publications describing the safety of CQ and AZ in pregnant women did not identify any additional safety issues beyond the information contained in the CQ and AZ product labels. In the doses used for malaria treatment and prophylaxis in pregnant women, an increased risk of adverse pregnancy outcomes has not been reported with CQ. With regard to AZ, many years of worldwide use, in addition to randomized trials and observational epidemiology studies have not identified any adverse pregnancy outcomes associated with AZ exposure during pregnancy.

To date there are no well-controlled clinical studies evaluating the effects of co-administration of AZ and CQ in pregnant women also containing safety information. In Pfizer trials where both drugs were used concomitantly for the treatment of uncomplicated malaria in adults, no additional safety concerns beyond those listed in the labels of the individual products were identified. No pharmacokinetic interaction is observed between the two drugs.

090177e1811dafca\Approved\Approved On: 22-Feb-2010 14:41

Please refer to the IB for additional information regarding the use of AZ and CQ combination in Malaria.

1.6. Rationale for AZCQ in IPTp

The benefits of current IPTp strategy are threatened by increasing resistance of *P. falciparum* to SP. Although review of SP efficacy data for pediatric treatment and IPTp suggests that SP may still offer some protection against malaria in pregnancy (MIP) in geographic areas where day 14 post-treatment failure rates for SP in children are as high as 39.,[112] This protection, however, is not uniform across populations. Primigravidae, who are particularly vulnerable to the effects of MIP, are protected the least by SP in areas where SP sensitivity is on the decline. In Ghana, where parasite sensitivity to SP remains higher than in east and southern Africa, uncorrected parasitological failure rates by Day 28 post-treatment were 36.4% (32 of 88) in children, 27.1% (29 of 107) in primigravidae, 6.1% (3 of 49) in secundigravidae, and 3.8% (2 of 52) in multigravidae.[113] Thus, SP is already compromised and an urgent need exists to identify alternative compounds for use in IPTp.

AZCQ offers several reasons for its evaluation as a potential replacement for SP in IPTp:

1) AZ and CQ combination is synergistic against CQ resistant strains of *P. falciparum* as demonstrated in vitro and in vivo;

2) Coadministration of AZ and CQ has demonstrated efficacy, safety and tolerability in two Phase 3 clinical studies (A0661134 and A0661155) in the treatment of symptomatic uncomplicated malaria in adults in sub-Saharan Africa;

3) AZ and CQ have been in market for several years and have extensive safety database in adults, children and pregnant women;

4) AZ and CQ have also been widely used in all trimesters of pregnancy and are considered safe in pregnant women as individual agents; and

5) Treatment and prevention of sexually transmitted infections including *T. pallidum* (syphilis), *N. gonorrhoeae* and *C. trachomatis* infections has been demonstrated to lower the incidence of adverse pregnancy outcomes by about 30%; hence, activity of AZ against these infections should provide additional benefit.

This study is a Phase 3 clinical trial aimed at demonstrating superiority of AZCQ over SP in countries in East and Southern sub-Saharan Africa, where SP is the current standard of care, and where SP resistance is a considerable issue. This will be the pivotal study for regulatory submissions to the EMA and to the national regulatory agencies in sub-Saharan African countries.

090177e1811dafca\Approved\Approved On: 22-Feb-2010 14:41

1.6.1. Dosing Regimen Selection

1.6.1.1. Sulfadoxine-Pyrimethamine (SP) as Comparator

Although SP resistance is increasing especially in East and Southern Africa, it remains the WHO recommended treatment for IPTp throughout the region.[15] SP will be administered in standard dosing regimen recommended for IPTp. Each subject will receive three IPTp treatment courses. The first course will be administered at the first antenatal visit in second trimester. The subsequent two courses will be administered at 4-8 weeks interval between courses in second/third trimester. Each treatment course will consist of a single oral dose of 1500 mg sulfadoxine and 75 mg pyrimethamine ie, 3 fixed dose tablets of SP (500 mg/25 mg strength). All treatment courses will be administered under supervision at the ANC clinic.

1.6.1.2. Azithromycin-Chloroquine (AZCQ)

AZCQ will be administered as three IPTp treatment course regimens during second and third trimester. The first treatment course will be administered at the first ANC visit in second trimester. The subsequent two courses will be administered at 4-8 weeks interval between courses in second/third trimester. Each treatment course will consist of 3-day of dosing: 1000 mg AZ/620 mg CQ, ie, 4 fixed dose tablets of AZCQ (250mg/150mg strength) administered once daily for 3 days. First dose will be administered under supervision at the ANC; subsequent two doses will be administered at home under supervision of a field worker.

Rationale for AZCQ Dosing Regimen: The dosing regimen of AZCQ has been selected for the IPTp program for the following reasons:

1. The above 3-day dosing regimen (1000 mg AZ/620 mg CQ, ie, 4 fixed dose tablets of AZCQ (250 mg/150 mg strength) administered once daily for 3 days) was evaluated and demonstrated efficacy in two adult treatment clinical trials (A0661134 and A0661155) in sub-Saharan Africa. Both PCR corrected and uncorrected parasitological efficacy rates were above 90%. For details on studies A0661134 and A0661155 (Refer to Section 1.5.2.2. and the IB).

2. In a clinical pharmacology study in healthy subjects (A0661186), the pharmacokinetics of the fixed dose AZCQ combination tablet appeared to be comparable to that of the individual tablets of AZ and CQ used in earlier adult treatment trials (Refer to Section 1.5.2.1. and the IB).

3. Although pregnant women experience unique physiological changes that can alter drug disposition during gestation, available data[93-95, 97] and PK modeling suggests that AZ and CQ PK are not significantly different between pregnant and non-pregnant women.

4. A total AZ dose of 3000 mg to be used for each IPTp treatment course is higher than the 2000 mg dose needed for treatment of STIs including *T. Pallidum*, *C. trachomatis*, and *N. gonorrohoea*.

090177e1811dafca\Approved\Approved On: 22-Feb-2010 14:41

A fixed dose combination tablet formulation AZCQ has been developed for the IPTp clinical program. Each tablet is labeled to contain 250 mg AZ and 155 mg CQ base. Please note that the content of CQ base in this tablet is equivalent to that contained in the commercial tablets of CQ (Aralen, 150 mg CQ base) used in the previous studies (A0661134 & A0661155). CQ is present in these tablets in the form of its phosphate salt. The 5 mg difference in strength is explained by the use of slightly different (more accurate) CQ base potency factors for in the CQ phosphate salt than what is used for the Aralen tablets. The combination tablet uses the actual potency factor (62.0%) vs Aralen that uses an approximate number of 60% for potency factor.

2. STUDY OBJECTIVES AND ENDPOINTS

2.1. Objectives

The primary objective is to establish superiority of AZCQ over SP in protective efficacy for IPTp as measured by the proportion of subjects with sub-optimal pregnancy outcome.

Key secondary objectives include comparison of IPTp regimens of AZCQ and SP in:

1. Proportion of subjects with LBW (<2500 g) live neonates;
2. Proportion of subjects with severe anemia (hemoglobin <8 g/dL);
3. Proportion of subjects with anemia (hemoglobin <11 g/dl);
4. Proportion of subjects with placental parasitemia;
5. Occurrence of sexually transmitted infections (STIs); and
6. Safety and tolerability of the two treatment regimens.

2.2. Endpoints

<u>Primary Efficacy Endpoint</u>

- Presence of subjects with a sub-optimal pregnancy outcome defined as any of the following: live-borne neonate (singleton) with low birth-weight (or LBW for short, defined as live birth weight <2,500g), premature births (<37 weeks), abortion (≤28 weeks), still birth (>28 weeks), lost to follow-up prior to termination of pregnancy or delivery, or missing birth weight of the neonates.

<u>Key Secondary Efficacy Endpoints</u>

1. Occurrence at birth of a LBW live neonates;
2. Occurrence of severe maternal anemia (Hb <8 g/dL) at 36-38 weeks of gestation;
3. Occurrence of anemia (Hb <11 g/dL) at 36-38 weeks of gestation;

090177e1811dafca\Approved\Approved On: 22-Feb-2010 14:41

4. Occurrence of placental parasitemia at delivery;

5. Occurrence of placental malaria as determined by histology;

6. Number of episodes of STIs per subject including *T. pallidum*, *N. gonorrhoeae*, *C. trachomatis*, during the study period following first dose (diagnosis based on clinical presentation any time from first IPTp dose to delivery and/or on laboratory test results between Weeks 36-38).

Other Secondary Efficacy Endpoints

1. Hemoglobin concentrations at 36-38 weeks of gestation;

2. Occurrence at birth of a neonate with congenital abnormalities;

3. Occurrence of a perinatal or neonatal deaths;

4. Birth weight of the live-borne neonate (singleton);

5. Number of episodes of symptomatic malaria per subject anytime from first IPTp dose administration to delivery;

6. Occurrence of a subject requiring additional treatment for malaria during the study period following the first dose (diagnosed based on clinical presentation and/or lab test results);

7. Occurrence of peripheral parasitemia at 36-38 weeks of gestation;

8. Occurrence of peripheral parasitemia at delivery;

9. Occurrence of cord blood parasitemia at delivery;

10. Occurrence of STIs including *T. pallidum*, *N. gonorrhoeae*, *C. trachomatis* during the study period following first dose (diagnosed based on clinical presentation prior to Week 36-38 and/or lab test results between Week 36-38);

11. Occurrence of a positive result for *C. trachomatis* infection at 36-38 weeks of gestation (diagnosed based on lab result);

12. Occurrence of a positive result for *N. gonorrhoeae* infection at 36-38 weeks of gestation (diagnosed based on lab result);

13. Occurrence of a positive result for *T. pallidum* test at 36-38 weeks of gestation (diagnosed based on lab result);

14. Occurrence of a *T. vaginalis* infection at 36-38 weeks of gestation (diagnosed based on lab result);

090177e1811dafca\Approved\Approved On: 22-Feb-2010 14:41

15. Occurrence of bacterial vaginosis at 36-38 weeks of gestation (diagnosed based on lab result);

16. Occurrence of ophthalmia neonatorum (diagnosed based on lab test results) in the neonate;

17. Occurrence of bacterial infections including pneumonia and other lower respiratory tract infections anytime from first IPTp dose administration to delivery;

18. Occurrence of pre-eclampsia from Week 20 to delivery;

19. Occurrence of nasopharyngeal swabs positive for macrolide resistant and penicillin resistant *Streptococcus pneumoniae* at baseline, at Day 28 (window Day28 - Day 42) post delivery, and at about 6 months following last IPTp course. This test will be done in about 600 subjects each from the AZCQ and SP arms from two or more sites.

Safety Endpoints

Safety and tolerability will be assessed by spontaneously reported adverse event reports, vital signs, physical examination, laboratory tests including hemoglobin and urine test for glucose and protein, and adverse pregnancy outcomes for mothers and by the general physical examination for the neonates through Day 28 (window Day 28 to Day 42) post delivery.

Outcome Research Endpoints

To assess health economic impact from the health system and provider perspective, as measured by in-hospital and out-of-hospital healthcare utilization for the newborn/infant and the mother consumed due to low birth weight, infections and health complications. The endpoints will include:

1. Number of times a newborn/infant is taken to a local health clinic/physician's office/outpatient hospital clinic including Emergency Room (without having to be admitted) during the first 28 days (window Day 28 - Day 42) of life to attend to a health complication and the primary reason for visit;

2. Number of times a newborn/infant is admitted to a hospital during the first 28 days (window Day 28 - Day 42) of life, including length of hospital stay;

3. Number of times a mother is taken to a local health clinic/physician's office/outpatient hospital clinic including Emergency Room (without having to be admitted) from the time of the administration of the first dose of IPTp through Visit 6 on Day 28 (window Day 28 - Day 42) post delivery for treatment (or follow up) of Anemia, Malaria, STD or other complications and primary reason for the visit;

4. Number of times a mother is admitted to a hospital from the time of the administration of the first dose of IPTp through Visit 6 on Day 28 (window Day 28 - Day 42) post delivery, including length of stay in hospital.

090177e1811dafca\Approved\Approved On: 22-Feb-2010 14:41

3. STUDY DESIGN

This is a Phase 3, open label, randomized, parallel group study that will compare the efficacy of IPTp regimens of AZCQ and SP in asymptomatic pregnant subjects enrolled during second trimester of pregnancy. Approximately half of the subjects will be primigravidae and secundigravidae pregnant women since they have a higher risk for suboptimal pregnancy outcomes due to malaria. They will be randomized to receive either AZCQ or SP after informed consent is obtained (and assent if applicable) and inclusion/exclusion criteria are determined to have been met. Each subject will receive three IPTp courses of AZCQ or SP during ANC visits at 4 - 8 week intervals. The first treatment course will be administered during the second trimester (14-26 weeks of gestation as confirmed by ultrasound). The last treatment course should be given to subjects prior to or during 36 weeks of gestation. Subjects should be followed up at delivery or within 2 days of subject reporting home delivery (report within 24 hours of delivery), and on Day 28 (window: Day 28 to Day 42) post-delivery. Long lasting insecticidal-treated bednets (LLINs) will be given to all subjects on Day 0 of the study with the instructions to use them; the installation of LLINs will be verified during the first home visits of Treatment Course 1 by fieldworker(s).

4. SUBJECT SELECTION

This study can fulfill its objectives only if appropriate subjects are enrolled. The following eligibility criteria are designed to select subjects for whom protocol treatment is considered appropriate. All relevant medical and non-medical conditions should be taken into consideration when deciding whether this protocol is suitable for a particular subject.

Subjects who fail to meet the selection criteria for the study will be referred to the appropriate physician for receiving standard ANC care including IPTp treatment as per local/national guidelines.

4.1. Inclusion Criteria

Subject eligibility should be reviewed and documented by an appropriately qualified member of the investigator’s study team before that subject is included in the study. Subjects must meet all of the following inclusion criteria on Day 0 at Visit 1 to be eligible for enrollment into the study:

1. Pregnant women (all gravidae) with ≥14 and ≤26 weeks of gestational age (defined by ultrasound).

2. Evidence of a personally signed and dated informed consent document indicating that the subject (or a legally acceptable representative if a subject is <18 years of age) has been informed of all pertinent aspects of the study and that all questions by the subject have been sufficiently answered. Assent will be obtained from subjects <18 years of age.

3. Subjects who are willing and able to comply with scheduled visits, treatment plan, laboratory tests, and other study procedures.

090177e1811dafca\Approved\Approved On: 22-Feb-2010 14:41

4. Subjects who agree to be supervised for treatment administration, and are available for all follow up visits as per protocol, including follow ups at delivery and on 28 days post delivery.

4.2. Exclusion Criteria

Subjects presenting with any of the following will not be randomized in the study:

1. Age <16 years old or >35 years old.
2. Multiple gestations (more than one fetus) as per the ultrasound results at screening.
3. Clinical symptoms of malaria.
4. Hemoglobin < 8 g/dL (measured at baseline).
5. Any condition requiring hospitalization or evidence of severe concomitant infection at time of presentation.
6. Use of antimalarial drugs in previous 4 weeks.
7. History of convulsions, hypertension, diabetes or any other chronic illness that may adversely affect fetal growth and viability.
8. Inability to tolerate oral treatment in tablet form.
9. Known allergy to the study drugs (AZ, CQ, and SP) or to any macrolides or sulphonamides.
10. Present history of smoking or alcohol or drug abuse since first becoming aware of current pregnancy.
11. Participation in other studies within 30 days before the current study begins and/or during study participation.
12. Inability to comprehend and/or unwillingness to follow the study protocol.
13. Concurrent participation in another investigational study.
14. Previously randomized in this study.
15. Requirement to use medication during the study that might interfere with the evaluation of the study drug (eg, trimethoprim-sulfamethoxazole use in subjects positive for HIV infection) or is contra-indicated during pregnancy per package inserts.

090177e1811dafca\Approved\Approved On: 22-Feb-2010 14:41

16. Severe acute or chronic medical or psychiatric condition or laboratory abnormality that may increase the risk associated with study participation or investigational product administration or may interfere with the interpretation of study results and, in the judgment of the investigator, would make the subject inappropriate for entry into this study. Examples would include but not limited to:

    a. Symptomatic HIV infection, including physical findings that suggest immunocompromised status (eg, oral candidiasis);

    b. Neurological conditions which may predispose to complications during pregnancy, including seizure disorders;

    c. Severe psychosis or major disorder that may interfere with the conduct of the study or adherence to study medication;

    d. Known, clinically significant pre-existing renal or hepatic disease.

17. Evidence of current obstetric complications that may adversely impact the pregnancy and/or fetal outcomes, including presence of congenital anomalies, placenta previa or abruption.

18. Known severe sickle cell (SS) disease or sickle-hemoglobin C (SC) anemia.

19. Known family history of prolonged QT Syndrome, serious ventricular arrhythmia, or sudden cardiac death.

4.3. Randomization Criteria

A computer-generated randomization list will be provided by the sponsor to the investigators. Randomization will be stratified according to gravidae (primigravidae & secundigravidae vs. other gravidae). Among all gravida, approximately 50% subjects will be stratified with primi gravidae & secundigravidae. The investigator will assign subject identification numbers sequentially as they are determined to be eligible for treatment (PID numbers). This unique PID number will be retained throughout the study and must appear on all case report form (CRF) pages, source documents and lab data. The eligible subjects who have met all inclusion/exclusion criteria will receive study medication assigned to the next available randomization number at the Day 0 during the first study visit. The randomization number will also be recorded on the proper CRF page.

4.4. Life Style Guidelines

Subjects will be encouraged to stay within the area during the entire pregnancy period and deliver in the hospital setting. Subjects will be encouraged to use LLINs. Site staff will discuss with subjects about the plan of hospital delivery. In case of home delivery, the subject will be asked to contact the study sites and report delivery within 24 hours of delivery. Fieldworker should visit the subject at home within 2 days of subject reporting delivery, and collect and/or transcribe data required in the study. If the site has not been notified of the delivery within 3 days post subject's due date, the study site will follow up with the subject about the delivery.

090177e1811dafca\Approved\Approved On: 22-Feb-2010 14:41

5. STUDY TREATMENTS

The Study treatment is a fixed dose tablet formulation of AZ and CQ (AZCQ) containing 250 mg AZ and 155 mg CQ base. Comparator drug is SP and will be supplied as Fansidar® (Roche) tablet (500 mg sulfadoxine/25 mg pyrimethamine). Subjects will be randomized in 1:1 ratio to receive either AZCQ or SP IPTp regimens. Both regimens will consist of three treatment courses. The treatment courses of AZCQ and SP will consist of the following:

1. **AZCQ treatment course**: 1000 mg AZ and 620 mg of CQ base (4 combination tablets of AZCQ with individual strength of 250 mg/155 mg), by mouth (PO) once daily for 3 days (Days 0, 1, 2);

OR

2. **SP treatment course:** 1500 mg sulfadoxine and 75 mg pyrimethamine (3 fixed tablets of SP strength at 500 mg/25 mg), PO single dose once daily on Day 0.

Each subject will receive three IPTp treatment courses of AZCQ or SP during ANC visits at 4 - 8 week intervals. The first treatment course will be administered during the second trimester (14-26 weeks of gestation as confirmed by ultrasound). The last treatment course should be given to subjects prior to or during 36 weeks of gestation.

The first dose of each AZCQ treatment course will be administered under supervision during the ANC visits. Subsequent two doses of AZCQ will be administered at home under supervision by the fieldworker(s).

Each dose of SP treatment will be administered under supervision during the ANC visits.

Please note that the terms course and dose are often used synonymously in IPTp studies using SP because only one directly observed dose is required per treatment course. Within this protocol, dose represents medications taken on a given day; course means all medications required for a complete treatment at one visit.

Contraindications:

The Investigator and study staff must be thoroughly aware of all contraindications and other information contained in the Package Insert for SP (Fansidar), AZ (Zithromax), CQ (Arelen), and IB for the study medication AZCQ.

5.1. Allocation to Treatment

The investigator's knowledge of the treatment must not influence the decision to enroll a particular subject or affect the order in which subjects are enrolled. Refer to Section 4.3 (Randomization Criteria) for details.

090177e1811dafca\Approved\Approved On: 22-Feb-2010 14:41

5.2. Breaking the Blind

The study is open label. However, the microbiologists (microscopists) will be blinded to the study treatments of the subject and the reading of other microscopists. The lab scientists undertaking the laboratory investigations (ie, molecular assays, biochemical assays, histological and microbiological investigations) will be unaware of the study group from which the sample was obtained.

5.3. Drug Supplies

All trial medication will be supplied by Pfizer.

AZ and CQ will be administered as a combination tablet containing 250 mg AZ and 155 mg CQ base. This is a research tablet, and is not approved for commercial use.

Commercial SP (Fansidar®) from Roche will be used in this study. Each tablet contains 500 mg sulfadoxine/25 mg pyrimethamine.

5.3.1. Formulation and Packaging

AZ and CQ fixed-dose combination tablets AZCQ will be supplied in unit dose bottles. Fansidar® will be supplied by Pfizer with an ancillary label applied to the commercial container.

Pfizer will perform the labeling and distribution of the clinical supplies in accordance with applicable regulatory requirements.

5.3.2. Preparation and Dispensing

Only qualified personnel who are familiar with procedures that minimize undue exposure to them and to the environment should undertake the preparation, handling, and safe disposal of chemotherapeutic agents.

AZCQ will be dispensed to a subject for oral administration once daily for three days of therapy. Similarly, SP will be dispensed from the commercial package and administered orally for once daily.

5.3.3. Administration

All study drugs will be administered as open label therapy under supervision. Study medication will be dispensed by the Investigator or designee (Section 5.3.2). AZCQ and SP should not be administered on an empty stomach. Each dose should be administered with a glass of water.

- <u>AZCQ treatment</u>: first dose of each AZCQ treatment course will be administered under supervision during the ANC visit. Subsequent two doses will be administered at home by fieldworker(s).

090177e1811dafca\Approved\Approved On: 22-Feb-2010 14:41

- SP treatment: each dose of SP treatment course will be administered under supervision during the ANC visit.

Each subject will receive three IPTp treatment courses of AZCQ or SP during ANC visits at 4 - 8 week intervals. The first treatment course will be administered during the second trimester (14-26 weeks of gestation as confirmed by ultrasound). The last treatment course should be given to subjects prior to or during 36 weeks of gestation.

Any dose that is vomited within 30 minutes of administration will be repeated. If vomiting re-occurs after the re-dose, the subject will not receive further study treatments but will be referred to the investigator and will be given standard ANC care. If subjects in AZCQ group vomit after re-dose during first or second treatment course, they will be given additional course(s) of standard IPTp courses with SP. If vomiting on re-dosing of AZCQ happens during the home visits, subject will be advised to return to the study site within 7 days for appropriate treatment by the study physician. All subjects will be followed through Visit 6 on Day 28 (window Day 28 - Day 42) post delivery.

5.3.4. Compliance

The medication will be administered to the subject under supervision by investigators or site study team members during ANC visit or at home by the fieldworkers.

5.4. Drug Storage and Drug Accountability

The investigator, or an approved representative (eg, pharmacist), will ensure that all study drug is stored in a secured area, under recommended storage conditions and in accordance with applicable regulatory requirements. Prior to dispensing, all study medications should be stored under the conditions per label instructions. The investigator will be responsible for recording the receipt and usage of all drugs supplied and for ensuring the supervision (via the hospital pharmacist or fieldworker where relevant) of the storage and allocation of these supplies. To ensure adequate records, all study drugs will be accounted for in the case report form and drug accountability inventory forms as instructed by Pfizer.

The investigator or designee must maintain adequate records documenting the receipt, use, loss, or other disposition of the investigational products. Pfizer may supply drug accountability forms that must be used or may approve use of standard institution forms. In either case, the forms must identify the investigational products, including batch or code numbers, and account for its disposition on a subject-by-subject basis, including specific dates and quantities. The forms must be signed by the individual who dispensed the drug, and copies must be provided to Pfizer.

At the end of the trial, Pfizer will provide instructions as to disposition of any unused investigational products. If Pfizer authorizes destruction at the trial site, the investigator must ensure that the materials are destroyed in compliance with applicable environmental regulations, institutional policy, and any special instructions provided by Pfizer. Destruction must be adequately documented.

090177e1811dafca\Approved\Approved On: 22-Feb-2010 14:41

5.5. Concomitant Medication(s)

Medications consistent with the Package Inserts of AZ and CQ are allowed in the AZCQ group. However, to avoid interfering with the evaluation of the study drugs, subjects in the AZCQ group must not take SP during the course of the study unless they need to be switched to SP IPTp regimen consequent to their withdrawal from the AZCQ IPTp regimen because of any safety or tolerability issues related to AZCQ use. Subjects in the SP group must not take AZ and/or CQ during the participation of the study but other medications consistent with the Package Inserts of Fansidar are allowed. The medications that are contra-indicated during pregnancy should be avoided. All medications used by the subject at study entry or at any time during participation in the study must be recorded in the case report form. At each study visit, all medications taken since the last evaluation will be recorded.
While interactions with digoxin, ergot alkaloids, triazolam, carbamazepine, cyclosporine, hexobarbital and phenytoin have not been reported in clinical trials with AZ, they have been observed with macrolide products. Careful monitoring of subjects taking these medications is advised.

A single oral dose of AZ co-administered with nelfinavir at steady-state resulted in increased AZ serum concentrations. Although it is not necessary to adjust the dose of AZ when administered in combination with nelfinavir, close monitoring for known side effects of AZ (eg, liver enzyme abnormalities, hearing impairment) is warranted.

There have been reports which may indicate an increase in incidence and severity of adverse reactions when CQ is used with Fansidar as compared to the use of Fansidar alone (refer to Fansidar package insert). Fansidar is compatible with quinine and with antibiotics. However, antifolic drugs such as sulfonamides, trimethoprim, or trimethoprim-sulfamethoxazole combinations should not be used while the patient is receiving Fansidar for antimalarial prophylaxis. Fansidar has not been reported to interfere with antidiabetic agents.

If signs of folic acid deficiency develop, Fansidar should be discontinued. When recovery of depressed platelets or white blood cell counts in subjects with drug-induced folic acid deficiency is too slow, folinic acid (leucovorin) may be administered in doses of 5-15 mg intramuscularly daily for 3 days or longer.

Please refer to package inserts for SP, AZ, CQ and IB for AZCQ for further information regarding potential interactions between study drugs and other treatment agents.

5.6. Use of Anti-Malarial Treatment for Malaria in Pregnancy

Subjects presenting with clinical symptoms of malaria (fever >37.5°C, oral) and diagnosed (either by rapid diagnostic tests or microscopy) with malaria at any time during the study period will receive standard antimalarial treatment according to the local treatment guidelines for malaria in pregnancy, and will continue to participate in the study.

090177e1811dafca\Approved\Approved On: 22-Feb-2010 14:41

5.7. Treatment for Sexually Transmitted Infection

Subjects diagnosed with *T. pallidum* at the baseline Day 0 of Visit 1 or any time during the study period will receive standard dosing regimen of benzathine penicillin G consistent with local ANC guidelines.

Subjects presenting with clinical symptoms of *N. gonorrhoeae, C. trachomatis*, *T. vaginalis* at any time during the study period will receive appropriate therapies according to local ANC guidelines for treatment of these diseases in pregnancy.

Please refer to Section 5.5 (Concomitant Medications) and/or drug inserts for further information regarding potential interactions between study drugs and other treatment agents.

5.8. Treatment for Anemia in Pregnancy

Subjects diagnosed with anemia at the baseline, Day 0 of Visit 1 or any other visits during the study period will receive the standard treatment according to local ANC guidelines.

6. STUDY PROCEDURES

This study is exclusively conducted in pregnant women at ANC center or facility. Depending on when subjects enter the trial by gestational week, some subjects may have been to ANC prior to the first study visit. For such subjects, if results of required tests are available, then the test will not be repeated. The results will be transcribed to subject's Case Report Form as required following informed consent process is completed.

Refer to Section 7 (Assessments) for details on the procedures performed in the study.

6.1. Study Period

6.1.1. Visit 1: Baseline/Screening Visit/Treatment Course 1

Day 0: Antenatal Care (ANC) Visit/Assessment of Study Eligibility

After informed consent is obtained, the subject must have the following procedures performed prior to the start of therapy:

- Medical history;
- Obstetrical history;
- Concomitant treatment history;
- Urine pregnancy test (if not done at any prior ANC visit for this pregnancy);
- Urine analysis for glucose and proteins;
- Complete physical examination;

090177e1811dafca\Approved\Approved On: 22-Feb-2010 14:41

- Vital signs;
- Routine obstetric checkup;
- Obstetrical ultrasound examination (if urine pregnancy test is positive);
- Hemoglobin level (with HemoCue^{TM});
- *T. pallidum* screen test if not done at previous ANC check up;
- Nasopharyngeal swab test for macrolide resistant and penicillin resistant *Streptococcus pneumoniae* in approximately 600 subjects each in AZCQ and SP arms from 2 or more sites as feasible.

Day 0: Post Assessment of Eligibility/Treatment Course 1

Subjects who have met all inclusion/exclusion requirements and are eligible to be randomized will have the following procedures performed:

- Randomize to one of the study treatments;
- Administer treatment (AZCQ or SP);
- Assess adverse (AE) events;
- Provide LLINs.

Days 1 and 2: Home Visit (by the fieldworker)

- For subjects in the AZCQ group: the second and third dose of AZCQ will be administered to subject on Day 1 and Day 2 at home under supervision of the fieldworker. The installation of LLINs will be verified during the Day 1 visit; AE assessment at both home visits;
- For subjects in the SP group: home visit will be conducted on Day 2 to confirm the installation of LLINs and assess AEs.

6.1.2. Visit 2: Treatment Course 2

The time interval between the visits for treatment course 1 and the treatment course 2 will be 4-8 weeks. The subject will have the following procedures performed:

Day 0: ANC Visit:

- Concomitant treatment history;
- Vital signs;

090177e1811dafca\Approved\Approved On: 22-Feb-2010 14:41

- Routine obstetric checkup;
- Hemoglobin level (with HemoCue^{TM});
- Urine analysis for glucose and proteins;
- Drug administration (AZCQ or SP);
- AE assessments.

Days 1 and 2: Home Visit

- For subjects in the AZCQ group: the second and third dose of AZCQ will be administered to subject on Day 1 and Day 2 at home under supervision of the fieldworker. AE assessment at both home visits;
- For subjects in the SP group: home visit will be conducted on day 2 to assess AEs.

6.1.3. Visit 3: Treatment Course 3

The time interval between the visits for treatment course 2 and the treatment course 3 will be 4-8 weeks. The third treatment course should be given to subjects prior to or during 36 weeks of gestation. The subject will have the following procedures performed:

Day 0: ANC Visit

The subject will have the following procedures performed at ANC:

- Concomitant treatment history;
- Vital signs;
- Routine obstetric checkup;
- Hemoglobin level (with HemoCue^{TM});
- Urine analysis for glucose and proteins;
- Drug administration (AZCQ or SP);
- AE assessments.

090177e1811dafca\Approved\Approved On: 22-Feb-2010 14:41

090177e1811dafca\Approved\Approved On: 22-Feb-2010 14:41

Days 1 and 2: Home Visit

- For subjects in the AZCQ group: the second and third dose of AZCQ will be administered to subject on Day 1 and Day 2 at home by the fieldworker(s). AE assessment at both home visits;
- For subjects in the SP group: home visit will be conducted on day 2 to assess AEs.

6.1.4. Visit 4: Follow-up Visit at Weeks 36 to 38 of Gestation

The subject will have the following procedures performed at this visit at ANC:

- Concomitant treatment history;
- Vital signs;
- Routine Obstetric check up;
- Thick and thin peripheral blood smears (2 smears per subject);
- Hemoglobin level (with HemoCue™);
- Urine analysis for glucose & proteins;
- A dried blood blot for measurement of *P. falciparum* genetic markers. The blot will be sent for resistance tests only if the smear is positive:
  - CQ resistance markers in AZCQ treatment group: Pfcrt/Pfmdr1;
  - SP resistance markers in AZCQ treatment group: pfdhfr/pfdhps.
- A blood sample for *T. pallidum* tests: Rapid Plasma Reagin (RPR) test with confirmatory Treponema Pallidum Particle Agglutination (TPPA) assay;
- An endocervical swab for *C. trachomatis* and *N. gonorrhoeae* Test (Amplicor CT/NG PCR);
- A vaginal swab for bacterial vaginosis (Gram stain);
- A vaginal swab for *T. vaginalis* (refer to lab manual for details);
- AE assessments.

6.1.5. Visit 5: Follow-up Visit at Delivery

6.1.5.1. Follow Up Visit at Birth in Case of Hospital Delivery (At Birth)

Every attempt should be made to have the subject deliver in hospital setting and data will be collected at birth. The following procedures will be performed at the time of delivery in hospital:

For mothers:

- Pregnancy outcomes;
- Limited physical examination;
- Vital signs;
- Hemoglobin level (with HemoCue^{TM});
- Peripheral blood smears from the mother (2 smears per subject);
- Placental blood smears (2 smears per subject);
- Cord blood smears (2 smears per subject);
- A sample of the placenta tissue for histological examination as feasible (size: about 2cm x 2cm x 1cm);
- Concomitant treatment history;
- AE assessments.

For neonates:

- Birth weight of the neonate;
- General physical exam of the neonate;
- Lab diagnosis of ophthalmia neonatorum: collection of conjunctival exudates from neonates with purulent discharge for the lab test before the presumptive treatment is given. The standard empiric regimen of ophthalmia neonatorum presumptive treatment will be given to all neonates at birth per local treatment guidelines;
- AE assessments;
- Outcome research/resource utilization question.

090177e1811dafca\Approved\Approved On: 22-Feb-2010 14:41

6.1.5.2. Follow Up Visit at Birth in Case of Home Delivery (Within 2 Days of Subject Reporting Delivery)

Subjects will be encouraged to deliver in hospital. In case that the subject gives birth at home (home delivery), the subject will be asked to contact the study sites and report delivery within 24 hours of delivery. Fieldworker should visit the subject at home thereafter within 2 days of subject reporting delivery, and collect and/or transcribe data required in the study.

The following data will be collected and/or transcribed by fieldworker(s) if available.

For mothers:

- Pregnancy outcomes;
- Vital signs;
- Concomitant treatment history;
- AE assessments.

For neonates:

- Birth weight of the neonate;
- General physical exam of the neonate: the data will be collected or transcribed if available;
- AE assessments;
- Outcome research/resource utilization question.

6.1.6. Visit 6: Follow-up Visit on Day 28 Post Delivery (Window: Day 28 to Day 42)

The subject will have the following procedures performed at this visit at ANC:

- Concomitant treatment history;
- Limited physical examination;
- Vital signs;
- Hemoglobin level (with HemoCue^{TM});
- A nasopharyngeal swab for macrolide resistant and penicillin resistant *Streptococcus pneumoniae* from subjects who were tested at baseline;
- AE assessments;

090177e1811dafca\Approved\Approved On: 22-Feb-2010 14:41

- General physical exam of neonate;
- Outcome research/resource utilization data collection.

6.1.7. Follow-up Visit at About 6 Months after Last IPTp Course (in a Limited Number of Subjects Only)

- Concomitant treatment history;
- Limited physical examination;
- Vital signs;
- A nasopharyngeal swab for macrolide resistant and penicillin resistant *Streptococcus pneumoniae* from subjects who were tested at baseline.
- AE assessments.

6.1.8. Unscheduled Visit

In the event that subjects require an unscheduled visit (eg, for additional follow up due to any safety concerns, concomitant illness etc), the following assessments/procedures should be completed at a minimum:

- Symptom-directed physical exam with vital signs;
- Assessment of fetal viability or neonatal examination;
- AE assessments;
- Concomitant treatment history.

Subjects and/or their babies who develop clinical symptoms of malaria (fever >37.5°C, oral), or any other illness at any time during study period will be asked to report to the study physician immediately for further evaluation. **Any additional tests, procedures, treatments, and consultations may be conducted if recommended by local/national ANC guidelines.**

6.2. Subject Withdrawal

Subjects may withdraw from the study at any time at their own request, or they may be withdrawn at any time at the discretion of the investigator or sponsor for safety, behavioral, or administrative reasons. If a subject does not return for a scheduled visit, every effort should be made to contact the subject. In any circumstance, every effort should be made to document subject outcome, if possible. The investigator should inquire about the reason for withdrawal, request the subject to return all unused investigational product(s), request the subject to return for a final visit, if applicable, and follow-up with the subject regarding any unresolved adverse events.

090177e1811dafca\Approved\Approved On: 22-Feb-2010 14:41

If the subject withdraws from the study and also withdraws consent for disclosure of future information, no further evaluations should be performed and no additional data should be collected. The sponsor may retain and continue to use any data collected before such withdrawal of consent.

Unless subject withdraws the consent or leaves the town/village, she should continue in the study for follow-ups until Day 28 (window 28 to Day 42) post delivery. Subject may be **withdrawn from study treatment** due to safety concerns (eg, adverse events, significant laboratory abnormalities, contraindications of concomitant medications), but not from the clinical study per se. Subject will receive standard ANC care as per local ANC guideline. If a subject is withdrawn from the AZCQ IPTp regimen, she will be switched to the standard IPTp regimen as per local guidelines and will undergo regular follow up visits. If she receives an SP IPTp course during the study, she will be subsequently followed up as per follow up schedule for subjects receiving SP IPTp regimen. The circumstances and data surrounding conditions of withdrawal should be clearly documented in the case report form. The investigator must record the primary reason for discontinuation. A discontinuation must be reported immediately to the Pfizer medical monitor or his/her designated representative if it is due to an SAE.

7. ASSESSMENTS

All study related assessment will be performed after obtaining informed consent. Every effort should be made to ensure that the protocol required tests and procedures are completed as described. Field workers will be engaged at each site to maximize the dosing compliance and to minimize loss to follow-up visits. However it is anticipated that from time to time there may be circumstances, outside of the control of the investigator that may make it unfeasible to perform the test. In these cases the investigator will take all steps necessary to ensure the safety and well being of the subject. When a protocol required test can not be performed the investigator will document the reason for this and any corrective and preventive actions which he/she has taken to ensure that normal processes are adhered to as soon as possible. The study team will be informed of these incidents in a timely fashion.

The followings are key study assessments. The completed data collection and guidance are provided in CRF and its completion guideline. The detailed descriptions of the procedures (ie, sampling, handling, testing, storage, and shipment of the biological samples) and whether materials (ie test tubes and labels) will be supplied by Pfizer will be provided in a separate document as a lab manual.

7.1. Safety Assessments:

Safety and tolerability will be assessed by spontaneously reported adverse events, vital signs, physical examination, safety laboratory tests including hemoglobin and urine test for glucose and protein, and adverse pregnancy outcomes for mothers and by the general physical examination for the neonates through Day 28 (window: Day 28 to Day 42) post delivery.

- Adverse events assessments: refer to Section 8 (Adverse Event Reporting);

090177e1811dafca\Approved\Approved On: 22-Feb-2010 14:41

090177e1811dafca\Approved\Approved On: 22-Feb-2010 14:41

- Complete physical exam (including general appearance, throat, neck, thyroid, musculoskeletal, skin, lymph nodes, extremities, pulses, pulmonary, cardiac, abdominal, and neurological examination) at baseline or limited physical exam (including general appearance, brief pulmonary, cardiac, abdominal and neurological examination or symptom directed exams) at Visit 5, 6, and 7 from the activity schedule;

- Vital signs: include weight, sitting blood pressure, pulse rate and oral temperature;

- Routine obstetric checkup: include fetal height, fetal heart beat, fetal position;

- Obstetrical ultrasound: will be performed on Day 0 of Visit 1 only if urine pregnancy test is positive. The assessments include current gestational age (weeks), congenital abnormality, or any malformation.

- Laboratory tests:

  - Urine pregnancy test: will be performed if not done at any prior ANC visit for this pregnancy;

  - Hemoglobin levels: with HemoCue™, via finger stick or peripheral blood collection;

  - Urine analysis for glucose & proteins.

- Pregnancy outcomes: refer to Section 7.2 for details;

- General physical examination of the neonate at birth (within 2 days for home delivery) and at Day 28 (Day 28 - Day 42): weight, length, head circumference, APGAR scores (1/5 minutes at birth only), Ballard scores, congenital anomalies, clinical/hospital visits/stays, and other complications, etc;

- Concomitant treatment assessments.

7.2. Efficacy Assessments

- Adverse pregnancy outcomes: defined as live-borne neonate (singleton) with LBW (<2,500g), premature births (<37 weeks), abortion (≤28 weeks), still birth (>28 weeks), lost to follow-up prior to termination of pregnancy or delivery, or missing birth weight of the neonates. Following are some details:[114]

  - LBW: defines as birth weight less than 2500 g (up to and including 2499g);

  - Abortion: defines as a pregnancy loss before 28 completed weeks of gestation (≤28 weeks);

  - Stillbirth: birth of a baby showing no signs of life. It defines as a pregnancy loss after 28 weeks of gestation (>28 weeks);

  - Premature births: the neonate born prior to 37 weeks of gestation (<37 weeks);

  - Lost to follow-up prior to termination of pregnancy or delivery.

  The sites are asked to make every attempt to follow up subjects through Day 28 post delivery unless the consent is withdrawn.

- Neonate outcomes at birth: birth weight, length, APGAR scores (1/5 minutes), Ballard scores, congenital abnormalities, perinatal and neonatal deaths, ophthalmia neonatorum (lab diagnosis):

    - Birth weight: The weight of a neonate determined immediately after delivery or as soon as feasible thereafter. If home delivery, the subject should contact the study site as soon as possible when the delivery occurs. Field workers will visit home as early as possible within 2 days of subject reporting delivery. Digital weight scale will be used for accurate readings;

    - Ophthalmia neonatorum: laboratory diagnosis will be performed among neonates with purulent discharge. A sample of conjunctival exudates will be taken on a sterile cotton swab before the treatment is given. The collected sample will be sent to a central lab to test *N. gonorrhoeae* and *C. trachomatis* using PCR assay (Amplicor CT/NG, Roche). The standard presumptive treatment/prophylaxis for ophthalmia neonatorum will be given to neonates at birth per local treatment guidelines;

- Hemoglobin levels: HemoCue™, via finger stick or peripheral blood collection;

- Diagnosis of pre-eclampsia from Week 20 to delivery:

  (1) Systolic blood pressure of at least 140 mmHg and/or diastolic blood pressure of at least 90 mmHg, measured on two separate readings taken at least 4 hours apart, AND

  (2) Proteinuria at least 300 mg protein in a 24 hour urine collection.

- Peripheral blood smears at Week 36 - 38 of gestation and at birth from subjects with hospital delivery: Thick and thin peripheral blood smears prepared using standard Giemsa staining method for parasite identification and count if positive. The blood smears will be read in a blinded fashion (blinded to study treatment and the reading of other microscopists);

090177e1811dafca\Approved\Approved On: 22-Feb-2010 14:41

- Placental blood smear: will be prepared from placental blood at birth from subjects who deliver at hospital. Thick and thin blood smears (2 smears per subject) will be prepared using standard Giemsa staining method for parasite identification and count. The blood smears will be read by at least two microscopists in a blinded fashion (blinded to study treatment and the reading of other microscopists);

- Placental histology: a sample of placenta tissue (size: about 2cm x 2cm x 1cm) will be collected at birth from the subjects who deliver at hospital for histological examination when feasible. Refer to lab manual for details;

- Cord blood smear: will be prepared from cord blood at birth from subjects with hospital delivery. Thick and thin blood smears (2 smears per subject) will be prepared using standard Giemsa staining method for parasite identification and count. The blood smears will be read by at least two microscopists in a blinded fashion (blinded to study treatment and the reading of other microscopists).

- Sexually Transmitted Infections (STIs)/Reproductive Tract Infections (RTIs) laboratory tests:

  A. *T. pallidum* test:

    - At Baseline: *T. pallidum* screening test: this will be a blood screening test using Rapid Plasma Reagin (RPR) method. It is often normally conducted at the first ANC visit. If the result is available, it will be transcribed to study case report form. If it is not done at previous ANC check up, the *T. pallidum* screen test will be conducted in consistent with local/national ANC guideline. The test result is not required at the enrollment.

    - At Weeks 36 -38 of gestation: blood samples (~0.5 ml) will be collected and serum will be used for the tests:

      - Rapid Plama Reagin (RPR): a non-trponemal specific serological screening test. Positive test indicates a high likelihood of *T. pallidum* infection, although not necessarily current, active disease;

      - Treponema Pallidum Particil Agglutination Assay (TPPA): a confirmatory test when RPR is positive. This is a treponemal test for the serologic detection of antibodies to the various species including *T. pallidum*. The test is used to confirm the reactive results of a non-treponemal screening test for *T. pallidum* or as a diagnostic test in patients with a nonreactive nontreponemal test but with signs or symptoms suggestive of late syphilis.

  B. *N. gonorrhoeae* and *C. trachomatis* test at Weeks 36 - 38 of gestation: an endocervical swab will be collected and PCR assay (Amplicor CT/NG, Roche) will be used for analysis;

090177e1811dafca\Approved\Approved On: 22-Feb-2010 14:41

C. Bacterial vaginosis at Weeks 36 - 38 of gestation: a vaginal swab will be collected for Gram staining;

D. Trichomonas vaginalis at Weeks 36 - 38 of gestation: a vaginal swab will be collected for the lab test. Refer to lab manual for details.

- Clinical presentations/diagnosis at any time during the study period through Day 28 post delivery. This information will be recorded on related CRF pages.
    - Clinical episode of malaria: subjects presenting with clinical symptoms of malaria (fever >37.5°C, oral) and diagnosed (either by rapid diagnostic tests or microscopy) with malaria;
    - Sexual Transmitted Disease (STIs): include *T. pallidum*, *N. gonorrhoeae*, *C. trachomatis;*
    - Bacterial infections including pneumonia and other lower respiratory tract infections.
- Subjects requiring additional treatments for malaria during the study period following the first dose (diagnosed based on clinical presentation and/or lab test results);
- *P.f.* genetic resistance marker tests at Weeks 36 - 38 of gestation: A dried blood blot will be collected from all subjects but tests will be done only from subjects with parasitemia. Polymerase Chain Reaction (PCR) assay will be used and following markers will be tested:
    - CQ resistance markers in AZCQ treatment group: Pfcrt/Pfmdr1;
    - SP resistance markers in SP treatment group: pfdhfr/ pfdhps.
- Macrolide and Penicillin resistance tests for *Strep. pneumoniae*:
    - Nasopharyngeal swabs will be collected from approximately 600 subjects each in AZCQ and SP arms from at least 2 sites at three time points: (1) at baseline (Visit 1), (2) at Day 28 - Day 42 post delivery (Visit 6), and (3) at about 6 months post last IPTp course (Visit 7). This is to test *Streptococcus pneumoniae* sensitivity against three antibiotics: azithromycin, erythromycin and penicillin. Refer to lab manual for details.

7.3. Outcome Research Assessment

Economic impact will be assessed from the health system and provider perspective, as measured by in-hospital and out-of-hospital healthcare utilization consumed by the newborn/infant and the mother due to low birth weight, infections and health complications. Healthcare resource utilization assessments will be done at delivery (within 2 days for home delivery) and on Day 28 post delivery.

090177e1811dafca\Approved\Approved On: 22-Feb-2010 14:41

8. ADVERSE EVENT REPORTING

8.1. Adverse Events

All observed or volunteered adverse events regardless of treatment group or suspected causal relationship to the investigational product(s) will be reported as described in the following sections.

For all adverse events, the investigator must pursue and obtain information adequate both to determine the outcome of the adverse event and to assess whether it meets the criteria for classification as a serious adverse event requiring immediate notification to Pfizer or its designated representative. For all adverse events, sufficient information should be obtained by the investigator to determine the causality of the adverse event. The investigator is required to assess causality. For adverse events with a causal relationship to the investigational product, follow-up by the investigator is required until the event or its sequelae resolve or stabilize at a level acceptable to the investigator, and Pfizer concurs with that assessment.

8.2. Reporting Period

For serious adverse events, the reporting period to Pfizer or its designated representative begins from the time that the subject provides informed consent, which is obtained prior to the subject's participation in the study, ie, prior to undergoing any study-related procedure and/or receiving investigational product, **through Day 42 and including 39 calendar days after the last administration of the investigational product or until the last study visit (which ever is later).** Any serious adverse event occurring any time after the reporting period must be promptly reported if a causal relationship to investigational product is suspected.

Adverse events (serious and non-serious) should be recorded on the CRF from the time the subject has taken at least one dose of study treatment through last subject visit.

8.3. Definition of an Adverse Event

An adverse event is any untoward medical occurrence in a clinical investigation subject administered a product or medical device; the event need not necessarily have a causal relationship with the treatment or usage. Examples of adverse events include but are not limited to:

- Abnormal test findings;
- Clinically significant symptoms and signs;
- Changes in physical examination findings;
- Hypersensitivity;

090177e1811dafca\Approved\Approved On: 22-Feb-2010 14:41

- Progression/worsening of underlying disease.

Additionally, they may include the signs or symptoms resulting from:

- Drug overdose;
- Drug withdrawal;
- Drug abuse;
- Drug misuse;
- Drug interactions;
- Drug dependency;
- Extravasation.

8.4. Abnormal Test Findings

The criteria for determining whether an abnormal objective test finding should be reported as an adverse event are as follows:

- Test result is associated with accompanying symptoms; and/or
- Test result requires additional diagnostic testing or medical/surgical intervention; and/or
- Test result leads to a change in study dosing or discontinuation from the study, significant additional concomitant drug treatment, or other therapy; and/or
- Test result is considered to be an adverse event by the investigator or sponsor.

Merely repeating an abnormal test, in the absence of any of the above conditions, does not constitute an adverse event. Any abnormal test result that is determined to be an error does not require reporting as an adverse event.

8.5. Serious Adverse Events

A serious adverse event or serious adverse drug reaction is any untoward medical occurrence at any dose **for mothers or fetus** that:

- Results in death;
- Is life-threatening (immediate risk of death);
- Requires inpatient hospitalization or prolongation of existing hospitalization;

090177e1811dafca\Approved\Approved On: 22-Feb-2010 14:41

- Results in persistent or significant disability/incapacity;
- Results in congenital anomaly/birth defect.

Medical and scientific judgment should be exercised in determining whether an event is an important medical event. An important medical event may not be immediately life-threatening and/or result in death or hospitalization. However, if it is determined that the event may jeopardize the subject and/or may require intervention to prevent one of the other adverse event outcomes, the important medical event should be reported as serious.

Examples of such events are intensive treatment in an emergency room or at home for allergic bronchospasm; blood dyscrasias or convulsions that do not result in hospitalization; or development of drug dependency or drug abuse.

8.6. Hospitalization

Adverse events reported from studies associated with hospitalization or prolongation of hospitalization, are considered serious. Any initial admission (even if less than 24 hours) to a healthcare facility meets these criteria. Admission also includes transfer within the hospital to an acute/intensive care unit (eg, from the psychiatric wing to a medical floor, medical floor to a coronary care unit, neurological floor to a tuberculosis unit).

Hospitalization does not include the following:

- Rehabilitation facilities;
- Hospice facilities;
- Respite care (eg, caregiver relief);
- Skilled nursing facilities;
- Nursing homes;
- Routine emergency room admissions;
- Same day surgeries (as outpatient/same day/ambulatory procedures).

Hospitalization or prolongation of hospitalization in the absence of a precipitating, clinical adverse event is not in itself a serious adverse event. Examples include:

- Admission for treatment of a pre-existing condition not associated with the development of a new adverse event or with a worsening of the pre-existing condition (eg, for work-up of persistent pre-treatment lab abnormality);
- Social admission (eg, subject has no place to sleep);
- Administrative admission (eg, for yearly physical exam);

090177e1811dafca\Approved\Approved On: 22-Feb-2010 14:41

- Protocol-specified admission during a study (eg, for a procedure required by the study protocol);
- Optional admission not associated with a precipitating clinical adverse event (eg, for elective cosmetic surgery);
- Pre-planned treatments or surgical procedures should be noted in the baseline documentation for the entire protocol and/or for the individual subject.

Diagnostic and therapeutic non-invasive and invasive procedures, such as surgery, should not be reported as adverse events. However, the medical condition for which the procedure was performed should be reported if it meets the definition of an adverse event. For example, an acute appendicitis that begins during the adverse event reporting period should be reported as the adverse event, and the resulting appendectomy should be recorded as treatment of the adverse event.

8.7. Severity Assessment

Table 3. Severity Assessment

If required on the adverse event case report forms, the investigator will use the adjectives MILD, MODERATE, or SEVERE to describe the maximum intensity of the adverse event. For purposes of consistency, these intensity grades are defined as follows:	
MILD	Does not interfere with subject's usual function.
MODERATE	Interferes to some extent with subject's usual function.
SEVERE	Interferes significantly with subject's usual function.

Note the distinction between the severity and the seriousness of an adverse event. A severe event is not necessarily a serious event. For example, a headache may be severe (interferes significantly with subject's usual function) but would not be classified as serious unless it met one of the criteria for serious adverse events, listed above.

8.8. Causality Assessment

The investigator's assessment of causality must be provided for all adverse events (serious and non-serious); the investigator must record the causal relationship in the CRF, as appropriate, and report such an assessment in accordance with the serious adverse reporting requirements if applicable. An investigator's causality assessment is the determination of whether there exists a reasonable possibility that the investigational product caused or contributed to an adverse event. If the investigator does not know whether or not investigational product caused the event, then the event will be handled as "related to investigational product" for reporting purposes, as defined by the Sponsor (See Section on Reporting Requirements). If the investigator's causality assessment is "unknown but not related to investigational product," this should be clearly documented on study records.

090177e1811dafca\Approved\Approved On: 22-Feb-2010 14:41

In addition, if the investigator determines a serious adverse event is associated with study procedures, the investigator must record this causal relationship in the source documents and CRF, as appropriate, and report such an assessment in accordance with the serious adverse event reporting requirements, if applicable.

8.9. Exposure During Pregnancy

This is a study exclusively in pregnant women. Enrolled subjects will be asymptomatic pregnant women who will receive study drugs for preventive treatment of malaria. All pregnancy-related AEs and pregnancy outcomes will be recorded on CRF pages as required clinical study data. Since all the information generally included on an Exposure In Utero (EIU) form will be collected on CRF paper, a separate EIU form is not required.

Subjects will be followed up to Day 28 post delivery (window: Day 28 to Day 42) for pregnancy outcomes or until pregnancy termination (ie, induced abortion). The reason(s) for an induced abortion should be specified.

If the outcome of the pregnancy meets the criteria for immediate classification as a serious adverse event (ie, spontaneous abortion, stillbirth, neonatal death, or congenital anomaly [including that in an aborted fetus, stillbirth or neonatal death]), the investigator should follow the procedures for reporting serious adverse events.

In the case of a live birth, the "normality" of the newborn can be assessed at the time of birth, or as soon thereafter as possible, and at Day 28 post delivery (window: Day 28 to Day 42). The "normality" of an aborted fetus can be assessed by gross visual inspection, unless pre-abortion test findings are suggestive of a congenital anomaly.

Additional information about pregnancy outcomes that are classified as serious adverse events follows:

- "Spontaneous abortion" includes miscarriage and missed abortion.
- All neonatal deaths that occur within 1 month of birth should be reported, without regard to causality, as serious adverse events. In addition, any infant death after 1 month that the investigator assesses as possibly related to the Exposure in Utero to the investigational medication should be reported.

Additional information regarding the Exposure in Utero may be requested by the investigator. Further follow-up of birth outcomes will be handled on a case-by-case basis (eg, follow-up on preterm infants to identify developmental delays). In the case of paternal exposure, the investigator must obtain permission from the subject's partner in order to conduct any follow-up or collect any information.

8.10. Withdrawal Due to Adverse Events (See also Section on Subject Withdrawal)

Withdrawal due to adverse event should be distinguished from withdrawal due to insufficient response, according to the definition of adverse event noted earlier, and recorded on the appropriate adverse event CRF page.

090177e1811dafca\Approved\Approved On: 22-Feb-2010 14:41

When a subject withdraws due to a serious adverse event, the serious adverse event must be reported in accordance with the reporting requirements defined below.

Unless a subject withdraws consent or leaves the town/village, she may be withdrawn from the study treatment but should continue in the study as we must follow them up for pregnancy outcome up to Day 28 post delivery.

8.11. Eliciting Adverse Event Information

The investigator is to report all directly observed adverse events and all adverse events spontaneously reported by the study subject. In addition, each study subject will be questioned about adverse events.

8.12. Reporting Requirements

Each adverse event is to be assessed to determine if it meets the criteria for serious adverse events. If a serious adverse event occurs, expedited reporting will follow local and international regulations, as appropriate.

8.12.1. Serious Adverse Event Reporting Requirements

If a serious adverse event occurs, Pfizer is to be notified within 24 hours of awareness of the event by the investigator. In particular, if the serious adverse event is fatal or life-threatening, notification to Pfizer must be made immediately, irrespective of the extent of available adverse event information. This timeframe also applies to additional new information (follow-up) on previously forwarded serious adverse event reports as well as to the initial and follow-up reporting of Exposure in Utero cases.

This timeframe of reporting within 24 hours also applies for the reporting of any medical device complaint.

In the rare event that the investigator does not become aware of the occurrence of a serious adverse event immediately (eg, if an outpatient study subject initially seeks treatment elsewhere), the investigator is to report the event within 24 hours after learning of it and document the time of his/her first awareness of the adverse event.

For all serious adverse events, the investigator is obligated to pursue and provide information to Pfizer in accordance with the timeframes for reporting specified above. In addition, an investigator may be requested by Pfizer to obtain specific additional follow-up information in an expedited fashion. This information may be more detailed than that captured on the adverse event case report form. In general, this will include a description of the adverse event in sufficient detail to allow for a complete medical assessment of the case and independent determination of possible causality. Information on other possible causes of the event, such as concomitant medications and illnesses must be provided. In the case of a subject death, a summary of available autopsy findings must be submitted as soon as possible to Pfizer or its designated representative.

090177e1811dafca\Approved\Approved On: 22-Feb-2010 14:41

In addition, the Investigator will follow all applicable local and national safety reporting guidelines and regulations for notification to their local ethics and regulatory bodies.

8.12.2. Non-Serious Adverse Event Reporting Requirements

All adverse events will be reported on the adverse event page(s) of the CRF. It should be noted that the form for collection of serious adverse event information is not the same as the adverse event CRF. Where the same data are collected, the forms must be completed in a consistent manner. For example, the same adverse event term should be used on both forms. Adverse events should be reported using concise medical terminology on the CRFs as well as on the form for collection of serious adverse event information.

8.12.3. Medical Device Complaint Reporting Requirements

Not applicable.

8.12.4. Sponsor Reporting Requirements to Regulatory Authorities

Adverse events reporting, including suspected serious unexpected adverse reactions, will be carried out in accordance with applicable local regulations.

9. DATA ANALYSIS/STATISTICAL METHODS

Detailed methodology for summarization and statistical analyses of the data collected in this study will be documented in a Statistical Analysis Plan, which will be maintained by the sponsor. This document may modify the plans outlined in the protocol. However, any major modifications of the primary endpoint definition and/or its analysis will also be reflected in a protocol amendment.

9.1. Sample Size and Power Determination

Sample size will be determined using the observed primary endpoint pooled across both arms following a blinded review when approximately 1000 subjects achieve the primary endpoint.

The underlying true incidence of the primary endpoint in the control group is unknown, but has a substantial impact on the required sample size. To manage the uncertainty, the proportion of subjects experiencing a sub-optimal pregnancy outcome will be estimated from the current trial, when approximately 1000 subjects achieve the endpoint. This evaluation will be done in a blinded manner by the Sponsor study team, though the trial is open label. The underlying incidence will be estimated by the pooled (blinded) overall proportion of subjects experiencing a sub-optimal pregnancy outcome without regard to treatment group.

Total sample size will be determined using Table 4. For example, if the observed proportion of subjects experiencing a sub-optimal pregnancy outcome is found to be 0.30, then the total sample size will be 4206.

090177e1811dafca\Approved\Approved On: 22-Feb-2010 14:41

Table 4. Sample Size Determination

Observed Pooled Proportion for The Primary Endpoint at N=1000	≤0.28	>0.28 to ≤0.32	>0.32 to ≤0.36	>0.36 to ≤0.40	>0.40
Total Sample Size Required	5044	4206	3552	3030	2602

Note that the total sample size required is calculated by employing a group sequential design as detailed in Section 9.5 with the upper bound of each interval assumed to be the true underlying proportion for the SP group.

Due to the adaptive nature of the sample size determination, operating characteristics of the study design in terms of power and Type I error rate were evaluated using simulations. Probabilities for early stopping for efficacy and futility at each of the interim analyses were evaluated as well. The design assumed a 20% risk reduction in the primary endpoint (relative risk=0.80, AZCQ/SP). A 23% risk reduction (relative risk=0.77, AZCQ/SP) was assumed for LBW (key secondary) given that early stopping for efficacy will only occur if both endpoints are positive. Type I error rates were simulated under the null hypothesis of no difference. Each scenario under the alternative hypothesis was run with 100,000 simulation iterations. One million iterations were used under the null hypothesis for each stated true proportion, for the primary endpoint.

9.1.1. Simulated Power Under a 20% Risk Reduction

Simulated powers and futility probabilities assuming a 20% risk reduction (relative risk=0.80, AZCQ/SP) are presented in Table 5. The powers are approximately 90% for the true SP proportions ≥28%, which is the range for SP expected in this study. Power for LBW (relative risk=0.77) ranges between 82% and 88% approximately.

Table 5. Power and Futility Probability: 20% Risk Reduction

**True Proportion for the Primary Endpoint**		**Probability of Claiming Efficacy (%)**			**Probability of Declaring Futility (%)**			**Average Sample Size**
SP	AZCQ	1st IA	2nd IA	**Power**	1st IA	2nd IA	Beta (1-Power)	
0.24	0.192	9	17	**81**	1	3	19	4492
0.28	0.224	15	23	**90**	<1	1	10	4267
0.32	0.256	18	26	**92**	<1	1	8	3677
0.36	0.288	20	26	**92**	<1	1	8	3120
0.40	0.320	21	27	**93**	<1	1	7	2679
0.44	0.352	22	27	**94**	<1	1	6	2307

090177e1811dafca\Approved\Approved On: 22-Feb-2010 14:41

Simulated Type I Error Rate Under the Null Hypothesis

Simulated Type I error rates for the primary endpoint are presented in Table 6. The Type I error rate is preserved at 0.0625% (alpha=0.000625 1-sided) within an allowable margin of error given the 1 million iterations performed.

Table 6. Type I Error Rate

True Proportion for the Primary Endpoint		Probability of Claiming Efficacy (%)			Probability of Declaring Futility (%)		Average Sample Size
SP	AZCQ	1st IA	2nd IA	**Type I Error Rate**	1st IA	2nd IA	
0.24	0.24	0.00340	0.00470	**0.06040**	73	22	2862
0.28	0.28	0.00430	0.00600	**0.05930**	74	21	2628
0.32	0.32	0.00310	0.00740	**0.06300**	74	21	2207
0.36	0.36	0.00320	0.00530	**0.05620**	73	22	1876
0.40	0.40	0.00260	0.00580	**0.06010**	74	21	1601
0.44	0.44	0.00350	0.00820	**0.05770**	74	21	1478

9.2. Efficacy Analysis

The primary efficacy analysis population (ie, Primary Analysis Set) will be intent-to-treat (ITT) for all endpoints. It will consist of subjects who are randomized, have received at least one dose of study medication (Day 0 at Visit 1 is considered the first dose of study medication), and have a single fetus. Subjects without the required data needed to compute an endpoint will be treated as missing. Analyses will also be performed for as per protocol analysis set, considered secondary. A per protocol analysis set will consist of all ITT subjects who have been compliant with study medication and will exclude subjects who have a neonate birth-weight measured greater than 7 days after birth if they do not already meet the definition of sub-optimal pregnancy outcome, and also exclude subjects who withdraw from study drug and switch to standard of care IPTp treatment. Compliance with study medication will consist of at least 6 of the 9 AZCQ daily doses, and at least 2 of the 3 SP daily doses. Once again, subjects without the required data needed to compute an endpoint will be treated as missing. Sensitivity analyses will be defined for both the ITT and PP populations.

Treatment group comparisons for the primary endpoint will use an overall alpha level of 0.00125 2-sided (equivalent to 0.000625 1-sided), subject to the alpha spending function specified in Section 9.5. Treatment group comparisons for the secondary endpoints will use an overall alpha level of 0.05 2-sided (equivalent to 0.025 1-sided), also subject to the same alpha spending function.

090177e1811dafca\Approved\Approved On: 22-Feb-2010 14:41

9.2.1. Analysis of Primary Endpoint

The primary efficacy endpoint of the trial is the presence of subjects with a sub-optimal pregnancy outcome defined as any of the following: live-borne neonate (singleton) with low birth-weight (or LBW for short, defined as live birth weight <2,500g), premature births (<37 weeks), abortion (≤28 weeks), still birth (>28 weeks), lost to follow-up prior to termination of pregnancy or delivery, or missing birth weight of the neonate. The primary analysis set will be the ITT, and will exclude multiple gestations. Secondary analyses will also be repeated using the per protocol analysis set.

Percentage of subjects with sub-optimal pregnancy outcome will be estimated for each treatment group. The following descriptive statistics will be available:

- Sample size (n) per group;
- Number of subjects with sub-optimal pregnancy outcome (m);
- Proportion of subjects with sub-optimal pregnancy outcome:

  $$\hat{p} = \frac{m}{n}$$

- Two sided CI will be formed for the proportion of subjects using the normal approximation to the binomial:

  $$\hat{p} \pm z_{1-\alpha/2}\sqrt{\frac{\hat{p}(1-\hat{p})}{n}}$$

The final results will be expressed in percentages, ie, proportions x 100. If the lower confidence limit is <0, it will be set to 0. If the upper confidence limit is >100%, it will be set to 100%.

The Mantel-Haenszel estimate[115, 116] of the common risk ratio will be computed using SAS Proc Freq, adjusted for the randomization strata. The risk ratio will be the proportion of AZCQ subjects with sub-optimal pregnancy outcome over the proportion of SP subjects with sub-optimal pregnancy outcome. The estimated variance for $ln(RR_{MH})$ given by Greenland and Robins[117] will be utilized. The two-sided p-value will be calculated using the Wald test statistic on the natural log risk ratio scale.

Additional analysis models for the primary endpoint will be used to examine the interaction of a range of effects with treatment group. These effects include randomization strata, HIV status at baseline, age, and country. An alpha level of 0.05 2-sided will be used for these additional analyses.

090177e1811dafca\Approved\Approved On: 22-Feb-2010 14:41

9.2.2. Analysis of Secondary Endpoints

The proportion of subjects with LBW is a key secondary endpoint. It is defined as the following: live-borne neonate (singleton) low birth-weight (or LBW for short, defined as live birth weight <2,500g). A secondary analysis will also be repeated using the per protocol analysis set.

All secondary efficacy endpoints that are dichotomous, expressed as a proportion, will be summarized and analyzed using the same methods as for the primary endpoint. Secondary efficacy endpoints that are not dichotomous will be analyzed using analysis of covariance (ANCOVA) when a baseline value is available, and analysis of variance (ANOVA) otherwise. The ANCOVA model will contain baseline as a covariate, as well as factors for treatment group and randomization strata. The ANOVA model will contain factors for treatment group and randomization strata.

For subjects with nasopharyngeal swabs positive for macrolide resistant and penicillin resistant *Strep. pneumoniae* (to be performed in about 600 subjects in each AZCQ arm and SP arm from at least 2 sites), the proportion positive at Baseline, Day 28-42 post delivery, and at 6 months post last AZCQ IPTp course will be computed. A listing of these subjects will also be provided.

9.3. Outcome Research Analysis

Health economic outcomes will be assessed in terms of the health care resource utilization consumed by newborns/infants and mothers due to low birth weight, anemia, sexually transmitted diseases or sub-optimal pregnancy outcome. Measures of resource utilization will include in-hospital and out-of-hospital services for newborn/infant and mother, and associated care.

In-hospital services will include number of times of hospitalization, length of hospital stay and the numbers and durations of related admissions/re-admissions. Out-of-hospital services will include local health clinic/physician's office/outpatient hospital clinic including emergency room (without having to be admitted) to attend to a health complication(s). Data will be summarized descriptively by treatment group.

Analysis of health economics outcomes will include an analysis of the number of hospitalization times and durations of hospital stays as well as number of outpatient hospital visits. Statistical methods will include the Wilcoxon or t-test for durations of total hospital stays and number of outpatient hospital visits. Descriptive statistics will be used in summarizing the results of the individual items in the healthcare resource utilization questionnaire. Cost analysis of these items may be performed separately, outside the scope of the current analysis plan.

Contributing to the health economic impact endpoint is the physician/subject questionnaire.

090177e1811dafca\Approved\Approved On: 22-Feb-2010 14:41

9.4. Safety Analysis

Adverse events, concomitant medications, laboratory safety tests including hemoglobin and urine test for glucose and protein, physical examinations, and vitals signs will be collected for each subject during the study according to the schedule of assessments. Standard safety reporting tables will summarize and list the safety data.

Each adverse event will be counted once according to the date of onset. If the adverse event onset was prior to the first dose of study drug and the event does not increase in severity after initiation of study drug, the adverse event is then considered to be a pre-treatment adverse event and will not be counted in the treatment-emergent adverse event incidence tables. If the onset is prior to the first dose of study drug and the severity increases thereafter, the event is counted as a treatment-emergent adverse event. An adverse event with onset after the first dose of study drug will be counted as a treatment-emergent adverse event. This rule is consistent with the treatment-emergent signs and symptoms (TESS) convention for counting adverse events.

9.5. Interim Analysis

The study will employ a group sequential design with three unblinded analyses conducted at 50%, 70% and 100% of information time (percentage of subjects with data for the primary endpoint). Group sequential stopping rules will be used to stop the trial early for efficacy and futility (futility is non binding) at each of the 2 sequential analyses.

The overall alpha level will be 1-sided 0.000625 and the power will be approximately 90% (See Section 9.1) for establishing superiority of AZCQ over SP in proportion of subjects with sub-optimal pregnancy outcome (primary endpoint). Secondary efficacy endpoints will use an overall alpha level of 1-sided 0.025. The study was designed using the EAST software Version 5.1.

To protect the Type I error rate, the gamma (-4) function will be used for alpha spending for all efficacy endpoints. Similarly, the gamma (-6) function will be used for beta spending for the primary endpoint only. Early stopping for efficacy will only occur if both the proportion of subjects with sub-optimal pregnancy outcome (primary endpoint) and the proportion of subjects with LBW (key secondary) is statistically significantly lower in the AZCQ group compared to SP, as evaluated by the relative risk (primary analysis). Early stopping for futility will only be based on the primary endpoint.

The EAST software will be used to monitor the sequential analyses. It is important to note that the critical values for the stopping boundaries will depend on the percentage of subjects actually in each sequential analysis (the actual observed percentage of information). The new critical values will be calculated within EAST subject to the pre-defined alpha and beta spending functions specified for the design, in order to maintain the appropriate overall power and significance level.

090177e1811dafca\Approved\Approved On: 22-Feb-2010 14:41

The sequential analyses will use unblinded treatment codes, and be prepared by a group within Pfizer but external to the study team. An Independent Data Monitoring Committee (IDMC) external to Pfizer will then be used to formally make decisions on stopping or continuing the study based on the interim results, the predefined stopping regions, and the interim monitoring output from EAST. The membership of the IDMC and its charter, as well as the procedures for performing the interim analyses will be finalized and documented prior to the first interim analysis.

9.6. Independent Data Monitoring Committee (IDMC)

An IDMC external from Pfizer will review interim analyses data and oversee evaluation of emerging safety data on an ongoing basis. The full composition of the IDMC and the guidelines for oversight of this study will be specified in the IDMC charter.

A sponsor statistical programmer external to the study team will prepare results necessary for the IDMC review and provide the results directly to the IDMC. The sponsor study personnel who are involved in the day to day operation of the trial will remain blinded to the results provided to the IDMC until the database lock for the final analysis.

10. QUALITY CONTROL AND QUALITY ASSURANCE

During study conduct, Pfizer or its agent will conduct periodic monitoring visits to ensure that the protocol and GCPs are being followed. The monitors may review source documents to confirm that the data recorded on CRFs is accurate. The investigator and institution will allow Pfizer monitors or its agents and appropriate regulatory authorities direct access to source documents to perform this verification.

The study site may be subject to review by the Institutional Review Board (IRB)/Independent Ethics Committee (IEC), and/or to quality assurance audits performed by Pfizer, or companies working with or on behalf of Pfizer, and/or to inspection by appropriate regulatory authorities.

It is important that the investigator(s) and their relevant personnel are available during the monitoring visits and possible audits or inspections and that sufficient time is devoted to the process.

11. DATA HANDLING AND RECORD KEEPING

11.1. Case Report Forms/Electronic Data Record

As used in this protocol, the term Case Report Form (CRF) should be understood to refer to either a paper form or an electronic data record or both, depending on the data collection method used in this study.

A CRF is required and should be completed for each included subject. The completed original CRFs are the sole property of Pfizer and should not be made available in any form to third parties, except for authorized representatives of Pfizer or appropriate regulatory authorities, without written permission from Pfizer.

090177e1811dafca\Approved\Approved On: 22-Feb-2010 14:41

The investigator has ultimate responsibility for the accuracy, authenticity, and timely collection and reporting of all clinical, safety, laboratory data entered on the CRFs and any other data collection forms. The CRFs must be signed by the investigator or by an authorized staff member to attest that the data contained on the CRFs is true. Any corrections to entries made in the CRFs, source documents must be dated, initialed and explained (if necessary) and should not obscure the original entry".

In most cases the source documents are the hospital or the physician's chart. In these cases, data collected on the CRFs must match those charts.

In some cases, the CRF may also serve as the source document. In these cases, a document should be available at the investigator's site as well as at Pfizer and clearly identify those data that will be recorded in the CRF, and for which the CRF will stand as the source document.

11.2. Record Retention

To enable evaluations and/or audits from regulatory authorities or Pfizer, the investigator agrees to keep records, including the identity of all participating subjects (sufficient information to link records, eg, CRFs and hospital records), all original signed informed consent forms, copies of all CRFs, serious adverse event forms, source documents, and detailed records of treatment disposition, and adequate documentation of relevant correspondence (eg, letters, meeting minutes, telephone calls reports). The records should be retained by the investigator according to ICH, local regulations, or as specified in the Clinical Study Agreement, whichever is longer.

If the investigator becomes unable for any reason to continue to retain study records for the required period (eg, retirement, relocation), Pfizer should be prospectively notified. The study records must be transferred to a designee acceptable to Pfizer, such as another investigator, another institution, or to Pfizer. The investigator must obtain Pfizer's written permission before disposing of any records, even if retention requirements have been met.

12. ETHICS

12.1. Institutional Review Board (IRB)/Independent Ethics Committee (IEC)

The IRB/IEC of London School of Hygiene and Tropical Medicine (LSHTM) is planned to be used in this study in addition to local IRB/IEC. Study related documentation will be reviewed by the LSHTM IRB/IEC and local IRB. No subjects will be enrolled in the study until the approvals from both LSHTM IRB/IEC and local IRB/IEC are received. It is the responsibility of the investigator to have prospective approval of the study protocol, protocol amendments, informed consent forms, and other relevant documents, eg, recruitment advertisements, if applicable, from the IRB/IEC. All correspondence with the IRB/IEC should be retained in the Investigator File. Copies of IRB/IEC approvals should be forwarded to Pfizer.

090177e1811dafca\Approved\Approved On: 22-Feb-2010 14:41

The only circumstance in which an amendment may be initiated prior to IRB/IEC approval is where the change is necessary to eliminate apparent immediate hazards to the subjects. In that event, the investigator must notify the IRB/IEC and Pfizer in writing immediately after the implementation.

12.2. Ethical Conduct of the Study

The study will be conducted in accordance with the Declaration of Helsinki on Ethical Principles for Medical Research Involving Human Subjects, adopted by the General Assembly of the World Medical Association (1996).

In addition, the study will be conducted in accordance with the protocol, the International Conference on Harmonization guideline on Good Clinical Practice, and applicable local regulatory requirements and laws.

12.3. Subject Information and Consent/Assent

All parties will ensure protection of subject personal data and will not include subject names on any sponsor forms, reports, publications, or in any other disclosures, except where required by laws. In case of data transfer, Pfizer will maintain high standards of confidentiality and protection of subject personal data.

The informed consent and assent form must be in compliance with ICH GCP, local regulatory requirements, and legal requirements. Assent will be obtained from subjects <18 years of age. Local IECs/IRBs will determine the age at which assent must be obtained from the subject.

The informed consent and assent form used in this study, and any changes made during the course of the study, must be prospectively approved by both the IRB/IEC and Pfizer before use.

The investigator must ensure that each study subject, or his/her legally acceptable representative, is fully informed about the nature and objectives of the study and possible risks associated with participation. The investigator, or a person designated by the investigator, will obtain written informed consent from each subject or the subject's legally acceptable representative before any study-specific activity is performed. The investigator will retain the original of each subject's signed consent form.

12.4. Reporting of Safety Issues and Serious Breaches of the Protocol or ICH GCP

In the event of any prohibition or restriction imposed (ie, clinical hold) by an applicable Competent Authority in any area of the World, or if the investigator is aware of any new information which might influence the evaluation of the benefits and risks of the investigational product, Pfizer should be informed immediately.

090177e1811dafca\Approved\Approved On: 22-Feb-2010 14:41

In addition, the investigator will inform Pfizer immediately of any urgent safety measures taken by the investigator to protect the study subjects against any immediate hazard, and of any serious breaches of this protocol or of ICH GCP that the investigator becomes aware of.

13. DEFINITION OF END OF TRIAL

13.1. End of Trial in all Participating Countries

End of Trial in all participating countries is defined as Last Subject Last Visit which may have been determined by the point at which the dose escalation and study stopping rules have been met.

14. SPONSOR DISCONTINUATION CRITERIA

Premature termination of this study may occur because of a regulatory authority decision, change in opinion of the IRB/IEC, drug safety problems, or at the discretion of Pfizer. In addition, Pfizer retains the right to discontinue development of AZCQ at any time.

If a study is prematurely terminated or discontinued, Pfizer will promptly notify the investigator. After notification, the investigator must contact all participating subjects and the hospital pharmacy (if applicable) within 30 days. As directed by Pfizer, all study materials must be collected and all CRFs completed to the greatest extent possible.

15. PUBLICATION OF STUDY RESULTS

Publication of study results is discussed in the Clinical Study Agreement.

15.1. Communication of Results by Pfizer

Pfizer fulfils its commitment to publicly disclose the results of studies through posting the results of this study on ClinicalStudyResults.org. Pfizer posts the results of studies that fall into either of the following categories:

- Studies that Pfizer registered on www.clinicaltrials.gov, (ClinicalTrials.gov) regardless of the reason for registration; OR
- All other studies for which the results have scientific or medical importance as determined by Pfizer.

For studies involving a Pfizer product, the timing of the posting depends on whether the Pfizer product is approved for marketing in any country at the time the study is completed:

- For studies involving products already approved in any country and for studies that do not involve a Pfizer product, Pfizer posts results within one year after study completion, defined as Last Subject, Last Visit (LSLV);

090177e1811dafca\Approved\Approved On: 22-Feb-2010 14:41

- For studies involving products that are not yet approved in any country, Pfizer posts the results of already-completed studies within one year after the first regulatory approval of the product;

- For studies involving products whose drug development is discontinued before approval, Pfizer posts the results within one year after such discontinuation.

Pfizer's posting on ClinicalStudyResults.org includes the following elements:

- Protocol title, study phase, and indication;

- A link to approved product labeling, if applicable;

- The synopsis of study results;

- Citations of known study publications;

- Legal disclaimer.

The study results synopsis posted on ClinicalStudyResults.org (called the PhRMA website synopsis) uses the format established by the ICH-E3 Clinical Study Report (CSR) Synopsis. If posting of study results to ClinicalStudyResults.org jeopardizes a planned publication of the study results, a Pending Full Publication notice is substituted for the synopsis until the study results publication has issued or two years have elapsed, whichever occurs first.

Pfizer posts citations only for publications that are accessible in recognized (searchable) publication databases. Single-centre results publications for a multi-centre study are generally not posted because they may not accurately reflect the results of the study.

15.2. Publications by Investigators

Pfizer has no objection to publication by Investigator of any information collected or generated by Investigator, whether or not the results are favorable to the Investigational Drug. However, to ensure against inadvertent disclosure of Confidential Information or unprotected Inventions, Investigator will provide Pfizer an opportunity to review any proposed publication or other type of disclosure before it is submitted or otherwise disclosed.

Investigator will provide manuscripts, abstracts, or the full text of any other intended disclosure (poster presentation, invited speaker or guest lecturer presentation, etc) to Pfizer at least 30 days before they are submitted for publication or otherwise disclosed. If any patent action is required to protect intellectual property rights, Investigator agrees to delay the disclosure for a period not to exceed an additional 60 days.

Investigator will, on request, remove any previously undisclosed Confidential Information (other than the Study results themselves) before disclosure.

090177e1811dafca\Approved\Approved On: 22-Feb-2010 14:41

If the Study is part of a multi-centre study, Investigator agrees that the first publication is to be a joint publication covering all centers. However, if a joint manuscript has not been submitted for publication within 12 months of completion or termination of the Study at all participating sites, Investigator is free to publish separately, subject to the other requirements of this Section.

For all publications relating to the Study, Institution will comply with recognized ethical standards concerning publications and authorship, including Section II - "Ethical Considerations in the Conduct and Reporting of Research" of the Uniform Requirements for Manuscripts Submitted to Biomedical Journals, http://www.icmje.org/index.html#authorship, established by the International Committee of Medical Journal Editors.

Publication of study results is also provided for in the Clinical Study Agreement between Pfizer and the institution. In this section entitled Publications by Investigators, the defined terms shall have the meanings given to them in the Clinical Study Agreement.

090177e1811dafca\Approved\Approved On: 22-Feb-2010 14:41

16. REFERENCES

1. Winstanley, P., *Modern chemotherapeutic options for malaria.* The Lancet Infectious Diseases, 2001. 1(4): p. 242-250.

2. Greenwood, B. and T. Mutabingwa, *Malaria in 2002.* Nature, 2002. 415(6872): p. 670-672.

3. WHO, *WORLD MALARIA REPORT 2009* 2009. http://www.who.int/malaria/publications/atoz/9789241563901/en/index.html.

4. Chico M, e.a., Azithromycin-chloroquine and the intermittent preventive treatment of malaria in pregnancy. Malaria Journal, 2008. 7: p. 255.

5. World Health Organization (WHO). Malaria in Pregnancy. Retrieved on January, 2010 from http://www.who.int/malaria/high_risk_groups/pregnancy/en/index.html.

6. McCormick, M.C., The contribution of low birth weight to infant mortality and childhood morbidity. N Engl J Med, 1985. 312(2): p. 82-90.

7. Ashworth, A., Effects of intrauterine growth retardation on mortality and morbidity in infants and young children. Eur J Clin Nutr, 1998. 52 Suppl 1: p. S34-41; discussion S41-2.

8. Wongsrichanalai, C., et al., Epidemiology of drug-resistant malaria. The Lancet Infectious Diseases, 2002. 2(4): p. 209-218.

9. Barker, D.J., The developmental origins of adult disease. J Am Coll Nutr, 2004. 23(6 Suppl): p. 588S-595S.

10. Godfrey, K.M. and D.J. Barker, Fetal nutrition and adult disease. Am J Clin Nutr, 2000. 71(5 Suppl): p. 1344S-52S.

11. Osmond, C. and D.J. Barker, Fetal, infant, and childhood growth are predictors of coronary heart disease, diabetes, and hypertension in adult men and women. Environ Health Perspect, 2000. 108 Suppl 3: p. 545-53.

12. Eriksson, J.G., et al., Early growth and coronary heart disease in later life: longitudinal study. BMJ, 2001. 322(7292): p. 949-53.

13. Sibai, B., G. Dekker, and M. Kupferminc, Pre-eclampsia. Lancet, 2005. 365(9461): p. 785-99.

14. Villar, J. and J.M. Belizan, The relative contribution of prematurity and fetal growth retardation to low birth weight in developing and developed societies. Am J Obstet Gynecol, 1982. 143(7): p. 793-8.

090177e1811dafca\Approved\Approved On: 22-Feb-2010 14:41

15. WHO, A Strategic Framework for Malaria Prevention and Control During Pregnancy in the Africa Region. Vol. AFR/MAL/04/01. 2004: World Health Organization Regional Office for Africa.

16. Kayentao K, K.M., Newman RD, et al., Comparison of intermittent preventive treatment with chemoprophylaxis for the prevention of malaria during pregnancy in Mali. J Infect Dis, 2005. 191 (1): p. 109-16.

17. WHO, Assessment and Monitoring of antimalarial drug efficacy for the treatment of uncomplicated falciparum malaria. Vol. WHO/HTM/RBM/2003.50. 2003, Geneva: World Health Organization.

18. Roper, C., et al., Antifolate antimalarial resistance in southeast Africa: a population-based analysis. Lancet, 2003. 361(9364): p. 1174-81.

19. Briand, V.r., et al., Intermittent Treatment for the Prevention of Malaria during Pregnancy in Benin: A Randomized, Open Label Equivalence Trial Comparing Sulfadoxine-Pyrimethamine with Mefloquine. The Journal of Infectious Diseases, 2009. 200(6): p. 991-1001.

20. Smoak, B.L., et al., The effects of inadvertent exposure of mefloquine chemoprophylaxis on pregnancy outcomes and infants of US Army servicewomen. J Infect Dis, 1997. 176(3): p. 831-3.

21. Nosten, F., et al., The effects of mefloquine treatment in pregnancy. Clin Infect Dis, 1999. 28(4): p. 808-15.

22. Clerk, C.A., et al., A Randomized, Controlled Trial of Intermittent Preventive Treatment with Sulfadoxine-Pyrimethamine, Amodiaquine, or the Combination in Pregnant Women in Ghana. J Infect Dis, 2008.

23. Tagbor, H., et al., Efficacy, safety, and tolerability of amodiaquine plus sulphadoxine-pyrimethamine used alone or in combination for malaria treatment in pregnancy: a randomised trial. Lancet, 2006. 368(9544): p. 1349-56.

24. Fanello, C.I., et al., Tolerability of amodiaquine and sulphadoxine-pyrimethamine, alone or in combination for the treatment of uncomplicated Plasmodium falciparum malaria in Rwandan adults. Trop Med Int Health, 2006. 11(5): p. 589-96.

25. Vallely A, V.L., Changalucha J, Greenwood B, Chandramohan D Intermittent preventive treatment for malaria in pregnancy in Africa: what's new, what's needed? Malar J, 2007. 6: p. 16.

26. WHO, Assessment of the Safety of Artemisinin Compounds in Pregnancy. Report of two informal consultations convened by WHO in 2002 (Roll Back Malaria and the UNDP/World Bank/WHO Special Programme for Research and Training in Tropical Diseases). Vol. WHO/CDS/MAL/2003.1094. WHO/RBM/TDR/Artemisinin/03.1. 2003, Geneva: World Health Organization [http://www.who.int/malaria/cmc_upload/0/000/016/323/artemisinin_pregnancy.pdf].

090177e1811dafca\Approved\Approved On: 22-Feb-2010 14:41

27. Andersen SL, A.A., McGreevy P, et al. , *Activity of azithromycin as a blood schizonticide against rodent and human plasmodia in vivo.* American Journal of Tropical Medicine & Hygiene 1995. 52: p. 159-61.

28. Sadiq, S.T., et al., Effects of azithromycin on malariometric indices in The Gambia. Lancet, 1995. 346(8979): p. 881-2.

29. Greenwood, B., et al., Malaria in pregnancy: priorities for research. Lancet Infect Dis, 2007. 7(2): p. 169-74.

30. Gray, R.H., et al., Randomized trial of presumptive sexually transmitted disease therapy during pregnancy in Rakai, Uganda. American Journal of Obstetrics and Gynecology, 2001. 185(5): p. 1209-1217.

31. Kaul, R., et al., Monthly antibiotic chemoprophylaxis and incidence of sexually transmitted infections and HIV-1 infection in Kenyan sex workers: a randomized controlled trial. JAMA, 2004. 291(21): p. 2555-62.

32. Morency, A.M. and E. Bujold, The effect of second-trimester antibiotic therapy on the rate of preterm birth. J Obstet Gynaecol Can, 2007. 29(1): p. 35-44.

33. Girard, A.E., et al., Pharmacokinetic and in vivo studies with azithromycin (CP-62,993), a new macrolide with an extended half-life and excellent tissue distribution. Antimicrob Agents Chemother, 1987. 31(12): p. 1948-54.

34. Dunn CJ and Barradell LB, Azithromycin. A review of its pharmacological properties and use as 3-day therapy in respiratory tract infections. Drugs, 1996. 51(3) p. 483-505.

35. Ballow, C.H. and G.W. Amsden, Azithromycin: the first azalide antibiotic. Ann Pharmacother, 1992. 26(10): p. 1253-61.

36. Peters, D.H., H.A. Friedel, and D. McTavish, Azithromycin. A review of its antimicrobial activity, pharmacokinetic properties and clinical efficacy. Drugs, 1992. 44(5): p. 750-99.

37. Sidhu AB, et al., In vitro efficacy, resistance selection, and structural modeling studies implicate the malarial parasite apicoplast as the target of azithromycin. J Biol Chem, 2007. 282(4): p. 2494-504.

38. WHO, Sexually transmitted and other reproductive tract infections: Guide to essential practice. Integrating STI/RTI Care for Reproductive Health. 2005, Geneva: Department of Reproductive Health and Research, World Health Organization.

39. Kiddugavu, M.G., et al., Effectiveness of syphilis treatment using azithromycin and/or benzathine penicillin in Rakai, Uganda. Sex Transm Dis, 2005. 32(1): p. 1-6.

090177e1811dafca\Approved\Approved On: 22-Feb-2010 14:41

40. Riedner, G., et al., Single-dose azithromycin versus penicillin G benzathine for the treatment of early syphilis. N Engl J Med, 2005. 353(12): p. 1236-44.

41. Araujo, F.G., D.R. Guptill, and J.S. Remington, Azithromycin, a macrolide antibiotic with potent activity against Toxoplasma gondii. Antimicrob Agents Chemother, 1988. 32(5): p. 755-7.

42. Crouch, A.A., et al., Sensitivity in vitro of Giardia intestinalis to dyadic combinations of azithromycin, doxycycline, mefloquine, tinidazole and furazolidone. Trans R Soc Trop Med Hyg, 1990. 84(2): p. 246-8.

43. Ravdin, J.I. and J. Skilogiannis, In vitro susceptibilities of Entamoeba histolytica to azithromycin, CP-63,956, erythromycin, and metronidazole. Antimicrob Agents Chemother, 1989. 33(6): p. 960-2.

44. Krause, P.J., et al., Atovaquone and azithromycin for the treatment of babesiosis. N Engl J Med, 2000. 343(20): p. 1454-8.

45. Noedl H, W.W., Krudsood S, et al,. Antimalarial activity of azithromycin, artemisinin and dihydroartemisinin in fresh isolates of Plasmodium falciparum in Thailand. Acta Tropica 2001. 80: p. 39-44.

46. Sidhu ABS, et al., Identification of a novel mutation in the L4 plastid ribosomal protein in P. falciparum azithromycin-resistant lines, in 54th Annual Meeting of the American Society of Topical Medicine and Hygiene. 2005, Albert Einstein College of Medicine and Pfizer Inc: Washington DC.

47. Gingras, B. and J. Jensen, Activity of azithromycin or erythromycin in combination with antimalarial drugs against multidrug-resistant Plasmodium falciparum in vitro. Am J Trop Med. Hygiene, 1992. 47: p. 378-382.

48. Gingras, B. and J. Jensen, Antimalarial activity of azithromycin and erythromycin against Plasmodium burghei. Am J Trop Med, 1993. 49: p. 101-105.

49. Yeo AE, R.K., Increased antimalarial activity of azithromycin during prolonged exposure of Plasmodium falciparum in vitro. Int J Parasitol 1995. 25: p. 531-2.

50. Biswas, S., In vitro antimalarial activity of azithromycin against chloroquine sensitive and chloroquine resistant Plasmodium falciparum. Journal of Postgraduate Medicine, 2001. 47 p. 240-3.

51. Duff, P., Antibiotic selection in obstetric patients. Infect Dis Clin North Am, 1997. 11(1): p. 1-12.

52. Ogasawara, K.K. and T.M. Goodwin, Efficacy of azithromycin in reducing lower genital Ureaplasma urealyticum colonization in women at risk for preterm delivery. J Matern Fetal Med, 1999. 8(1): p. 12-6.

090177e1811dafca\Approved\Approved On: 22-Feb-2010 14:41

53. Donders, G.G., Treatment of sexually transmitted bacterial diseases in pregnant women. Drugs, 2000. 59(3): p. 477-85.

54. Ramsey, P.S., et al., Maternal and transplacental pharmacokinetics of azithromycin. Am J Obstet Gynecol, 2003. 188(3): p. 714-8.

55. Heikkinen, T., et al., The transplacental transfer of the macrolide antibiotics erythromycin, roxithromycin and azithromycin. BJOG, 2000. 107(6): p. 770-5.

56. Liu, P., et al., Comparative Pharmacokinetics of Azithromycin in Serum and White Blood Cells of Healthy Subjects Receiving a Single-Dose Extended-Release Regimen versus a 3-Day Immediate-Release Regimen. Antimicrob. Agents Chemother., 2007. 51(1): p. 103-109.

57. Taylor WR, et al., Malaria prophylaxis using azithromycin: a double-blind, placebo-controlled trial in Irian Jaya, Indonesia. Clin Infect Dis 1999. 28: p. 74–81.

58. Heppner, D.G., Jr., et al., Randomized, controlled, double-blind trial of daily oral azithromycin in adults for the prophylaxis of Plasmodium vivax malaria in Western Thailand. Am J Trop Med Hyg, 2005. 73(5): p. 842-9.

59. Andersen, S.L., et al., Successful double-blinded, randomized, placebo-controlled field trial of azithromycin and doxycycline as prophylaxis for malaria in western Kenya. Clin Infect Dis, 1998. 26(1): p. 146-50.

60. Kuschner, R., et al., Azithromycin prophylaxis against a chloroquine resistant strain of Plasmodium falciparum. Lancet, 1994. 343(8910): p. 1396–1397.

61. Anderson, S., et al., Prophylaxis of Plasmodium falciparum malaria with azithromycin administered to volunteers. Ann Intern Med, 1995. 123: p. 771–773.

62. Dunne, MichaelÂ W., et al., A Multicenter Study of Azithromycin, Alone and in Combination with Chloroquine, for the Treatment of Acute Uncomplicated Plasmodium falciparum Malaria in India. The Journal of Infectious Diseases, 2005. 191(10): p. 1582-1588.

63. Mullick, S., et al., Sexually transmitted infections in pregnancy: prevalence, impact on pregnancy outcomes, and approach to treatment in developing countries. Sex Transm Infect, 2005. 81(4): p. 294 - 302.

64. Gray, R.H., et al., Randomized trial of presumptive sexually transmitted disease therapy during pregnancy in Rakai, Uganda. Am J Obstet Gynecol, 2001. 185(5): p. 1209-1217.

65. Who, Sexually transmitted and other reproductive tract infections: Guide to essential practice. 2005.

090177e1811dafca\Approved\Approved On: 22-Feb-2010 14:41

66. CDC, Sexually Transmitted Diseases Treatment Guidelines, 2006. MMWR, 2006. 55 (No. RR-11).

67. Riedner, G., et al., Single-dose azithromycin versus penicillin G benzathine for the treatment of early syphilis. N Engl J Med, 2005. 353(12): p. 1236-1244.

68. Hook E, et al (2008). National STD Prevention Conference 2008. Chicago, Ill. LBSb.

69. Pfizer, Trovano®/Zithromax® Compliance Pak. 1998: [http://www.fda.gov/CDER/foi/label/1998/50-762.pdf].

70. Pitsouni, E., et al., Single-dose azithromycin versus erythromycin or amoxicillin for Chlamydia trachomatis infection during pregnancy: a meta-analysis of randomised controlled trials. Int J Antimicrob Agents, 2007. 30(3): p. 213-21.

71. Bar-Oz B, Diav-Citrin, O, Schechtman, S, et al. Pregnancy outcome after gestational exposure to the new macrolides: a prospective multi-center observational study. European J of Obstetrics and Gynecology and Reproductive Biology 2008; 141:31-34.

72. Cooper W, Hernandez-Diaz, S, Arbogast PG, et al. Antibiotics potentially used in response to bioterrorism and the risk of major congenital malformations. Pediatric and Perinatal Epidemiology 2008; 23:18-28.

73. Kalliani L, Mofolo I, Chaponda M, et al. A randomized controlled pilot trial of azithromycin or astesunate added to sulfadoxine-pryimethamine as treatment for malaria in pregnant women. PLoS ONE. Vol. 2, 2007; e1166. doi:10.1371/journal.pone.0001166.

74. Pihlajamaki, M., J. Kataja, H. Seppala, J. Elliot, M. Leinonen, P. Huovinen, and J. Jalava. 2002. Ribosomal mutations in Streptococcus pneumoniae clinical isolates. Antimicrob. Agents Chemother. 46:654–658.

75. Leach AJ, Shelby-James TM, Mayo M, Gratten M, Laming AC, Currie BJ, Mathews JD: A prospective study of the impact of community-based azithromycin treatment of trachoma on carriage and resistance of Streptococcus pneumoniae. Clin Infect Dis 1997, 24(3):356-362.

76. Gaynor BD, Holbrook KA, Whitcher JP, Holm SO, Jha HC, Chaudhary JS, Bhatta RC, Lietman T: Community treatment with azithromycin for trachoma is not associated with antibiotic resistance in Streptococcus pneumoniae at 1 year. Br J Ophthalmol 2003, 87(2):147-148.

77. Batt SL, Charalambous BM, Solomon AW, Knirsch C, Massae PA, Safari S, Sam NE, Everett D, Mabey DC, Gillespie SH: Impact of AzithromycinAdministration for Trachoma Control on the Carriage of Antibiotic-Resistant Streptococcus pneumoniae. ANTIMICROBIAL AGENTS AND CHEMOTHERAPY. 2003, 47(9):2765–2769.

090177e1811dafca\Approved\Approved On: 22-Feb-2010 14:41

78. Sidhu, A.B., D. Verdier-Pinard, and D.A. Fidock, Chloroquine resistance in Plasmodium falciparum malaria parasites conferred by pfcrt mutations. Science, 2002. 298(5591): p. 210-3.

79. Fidock DA, et al., Mutations in the P. falciparum digestive vacuole transmembrane protein PfCRT and evidence for their role in chloroquine resistance. Mol Cell, 2000. 6(4): p. 861-71.

80. Reprotox. Website accessed electronically on May, 2006 from http://csi.micromedex.com/DATA/RX/RX1056.HTM?Top=Yes.

81. Udalova et al, from Shepard's Catalog of Teratogenic Agents. Website accessed electronically on May, 2006 from http://csi.micromedex.com/DATA/SH/SH660.HTM?Top=Yes.

82. Walker, B. and Warner, C. (1974). Proceedings: A preliminary investigation of the teratogenic action of chloroquine in the rat. West African J Pharmacol Drug Res 2: 61P-62P.

83. Sharma, A. and Rawat, A.K.(1989) Toxicological Consequences of Chloroquine and Ethanol on the Developing Fetus. Pharmacology Biochemistry & Behavior 34: 77-82.

84. Lee, S., et al., Chloroquine pharmacokinetics in pregnant and nonpregnant women with vivax malaria. European Journal of Clinical Pharmacology, 2008. 64(10): p. 987-992.

85. Denoeud, L., et al., Is chloroquine chemoprophylaxis still effective to prevent low birth weight? Results of a study in Benin. Malar J, 2007. 6: p. 27.

86. Mita, T., et al., Recovery of chloroquine sensitivity and low prevalence of the Plasmodium falciparum chloroquine resistance transporter gene mutation K76T following the discontinuance of chloroquine use in Malawi. Am J Trop Med Hyg, 2003. 68(4): p. 413-5.

87. Takechi, M., et al., Therapeutic efficacy of sulphadoxine/pyrimethamine and susceptibility in vitro of P. falciparum isolates to sulphadoxine-pyremethamine and other antimalarial drugs in Malawian children. Trop Med Int Health, 2001. 6(6): p. 429-34.

88. Djimde, A., et al., A molecular marker for chloroquine-resistant falciparum malaria. N Engl J Med, 2001. 344(4): p. 257-63.

89. Kublin, J.G., et al., Reemergence of chloroquine-sensitive Plasmodium falciparum malaria after cessation of chloroquine use in Malawi. J Infect Dis, 2003. 187(12): p. 1870-5.

90. Laufer, M.K., et al., Return of chloroquine antimalarial efficacy in Malawi. N Engl J Med, 2006. 355(19): p. 1959-66.

090177e1811dafca\Approved\Approved On: 22-Feb-2010 14:41

91. Hastings, I.M. and M.J. Donnelly, The impact of antimalarial drug resistance mutations on parasite fitness, and its implications for the evolution of resistance. Drug Resist Updat, 2005. 8(1-2): p. 43-50.

92. Laufer, M.K. and C.V. Plowe, Withdrawing antimalarial drugs: impact on parasite resistance and implications for malaria treatment policies. Drug Resist Updat, 2004. 7(4-5): p. 279-88.

93. Walliker, D., P. Hunt, and H. Babiker, Fitness of drug-resistant malaria parasites. Acta Trop, 2005. 94(3): p. 251-9.

94. Mwai, L., et al., Chloroquine resistance before and after its withdrawal in Kenya. Malar J, 2009. 8: p. 106.

95. Neely, M., et al., Effect of chloroquine on human immunodeficiency virus (HIV) vertical transmission. Afr Health Sci, 2003. 3(2): p. 61-7.

96. Semrau, K., et al., Impact of chloroquine on viral load in breast milk. Trop Med Int Health, 2006. 11(6): p. 800-3.

97. Whitehouse, M.W. and H. Bostrom, Biochemical properties of anti-inflammatory drugs. VI. The effects of chloroquine (resochin), mepacrine (quinacrine) and some of their potential metabolites on cartilage metabolism and oxidative phosphorylation. Biochem Pharmacol, 1965. 14(8): p. 1173-84.

98. Anigbogu, C.N., et al., Chloroquine reduces blood pressure and forearm vascular resistance and increases forearm blood flow in healthy young adults. Clin Physiol, 1993. 13(2): p. 209-16.

99. Jaeger, A., Poisonous substances: Quinine and chloroquine. Medicine, 2007. 35: p. 652-653.

100. Sowunmi, A., O. Walker, and L.A. Salako, Pruritus and antimalarial drugs in Africans. Lancet, 1989. 2(8656): p. 213.

101. Mutabingwa, T., L. Villegas, and F. Nosten, Chemoprophylaxis and other protective measures: Preventing pregnancy malaria, in Malaria in Pregnancy: Deadly Parasite, Susceptible Host, P. Duffy and M. Fried, Editors. 2001, Taylor and Francis: London. p. 189-222.

102. Tagbor H, Bruce J, Ord R, Randall A, et al. Comparison of the therapeutic efficacy of chloroquine and sulphadoxine-pyremethamine in children and pregnant women. Tropical Medicine and International Health 2007; 12:1288-1297.

103. Briand V, Denoeund L, Massougbodji, A, et al. Efficacy of intermittent preventive treatment versus chloroquine prophylaxis to prevent malaria during pregnancy in Benin. Journal of Infectious Diseases 2008; 198:594-601.

090177e1811dafca\Approved\Approved On: 22-Feb-2010 14:41

104. Asa O, Onayade, AA, Fatusi, AO. Efficacy of intermittent preventive treatment of malaria with sulphadoxine-pyrimethamine in preventing anaemia in pregnancy among Nigerian women. Matern Child Health J 2008; 12:692-698.

105. Law I, Ilett KF, Hackett, P, et al. Transfer of chloroquine and desethylcholorquine across the placenta and into milk in Melanesian mothers. British J of Clinical Pharmacology 1008; 65:674-679.

106. Ohrt, C., et al., Assessment of azithromycin in combination with other antimalarial drugs against Plasmodium falciparum in vitro. Antimicrob Agents Chemother, 2002. 46(8): p. 2518-24.

107. Salman, S., S. J. Rogerson, et al. (2009). The pharmacokinetic properties of azithromycin in pregnancy. Antimicrob. Agents Chemother: AAC.00771-09.

108. Chandra R, L.D., Moran D, Dubhashi N, Sarkar S, Wang C, Cai J, Dunne M, A phase 2, open label, non-comparative trial of Azithromycin 2g plus chloroquine 600 mg base daily for three days for the treatment of uncomplicated Plasmodium falciparum malaria. 57th ASTMH (American Society of Tropical Medicine and Hygiene) annual meeting, New Orleans, USA, 2008.

109. Lewis, D., et al., A Phase 2/3, Randomized, Double Blind, Comparative Trial of Azithromycin Plus Chloroquine Versus Mefloquine for the Treatment of Uncomplicated Plasmodium falciparum Malaria in Africa. , in 5th European Congress on Tropical Medicine and International Health. 2007: Amsterdam.

110. Chandra R, et al., A Phase 3, Randomized, Open-Label, Comparative Trial of Azithromycin plus Chloroquine versus Mefloquine for the Treatment of Uncomplicated Plasmodium falciparum Malaria in Africa, in 56th Annual Meeting of the American Society of Topical Medicine and Hygiene. 2007, Pfizer Inc: Philadelphia.

111. Most H, Clinical Trials of Antimalarial Drugs, in Internal Medicine in World War II, Coates JB, Editor. 1963, Office of the Surgeon General, Medical Department, United States Army: Washington DC.

112. ter Kuile, F.O., A.M. van Eijk, and S.J. Filler, Effect of sulfadoxine-pyrimethamine resistance on the efficacy of intermittent preventive therapy for malaria control during pregnancy: a systematic review. JAMA, 2007. 297(23): p. 2603-16.

113. Tagbor, H., et al., Comparison of the therapeutic efficacy of chloroquine and sulphadoxine-pyrimethamine in children and pregnant women. Trop Med Int Health, 2007. 12(11): p. 1288-97.

114. WHO, DEFINITIONS AND INDICATORS IN FAMILY PLANNING MATERNAL & CHILD HEALTH AND REPRODUCTIVE HEALTH. Reproductive, Maternal and Child Health European Regional Office, 2001.

090177e1811dafca\Approved\Approved On: 22-Feb-2010 14:41

115. Agresti, A., Categorical Data Analysis. 2nd Edition. New York: John Wiley & Sons, Inc. 2002.

116. Mantel, N., Haenszel, W, Statistical Aspects of the Analysis of Data from Retrospective Studies of Disease. J of the National Cancer Institute, 1959. 22: p. 719-748.

117. Greenland, S., Robins, JM. Estimators of the Mantel-Haenszel Variance Consistent in Both Sparse Data and Large-Strata Limiting Models. Biometrics, 1985. 42: p. 311-323.

118. Gaynor BD, Chidambaram JD, Cevallos V, Miao Y, Miller K, Jha HC, Bhatta RC, Chaudhary JS, Osaki Holm S, Whitcher JP et al: Topical ocular antibiotics induce bacterial resistance at extraocular sites. Br J Ophthalmol 2005, 89(9):1097-1099.

119. Chern KC, Shrestha SK, Cevallos V, Dhami HL, Tiwari P, Chern L, Whitcher JP, Lietman TM: Alterations in the conjunctival bacterial flora following a single dose of azithromycin in a trachoma endemic area. Br J Ophthalmol 1999, 83(12):1332-1335.

120. Hoepelmana I.M., Schneider M.M.E. Azithromycin: the first of the tissue-selective azalides. International Journal of Antimicrobial Agents. 1995(5): 145-167.

121. G. Foulds, R.A. Ferraina, H. G. Fouda, R. B. Johnson, A. M. Kamel, and R. M. Shepard, (1995) Azithromycin Concentrations in Gallbladder, hepatic tissue, and bile following a 5-day regimen in humans. Infectious disease and therapy (1995), 18:367-372.

090177e1811dafca\Approved\Approved On: 22-Feb-2010 14:41
